# Characterization of post-mitotic DNA synthesis and its regulation in human cells

**DOI:** 10.1038/s41467-026-78035-5

**Published:** 2026-09-28

**Authors:** Julia Vornberger, Ralph Imhof, Virginia Pasti, Beatrice Bianchi, Mingxuan Wei, Jeannette Alisha Straw, Sandhya Manohar, Matthias Altmeyer

**Affiliations:** 1 Department of Molecular Mechanisms of Disease, University of Zurich (UZH), Zurich, Switzerland; 2 Molecular Life Sciences PhD Program, Life Science Zurich Graduate School, Zurich, Switzerland

**Keywords:** Cell biology, DNA damage and repair, DNA replication

## Abstract

DNA replication stress promotes cancer development and is associated with DNA synthesis beyond S-phase. Replication stress-induced DNA lesions and under-replicated DNA can persist into G2 and mitosis, where they undergo mitotic DNA synthesis (MiDAS). After cell division, inherited DNA lesions are protected by genome caretaker proteins and show signs of post-mitotic DNA synthesis (post-MiDAS) in G1. The genomic regions that undergo post-MiDAS, the cellular factors involved, and the implications for genome integrity remain poorly understood. Here, we provide evidence for post-MiDAS activity at centromeres and telomeres in cancer cells employing alternative lengthening of telomeres (ALT) and present data suggesting that a subset of these fragile genomic regions can cluster during post-MiDAS in G1. We further show that post-MiDAS is promoted by local SUMOylation and BLM recruitment into ALT-associated PML bodies (APBs) and involves DNA damage tolerance factors and proteins associated with ALT and MiDAS. Consistent with G1-specific regulation, complementary quantitative proteomics and targeted siRNA screening reveal that APC/C, together with its G1 co-activator CDH1 but independent of its mitotic co-activator CDC20, restrains excessive post-MiDAS in G1. Finally, we show that CDH1 depletion selectively impairs the survival of ALT-positive cancer cell lines, indicating that post-MiDAS might represent a potential ALT-specific vulnerability.

## Abstract (web summary)

Mitotic DNA synthesis (MiDAS) is a response to replication stress. Here, the authors characterize a related process, post-MiDAS, in G1-phase cells that had completed mitosis. They reveal that post-MiDAS occurs at telomeres and centromeres and identify key factors involved in this process.

## Introduction

Progressive telomere shortening during cell division limits cellular proliferation^1–4^. To achieve replicative immortality, cancer cells either reactivate the reverse transcriptase telomerase to extend their telomeres, or use the alternative lengthening of telomeres (ALT) pathway for telomere maintenance^5–10^. ALT is prevalent in cancers of predominantly mesenchymal origin, including osteosarcoma, chondrosarcoma, and leiomyosarcoma, and in tumors of the central nervous system, including glioma and astrocytoma, and is associated with limited therapeutic options and poor prognosis^11–13^. Different from telomerase-mediated telomere extension, ALT is a recombination-based mechanism, the molecular details and regulation of which remain poorly understood.

Telomeres and subtelomeric regions, composed of long heterochromatic tandem sequence repeats, are inherently difficult to replicate^14,15^. Replication stress at telomeres is particularly high in ALT-positive cancer cells due to the loss of chromatin regulators such as ATRX and DAXX, elevated levels of telomeric repeat-containing RNA (TERRA), and increased formation of secondary structures such as G-quadruplexes and R-loops^16–25^. Persistent replication fork stalling during telomere replication results in replication fork collapse, which, through the generation of one-ended DNA double-strand breaks (DSBs), can initiate break-induced DNA replication (BIR)^26,27^. Mechanistically, ALT is thought to employ RAD52 and POLD3 for conservative DNA replication using a homologous template and to occur within ALT-associated promyelocytic leukemia (PML) bodies (APBs)^27–30^. Whereas ALT is initiated primarily in S-phase, it can extend into the G2-phase of the cell cycle to resolve ALT replication intermediates that would otherwise threaten genome stability^31^.

A subset of replication intermediates, including regions with under-replicated DNA, can escape cell cycle checkpoint surveillance and persist into mitosis^32,33^. To preserve genome integrity, cells attempt to complete DNA replication at these sites in early mitosis through mitotic DNA synthesis (MiDAS)^33–35^. Like ALT, MiDAS is thought to proceed via a BIR-like mechanism and to serve as a last resort pathway to resolve under-replicated DNA before chromosome segregation^34–37^. Accordingly, MiDAS has emerged as a safeguard of genome integrity, particularly for difficult-to-replicate loci such as telomeres.

Nonetheless, not all replication intermediates can be fully resolved before sister chromatids separate, and the remaining unresolved genomic lesions are transmitted to later stages of mitosis and, upon processing, to the next cell generation. In G1-phase cells, inherited genomic regions with under-replicated DNA or unresolved DNA lesions are sequestered in nuclear compartments, referred to as 53 binding protein 1 (53BP1) nuclear bodies, where they are protected from nucleolytic degradation to avoid chromosome attrition^38,39^.

Recent work revealed that the heterotrimeric single-stranded DNA (ssDNA)-binding replication protein A (RPA) complex marks replication stress-induced heritable DNA lesions in G1 cells, which are largely distinct from 53BP1-marked genomic regions^40^. The binding of RPA at these ssDNA lesions is associated with nascent DNA synthesis in G1, referred to as post-mitotic DNA synthesis (post-MiDAS)^40^. Post-MiDAS in G1 occurs in normally proliferating, unperturbed cells and is elevated when replication stress is experienced in the previous cell cycle. Telomeres are hotspots for post-MiDAS, especially in ALT-positive cancer cells, indicating that this process may serve as a backup mechanism for failed or incomplete telomere synthesis in S/G2 and mitosis, thereby further extending the concept of unscheduled DNA synthesis to the next cell generation^40^. In support of this notion, budding yeast completes DNA synthesis at difficult-to-replicate regions near chromosome ends late in mitosis and in early G1^41^. Unscheduled DNA synthesis in G1 must be tightly controlled and has been associated with elevated mutation rates and genome instability^41,42^. However, the extent to which post-MiDAS promotes or challenges genome integrity is unclear. Moreover, the cellular factors and molecular mechanisms involved in post-MiDAS are largely undefined, and how this process is regulated in G1 remains to be elucidated. Here, we sought to characterize post-MiDAS at RPA-marked fragile genomic regions in unchallenged conditions and in response to replication stress, elucidate the molecular mechanisms, and identify cellular factors involved in this process.

## Results

### Post-MiDAS occurs at ALT telomeres in unchallenged cells and upon replication stress

**Fig. 1:**
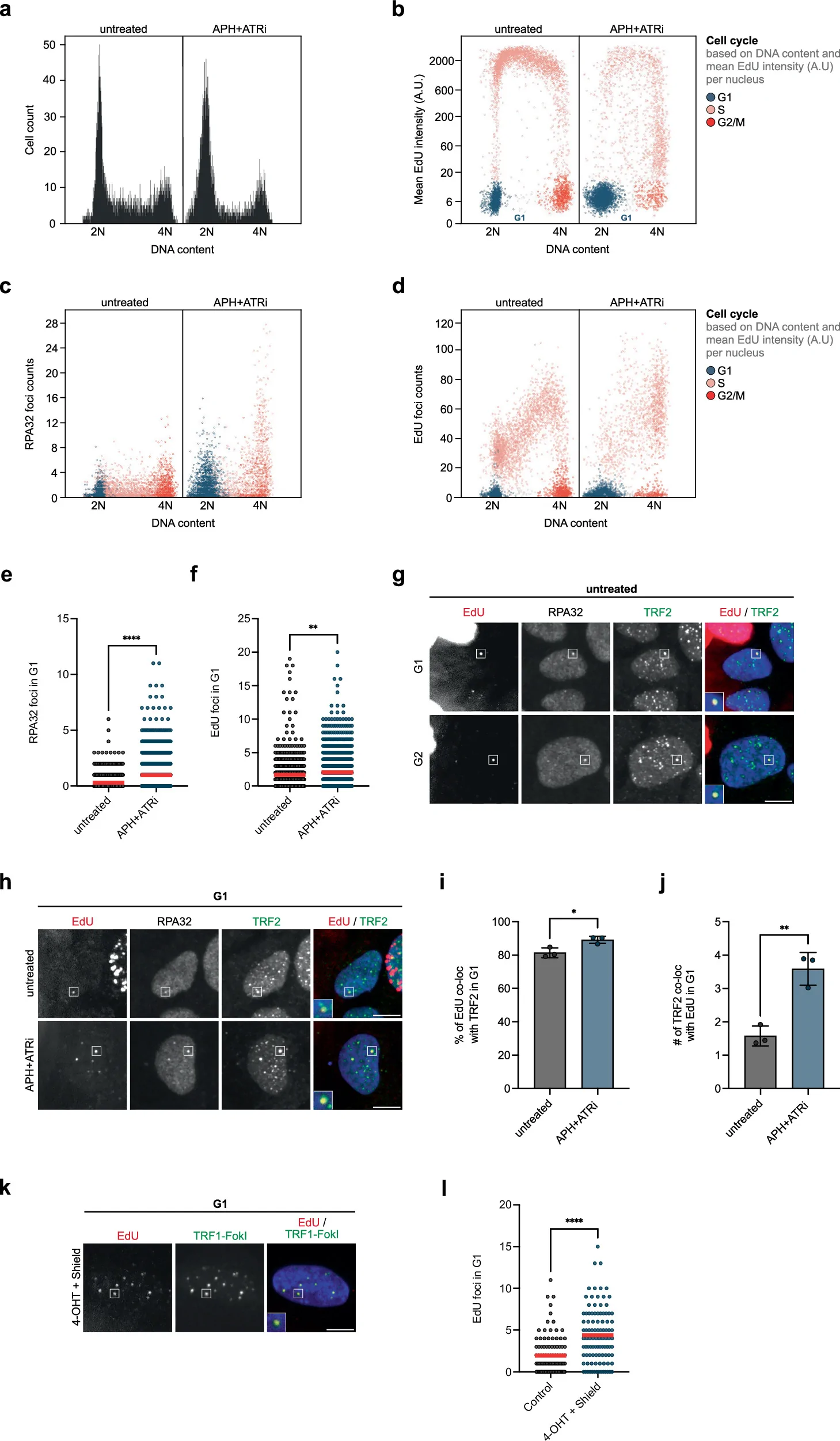
Post-MiDAS occurs at telomeres in ALT-positive cells. **a** High-content microscopy-derived cell cycle profiles based on DNA content (total DAPI intensity (A.U.)) per U-2 OS cell nucleus. Cells were untreated or treated with APH (0.2 µM) and ATRi (1 µM) for 24 h (*n* = 5000 cells per condition). **b** Cell cycle profiles based on total DAPI intensity (A.U.) and EdU incorporation (mean EdU intensity (A.U.)) per U-2 OS nucleus as in (**a**). Color coding indicates the cell cycle phase defined by DNA content and mean EdU intensity (*n* = 5000 cells per condition). **c**,** d** Cell cycle-resolved RPA32 (**c**) and EdU (**d**) foci formation per U-2 OS nucleus as in (**a**). Color coding indicates the cell cycle phase as defined in (**b**) (*n* = 5000 cells per condition). **e**,** f** RPA32 (**e**) and EdU (**f**) foci counts per G1-phase U-2 OS nucleus as in (**a**). Scatterplots show *n* = 1000 cells per condition. Red lines represent the mean. **** *p* < 0.0001 (**e**) and ** *p* = 0.0015 (**f**) (two-tailed unpaired *t* test). **g** Representative images of EdU, RPA32, and TRF2 foci co-localization in untreated G1- and G2-phase U-2 OS cells. **h** Representative images of EdU, RPA32, and TRF2 foci co-localization in G1-phase U-2 OS cells, as in (**a**). **i**,** j** Percentage of EdU foci co-localizing with TRF2 (**i**) and number of TRF2 foci co-localizing with EdU (**j**) in G1-phase U-2 OS cells as in (**a**). Means ± SD of three independent experiments are shown (*n* = 100 cells per experiment). * *p* = 0.0209 (**i**) and ** *p* = 0.0038 (**j**) (two-tailed unpaired *t* test). **k** Representative images of EdU and TRF1-FokI foci co-localization in G1-phase U-2 OS TRF1-FokI cells treated with 4-OHT (1 µM) and Shield-1 (1 µM) for 2 h. **l** EdU foci count per G1-phase U-2 OS TRF1-FokI nucleus as in (**k**). Scatterplots show *n* = 100 cells per condition. Red lines represent the mean. **** *p* < 0.0001 (two-tailed unpaired *t* test). A.U., arbitrary units. Scale bars, 10 µm. Source data are provided as a Source Data file.

To validate and extend previous findings that ALT-dependent cancer cells are particularly prone to inherit RPA-marked ssDNA lesions from the previous cell cycle and use post-MiDAS in G1^40^, we analyzed post-MiDAS events in ALT-positive U-2 OS cells by quantitative image-based cytometry (QIBC)^43,44^. These analyses included both unperturbed cell populations as well as cells with elevated replication stress upon treatment with a low dose of the DNA polymerase inhibitor aphidicolin (APH) and an ATR inhibitor (ATRi) to override the G2/M checkpoint and thereby increase the number of cells in G1 with replication stress-induced DNA lesions (Supplementary Fig. 1a, b). QIBC enabled cell cycle staging and identification of G1 cells in asynchronous cell populations (Fig. 1a, b and Supplementary Fig. 1a, b) and allowed us to quantify RPA and EdU (5-ethynyl-2’-deoxyuridine) foci, marking DNA synthesis, in specific cell cycle phases (Fig. 1c, d and Supplementary Fig. 1c, d). As before^40^, EdU pulse labeling for 1 h in asynchronous cell populations was performed in the presence of nocodazole to prevent mitotic cells from entering G1 during the period of the EdU pulse. Reliable identification of G1 cells by QIBC was validated in U-2 OS cells stably expressing established fluorescent ubiquitylation-based cell cycle indicators (FUCCI) based on GFP-Geminin (amino acids 1–110) and RFP-CDT1 (amino acids 30-120) fragments^45^, analyzed for DNA content and additionally either EdU incorporation or Cyclin A expression (Supplementary Fig. 2a, b). Consistent with our previous findings^40^, RPA foci were observed in unchallenged G1 cells, and their numbers increased upon replication stress (Fig. 1e). Likewise, EdU foci were detected in unchallenged G1 cells, and their numbers increased upon replication stress (Fig. 1f). RPA and EdU foci co-localized with the telomere marker telomeric repeat-binding factor 2 (TRF2) in G1 cells, like in G2, indicating that post-MiDAS activity occurs at telomeres (Fig. 1g, h). Around 80–90% of discernable EdU foci in G1, and thus post-MiDAS sites, showed co-localization with TRF2 (Fig. 1i), and elevated replication stress increased the fraction of telomeres undergoing post-MiDAS in G1, consistent with replication stress-induced telomeric DNA lesions (Fig. 1j).

Interestingly, not only replication stress in the previous cell cycle but also targeted induction of DSBs at telomeres by the FokI endonuclease fused to the telomeric repeat-binding factor 1 (TRF1-FokI)^28,46^ enhanced post-MiDAS (Fig. 1k, l and Supplementary Fig. 3a–d). Post-MiDAS at telomeres thus occurs in unchallenged, normally proliferating U-2 OS cells and is enhanced both upon replication stress and upon acute telomere-associated DNA damage.

### Post-MiDAS activity occurs at centromeres

**Fig. 2:**
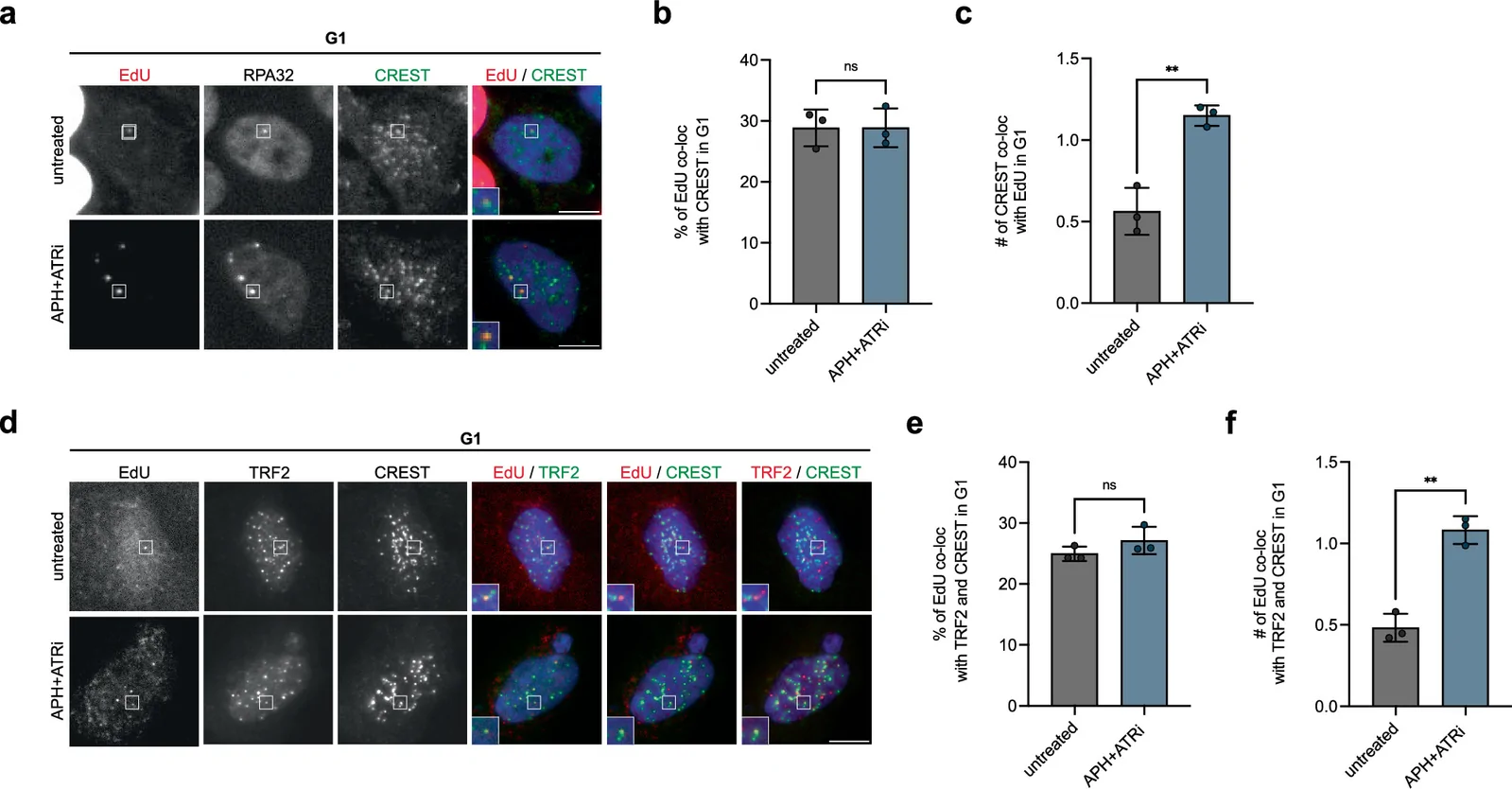
Telomeres and centromeres cluster at post-MiDAS sites. **a** Representative images of EdU, RPA32, and CREST foci co-localization in G1-phase U-2 OS cells. Cells were untreated or treated with APH (0.2 µM) and ATRi (1 µM) for 24 h. **b**,** c** Percentage of EdU foci co-localizing with CREST (**b**) and number of CREST foci co-localizing with EdU (**c**) in G1-phase U-2 OS cells as in (**a**). Means ± SD of three independent experiments are shown (*n* = 100 cells per experiment). ns *p* = 0.9888 (**b**) and ** *p* = 0.0029 (**c**) (two-tailed unpaired *t* test). **d** Representative images of EdU, TRF2, and CREST foci co-localization in G1-phase U-2 OS cells as in (**a**). **e**,** f** Percentage of EdU foci co-localizing with TRF2 and CREST (**e**) and number of EdU foci co-localizing with TRF2 and CREST (**f**) in G1-phase U-2 OS cells as in (**a**). Means ± SD of three independent experiments are shown (*n* = 100 cells per experiment). ns *p* = 0.1955 (**e**) and ** *p* = 0.0010 (**f**) (two-tailed unpaired *t* test). Scale bars, 10 µm. Source data are provided as a Source Data file.

To determine whether other fragile, difficult-to-replicate genomic regions are similarly prone to undergo post-MiDAS, we focused on centromeres, which frequently experience replication stress and were previously shown to rely on homologous recombination for the repair of centromeric DNA breaks in G1 to maintain centromeric integrity^47,48^. Indeed, co-staining for CREST as a centromeric marker revealed a co-localization of almost one-third of all post-MiDAS sites in untreated G1 cells with centromeres (Fig. 2a, b), and the number of centromeres that were positive for EdU incorporation in G1 increased upon enhanced replication stress (Fig. 2c). Thus, in addition to telomeres, centromeric regions also seem to undergo post-MiDAS in response to endogenous and exogenous replication stress.

Interestingly, through iterative indirect immunofluorescence imaging^49^, we found that around one quarter of post-MiDAS sites co-localized with both telomere and centromere foci in untreated U-2 OS cells (Fig. 2d, e), and that enhanced replication stress increased the number of EdU foci that were positive for both TRF2 and CREST (Fig. 2f). These results suggest that repetitive telomere and centromere sequences with replication stress-derived inherited ssDNA lesions may cluster together during G1 and share the same local repair environment during post-MiDAS.

### ALT factors function in the regulation of post-MiDAS

**Fig. 3:**
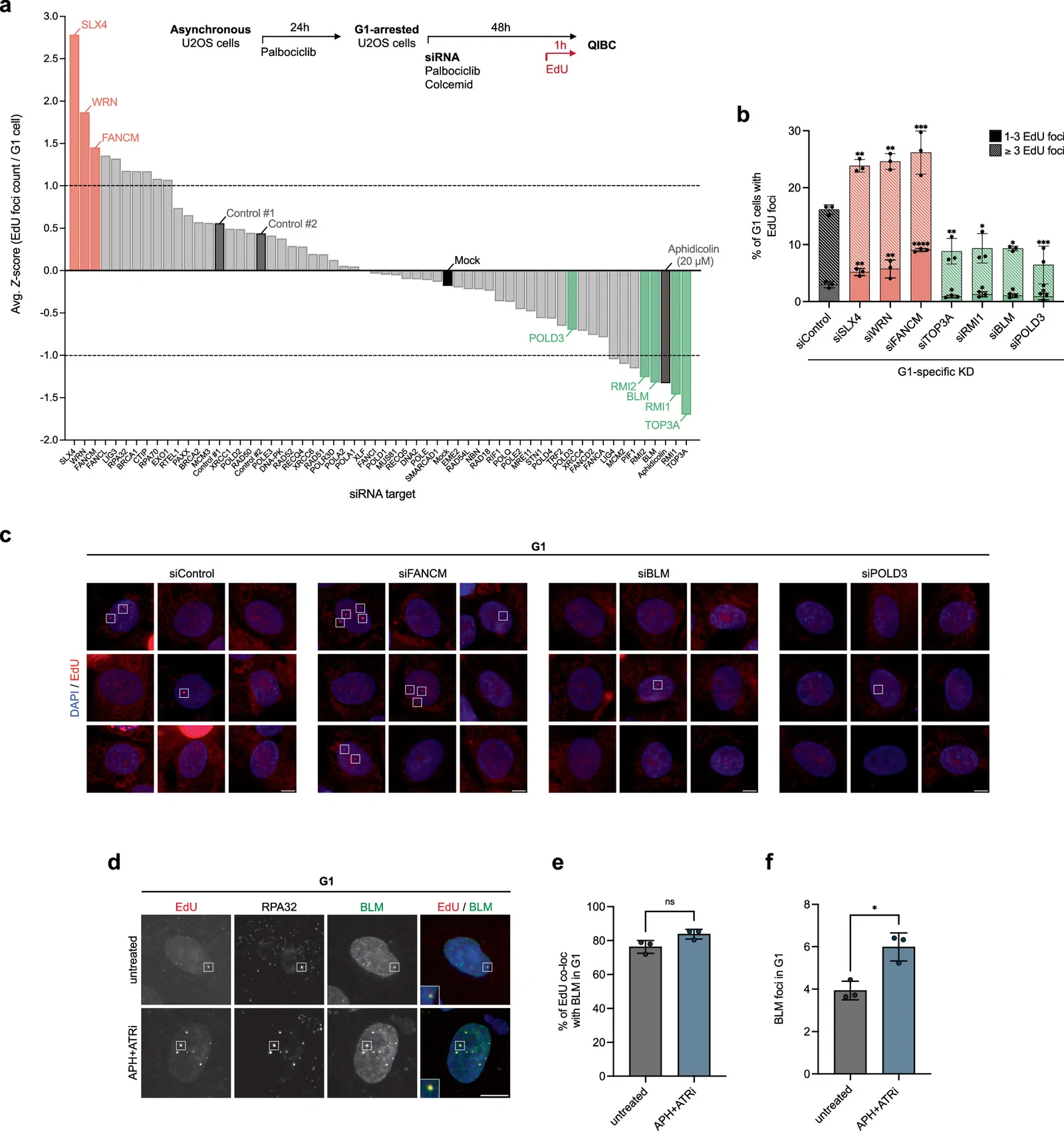
A G1-targeted siRNA screen reveals the involvement of ALT factors in post-MiDAS. **a** Schematic of the QIBC-based targeted siRNA screen in G1-synchronized U-2 OS cells. Ranked average *Z*-scores of the average EdU foci count per G1-phase cell following siRNA-mediated knockdown of 56 target genes, transfection with non-targeting negative control siRNAs (Control #1 and #2), mock transfection without siRNA, or treatment with APH (20 µM) during the EdU pulse to suppress DNA synthesis. *n* > 400 G1-phase cells per condition. Average Z-scores from two independent experiments are shown. **b** Percentage of G1-phase U-2 OS cells with 1–3 or ≥ 3 EdU foci following G1-specific transfection with control, SLX4, WRN, FANCM, TOP3A, RMI1, BLM, or POLD3 siRNA. Means ± SD of three replicates are shown (*n* = 900 cells per replicate). 1–3 EdU foci: ** (siSLX4) *p* = 0.0083, ** (siWRN) *p* = 0.0012, **** (siFANCM) *p* < 0.0001, * (siTOP3A) *p* = 0.0123, * (siRMI1) *p* = 0.0447, * (siBLM) *p* = 0.0207, and * (siPOLD3) *p* = 0.0123 (one-way ANOVA with Dunnett’s multiple-comparison test). ≥ 3 EdU foci: ** (siSLX4) *p* = 0.0043, ** (siWRN) *p* = 0.0018, *** (siFANCM) *p* = 0.0003, ** (siTOP3A) *p* = 0.0062, * (siRMI1) *p* = 0.0110, * (siBLM) *p* = 0.0106, and *** (siPOLD3) *p* = 0.0005 (one-way ANOVA with Dunnett’s multiple-comparison test). **c** Representative images of EdU foci in G1-phase U-2 OS cells following G1-specific transfection with control, FANCM, BLM, or POLD3 siRNA. **d** Representative images of EdU, RPA32, and BLM foci co-localization in G1-phase U-2 OS cells, untreated or treated with APH (0.2 µM) and ATRi (1 µM) for 24 h. **e**,** f** Percentage of EdU foci co-localizing with BLM (**e**) and average BLM foci count per nucleus (**f**) in G1-phase U-2 OS cells as in (**d**). Means ± SD of three independent experiments are shown (*n*_1_ = 100, *n*_2_ = 100, and *n*_3_ = 90 (**e**); *n*_1_ = 126, *n*_2_ = 209, and *n*_3_ = 230 (**f**); *n* values indicate cells per experiment). ns *p* = 0.0538 (**e**) and * *p* = 0.0109 (**f**) (two-tailed unpaired *t* test). Scale bars, 10 µm. Source data are provided as a Source Data file.

To identify and characterize the factors involved in post-MiDAS, we performed a high-content microscopy-based small interfering RNA (siRNA) screen targeting genes with known roles in the DNA damage response (DDR), the replication stress response (RSR), ALT, and MiDAS (Fig. 3a and Supplementary Data 1). To screen for potential post-MiDAS regulators without interference from earlier cell cycle phases, we synchronized asynchronously growing U-2 OS cells in G1 by a 24 h treatment with the CDK4/6 inhibitor Palbociclib, which arrested 70-80% of the cell population in G1 and induced markedly lower levels of DNA damage compared to other G1-synchronization methods (Supplementary Fig. 4a, b). We then transfected the G1-synchronized, but otherwise untreated cell population for 48 h with siRNAs in the presence of Palbociclib and the mitotic inhibitor colcemid. Colcemid was added to prevent cells that escaped the G1 arrest, from going through mitosis and re-entering G1 during the knockdown period. Live cell imaging of U-2 OS H2B-GFP cells under these conditions confirmed that G1 re-entry was effectively blocked (Supplementary Fig. 4c, d and Supplementary Movies S1–3). Depletion of TRF2, used here as a control, was efficient in G1-specific knockdown conditions (Supplementary Fig. 4e). The G1-arrested cells re-entered S-phase upon release from Palbociclib, indicating that the arrest was reversible (Supplementary Fig. 4f, g). During the G1 arrest, global BrdU (5-bromo-2′-deoxyuridine) incorporation was abolished in the G1 cell population, demonstrating that S-phase entry was effectively blocked during the Palbociclib treatment (Supplementary Fig. 4h–j). QIBC enabled G1-focused readouts in cells with a 2 N DNA content as before (Fig. 1a, b and Supplementary Fig. 4a). To identify post-MiDAS regulators, we scored EdU foci counts per G1 nucleus of on average 400 G1 cells per condition (Fig. 3a and Supplementary Fig. 4a). EdU staining specificity was confirmed by a control condition in which a high dose of the DNA polymerase inhibitor APH was applied during the EdU pulse (Fig. 3a).

Remarkably, the G1-specific siRNA screen identified all core components of the BTR/BTRR complex (BLM, TOP3A, RMI1, and RMI2) as the strongest hits, showing the lowest *Z*-score for average EdU foci per G1 cell upon their G1-specific depletion, suggesting a role of these factors in promoting post-MiDAS in G1 (Fig. 3a). Similarly, the G1-specific depletion of the DNA Polymerase δ subunit POLD3 decreased EdU foci formation in G1 compared to control conditions, indicating a POLD3 requirement during post-MiDAS (Fig. 3a). Independent validation experiments confirmed that G1-specific depletion of the BTR complex and POLD3 not only decreased average EdU foci numbers per G1 nucleus but also reduced the fraction of post-MiDAS-positive G1 cells (Fig. 3b, c and Supplementary Fig. 5a, e–h). These findings extend recent reports on the functional roles of the Bloom Syndrome helicase BLM and the BTR complex in promoting telomere DNA synthesis by ALT in G2-phase, including telomere sequestration into APBs, telomeric G-quadruplex resolution, assembly of the ALT replisome, branch migration during telomere DNA synthesis, and the processing of HR intermediates^50–55^. Our results suggest that the BTR complex and POLD3 execute similar functions at telomeres that escaped faithful and complete telomere extension in G2 and mitosis and undergo post-MiDAS in G1. Consistent with these findings and supporting a direct role of BLM at post-MiDAS sites in G1, we observed recruitment of BLM to over 75% of post-MiDAS sites under both unperturbed and replication stress conditions (Fig. 3d–f).

In addition to the BTR complex and POLD3 as positive regulators of post-MiDAS, we identified the structure-specific endonuclease subunit SLX4, the RecQ-like DNA helicase and exonuclease WRN, and the ATP-dependent translocase Fanconi anemia group protein M (FANCM) as post-MiDAS suppressors, as their G1-specific depletion resulted in significantly increased post-MiDAS activity (Fig. 3a). Consistently, depletion of SLX4, WRN, or FANCM in G1 increased EdU foci counts in G1 and the percentage of post-MiDAS-positive G1 cells in independent validation experiments, thus confirming their role as negative regulators of post-MiDAS (Fig. 3b, c and Supplementary Fig. 5a–d). In agreement with these findings, SLX4, as part of the SMX endonuclease complex, was reported to antagonize ALT telomere replication mediated by the BTR complex through promoting premature resolution of HR-intermediates^54^. Moreover, we previously showed that depletion of FANCM in asynchronously growing cells enhances post-MiDAS^40^. In principle, this could be explained by functions of FANCM during S/G2, where it suppresses replication stress at telomeres through replication fork remodeling and reduces R-loop levels and BLM activity^56–58^. Elevated post-MiDAS upon FANCM depletion was dependent on BLM in our conditions, because BLM co-depletion abolished EdU foci induction in G1 (Supplementary Fig. 5i–l). The antagonistic effects observed after G1-specific depletion of FANCM and BLM, together with the recruitment of BLM to post-MiDAS sites, suggest direct roles for FANCM and BLM during post-MiDAS in G1. This notion is further supported by acute, short-term treatment with a BLM inhibitor (BLM-IN-1), which resulted in reduced EdU foci detection at TRF1-FokI-induced telomere damage in G1 (Supplementary Fig. 5m).

Together, our results indicate that post-MiDAS and ALT in S/G2 and mitosis share a set of common cellular factors, including antagonistic roles of the BTR complex and FANCM-SLX4, despite occurring in different cell cycle phases and despite differences in the availability of sister chromatid sequences as repair templates.

### Post-MiDAS telomeres are sequestered into APBs promoted by SUMOylation

**Fig. 4:**
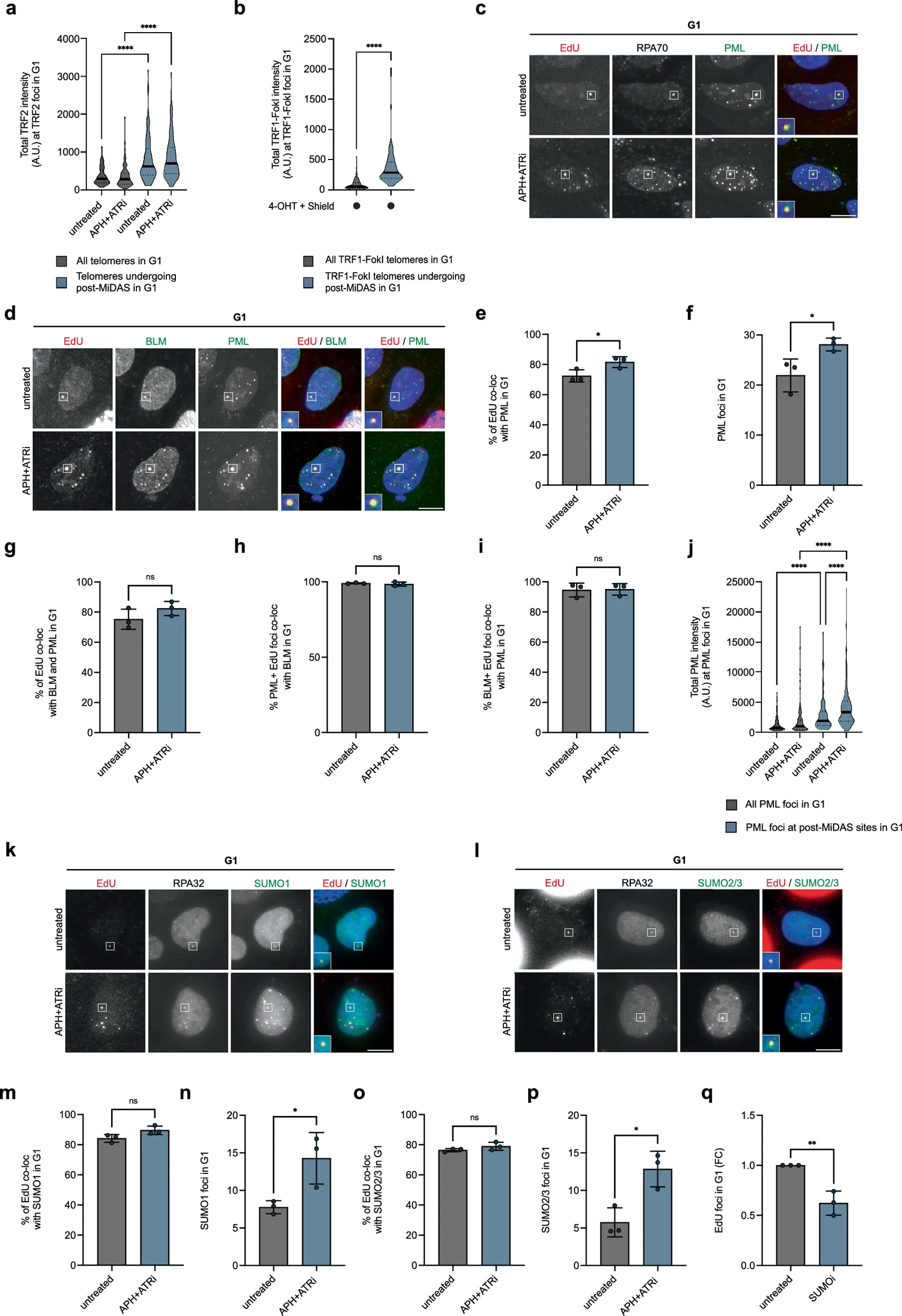
Post-MiDAS is associated with SUMOylation, PML, and telomere clustering in APBs. **a** Violin plot of total TRF2 intensity (A.U.) at EdU-positive and all detected TRF2 foci in G1-phase U-2 OS cells. Cells were untreated or treated with APH (0.2 µM) and ATRi (1 µM) for 24 h (*n* = 100 foci per condition). Solid lines represent the median; dashed lines indicate the upper and lower quartiles. **** *p* < 0.0001 (one-way ANOVA with Tukey’s multiple-comparison test). **b** Violin plot of total TRF1-FokI intensity (A.U.) at EdU-positive and all detected TRF1-FokI foci in G1-phase U-2 OS TRF1-FokI cells treated with 4-OHT (1 µM) and Shield-1 (1 µM) for 2 h (*n* = 100 foci per condition). Solid lines represent the median; dashed lines indicate the upper and lower quartiles. **** *p* < 0.0001 (two-tailed unpaired *t* test). **c**, **d** Representative images of EdU, RPA70, and PML (**c**) or EdU, BLM, and PML (**d**) foci co-localization in G1-phase U-2 OS cells as in (**a**). **e**–**i** Percentage of EdU foci co-localizing with PML (**e**), average PML foci count per nucleus (**f**), percentage of EdU foci co-localizing with BLM and PML (**g**), percentage of PML-positive EdU foci co-localizing with BLM (**h**), and percentage of BLM-positive EdU foci co-localizing with PML (**i**) in G1-phase U-2 OS cells as in (**a**). Means ± SD of three independent experiments are shown (*n*_1_ = 100, *n*_2_ = 100, and *n*_3_ = 90 (**e**, **g**); *n*_1_ = 174, *n*_2_ = 182, and *n*_3_ = 621 (**f**); *n*_1_ = 87, *n*_2_ = 86, and *n*_3_ = 91 (**h**); and *n*_1_ = 90, *n*_2_ = 84, and *n*_3_ = 91 (**i**); *n*-values indicate cells per experiment). * *p* = 0.0434 (**e**), * *p* = 0.0386 (**f**), ns *p* = 0.2051 (**g**), ns *p* = 0.5449 (**h**), and ns *p* = 0.9134 (**i**) (two-tailed unpaired *t* test). **j** Violin plot of total PML foci intensity (A.U.) at EdU-positive and all detected PML foci in G1-phase U-2 OS cells treated as in (**a**) (*n* = 150 foci per condition). Solid lines represent the median; dashed lines indicate the upper and lower quartiles. **** *p* < 0.0001 (one-way ANOVA with Tukey’s multiple-comparison test). **k**,** l** Representative images of EdU, RPA32, and SUMO1 (**k**) or EdU, RPA32, and SUMO2/3 (**l**) foci co-localization in G1-phase U-2 OS cells as in (**a**). **m**–**p** Percentage of EdU foci co-localizing with SUMO1 (**m**), average SUMO1 foci count per nucleus (**n**), percentage of EdU foci co-localizing with SUMO2/3 (**o**), and average SUMO2/3 foci count per nucleus (**p**) in G1-phase U-2 OS cells as in (**a**). Means ± SD of three independent experiments are shown (*n*_1_ = 100, *n*_2_ = 100, and *n*_3_ = 85 (**m**); *n*_1_ = 98, *n*_2_ = 136, and *n*_3_ = 600 (**n**); *n*_1_ = 100, *n*_2_ = 100, and *n*_3_ = 60 (**o**); and *n*_1_ = 242, *n*_2_ = 153, and *n*_3_ = 182 (**p**); *n* values indicate cells per experiment). ns *p* = 0.0697 (**m**), * *p* = 0.0331 (**n**), ns *p* = 0.1941 (**o**), and * *p* = 0.0159 (**p**) (two-tailed unpaired *t* test). **q** Normalized EdU foci count (fold change, FC) per G1-phase U-2 OS nucleus untreated or treated with SUMOi (ML-792, 1 µM) for 2 h. Means ± SD of three independent experiments, normalized to the untreated control, are shown (*n* = 100 cells per experiment). ** *p* = 0.0056 (two-tailed unpaired *t* test). A.U., arbitrary units. Scale bars, 10 µm. Source data are provided as a Source Data file.

Considering the identified contributions of ALT factors to post-MiDAS and our previous findings that particularly ALT-positive cancer cells use post-MiDAS^40^, we investigated whether telomere clustering into APBs, a hallmark of ALT^59^, might also contribute to post-MiDAS. To this end, we compared the total TRF2 foci intensity at telomeres in G1 undergoing post-MiDAS with the total TRF2 foci intensity across all detected telomeres, marked by TRF2, per G1 nucleus. Post-MiDAS at telomeres was strongly associated with larger and brighter TRF2 foci and a two-fold increase in total TRF2 foci intensity compared to the average across all telomeres per G1 nucleus, an effect which was further enhanced upon replication stress (Fig. 4a and Supplementary Fig. 6a). Consistently, when analyzing telomeres with TRF1-FokI-induced DNA damage in G1 cells, we observed that particularly the larger and brighter TRF1-FokI foci were sites of post-MiDAS, with a significantly higher TRF1-FokI intensity compared to the average across all TRF1-FokI foci per G1 nucleus (Fig. 4b and Supplementary Fig. 6b). These results suggest that telomeres undergoing post-MiDAS in the absence of sister chromatids tend to cluster together in G1.

Moreover, the majority of post-MiDAS sites co-localized with bright PML and BLM foci under both unperturbed and replication stress conditions, indicating APB formation at clustered telomeres for post-MiDAS in G1 (Fig. 4c–i). In line, predominantly large and bright PML bodies with a significantly higher PML foci intensity than the average per G1 nucleus were prominent sites of post-MiDAS activity (Fig. 4c, d, j and Supplementary Fig. 6c, d). Collectively, these findings suggest that ALT telomeres, which experienced replication stress or DNA damage, are sequestered in APBs, where they are enriched and cluster while undergoing post-MiDAS in G1.

The formation of APBs is driven by SUMOylation of telomere proteins such as TRF1 and TRF2, as well as PML itself^60,61^. Consistently, we found that 75–90% of post-MiDAS sites were associated with strong enrichment of the SUMO paralogues SUMO1 and SUMO2/3 in a replication stress-dependent manner (Fig. 4k–p). Inhibition of SUMOylation using the SUMO inhibitor ML792 (SUMOi) led to a clear reduction in the average EdU foci count per G1 nucleus (Fig. 4q and Supplementary Fig. 6e–h), suggesting that SUMOylation, similar to its role in the ALT pathway in S/G2, promotes APB formation and contributes to efficient post-MiDAS in G1.

### Factors associated with MiDAS and DNA damage tolerance contribute to post-MiDAS

**Fig. 5:**
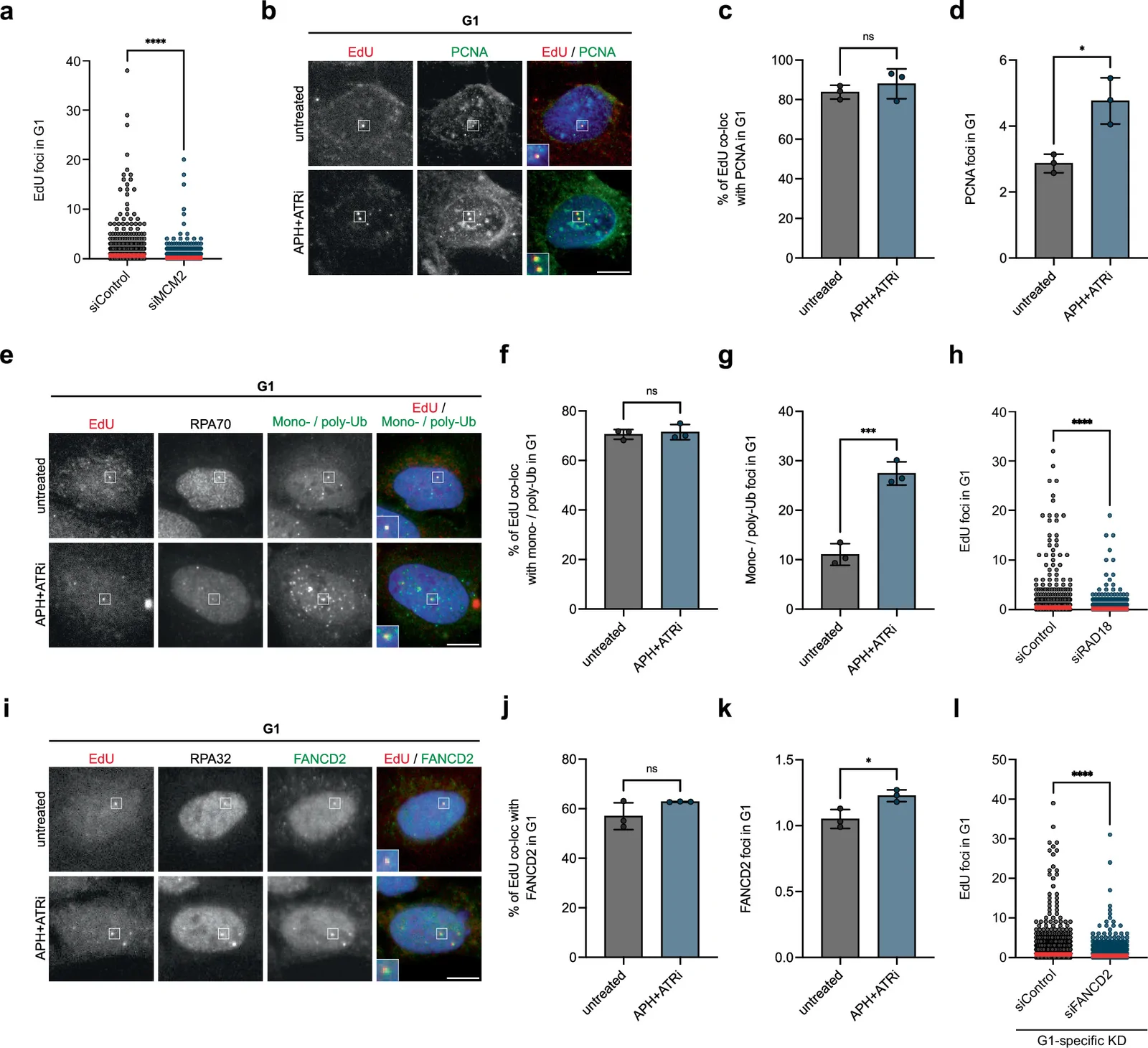
Factors associated with MiDAS and DNA damage tolerance contribute to post-MiDAS. **a** EdU foci count per G1-phase U-2 OS nucleus following transfection with control or MCM2 siRNA. Scatterplots show *n* = 2000 cells per condition. Red lines represent the mean. **** *p* < 0.0001 (two-tailed unpaired *t* test). **b** Representative images of EdU and PCNA foci co-localization in G1-phase U-2 OS cells, untreated or treated with APH (0.2 µM) and ATRi (1 µM) for 24 h. **c**, **d** Percentage of EdU foci co-localizing with PCNA (**c**) and average PCNA foci count per nucleus (**d**) in G1-phase U-2 OS cells as in (**b**). Means ± SD of three independent experiments are shown (*n* = 100 (**c**); and *n*_1_ = 161, *n*_2_ = 147, and *n*_3_ = 110 (**d**); *n-*values indicate cells per experiment). ns *p* = 0.4319 (**c**) and * *p* = 0.0121 (**d**) (two-tailed unpaired *t* test). **e** Representative images of EdU, RPA70, and mono- and polyubiquitin conjugate co-localization in G1-phase U-2 OS cells as in (**b**). **f**,** g** Percentage of EdU foci co-localizing with mono- and polyubiquitin conjugates (**f**) and average mono- and polyubiquitin conjugate foci count per nucleus (**g**) in G1-phase U-2 OS cells as in (**b**). Means ± SD of three independent experiments are shown (*n*_1_ = 100, *n*_2_ = 100, and *n*_3_ = 90 (**f**); and *n*_1_ = 380, *n*_2_ = 742, and *n*_3_ = 808 (**g**); *n-*values indicate cells per experiment). ns *p* = 0.6839 (**f**) and *** *p* = 0.0009 (**g**) (two-tailed unpaired *t* test). **h** EdU foci count per G1-phase U-2 OS nucleus following transfection with control or RAD18 siRNA. Scatterplots show *n* = 3000 cells per condition. Red lines represent the mean. **** *p* < 0.0001 (two-tailed unpaired *t* test). **i** Representative images of EdU, RPA32, and FANCD2 foci co-localization in G1-phase U-2 OS as in (**b**). **j**,** k** Percentage of EdU foci co-localizing with FANCD2 (**j**) and average FANCD2 foci count per nucleus (**k**) in G1-phase U-2 OS cells as in (**b**). Means ± SD of three independent experiments are shown (*n* = 100 (**j**); and *n*_1_ = 1567, *n*_2_ = 7000, and *n*_3_ = 7000 (**k**); *n-*values indicate cells per experiment). ns *p* = 0.1359 (**j**), and * *p* = 0.0228 (**k**) (two-tailed unpaired *t* test). **l** EdU foci count per G1-phase U-2 OS nucleus following G1-specific transfection with control or FANCD2 siRNA. Scatterplots show *n* = 2300 cells per condition. Red lines represent the mean. **** *p* < 0.0001 (two-tailed unpaired *t* test). Scale bars, 10 µm. Source data are provided as a Source Data file.

The DNA helicase subunit MCM2, a core component of the MCM2-7 replicative helicase complex, and the sliding clamp proliferating cell nuclear antigen (PCNA), which ensures polymerase processivity, are both required for canonical DNA replication initiation and elongation^62,63^. Notably, depletion of MCM2 in asynchronous cells, as well as its G1-specific knockdown in our siRNA screen, resulted in reduced post-MiDAS, indicating that MCM helicase activity is needed not only for canonical S-phase replication but also for post-MiDAS in G1 (Figs. 3a, 5a and Supplementary Fig. 7a–c). Moreover, the majority of post-MiDAS events co-localized with PCNA under both unperturbed and replication stress conditions, suggesting de novo DNA synthesis at post-MiDAS sites in G1 cells (Fig. 5b–d and Supplementary Fig. 7d).

Besides its central role in canonical S-phase DNA replication, PCNA is also important for DNA damage tolerance pathways and MiDAS in response to replication stress. In MiDAS, RAD18-mediated monoubiquitylation of PCNA promotes DNA lesion bypass and polymerase switching^64^. PCNA ubiquitylation promotes the recruitment of Fanconi anemia (FA) group D2 protein (FANCD2) and, subsequently, of REV1 and REV3, as well as POLD3, to FANCD2-marked under-replicated DNA to initiate MiDAS via translesion synthesis (TLS)^65^. MiDAS initiation is then followed by a POLD3-dependent polymerase switch that enables processive DNA synthesis by DNA polymerase δ to complete replication^64,65^. Considering the potential mechanistic parallels between MiDAS and post-MiDAS in G1 and our findings that POLD3, MCM2, and PCNA are all required for post-MiDAS, we tested the contribution of RAD18-dependent events to post-MiDAS. Indeed, we detected ubiquitylation at around 70% of post-MiDAS sites using an antibody against mono- and polyubiquitylated conjugates (Fig. 5e–g and Supplementary Fig. 7e, f). Furthermore, depletion of RAD18 resulted in impaired post-MiDAS (Fig. 5h and Supplementary Fig. 7g, h). Consistent with its reported role in marking under-replicated genomic regions during MiDAS^65^, FANCD2 was present at approximately 60% of post-MiDAS sites in G1 (Fig. 5i–k). Moreover, the depletion of FANCD2 in G1 significantly reduced post-MiDAS in our siRNA screen and in independent validation experiments, demonstrating that FANCD2 contributes to post-MiDAS (Figs. 3a, 5l and Supplementary Fig. 7i–k). Taken together, these results suggest that post-MiDAS employs canonical DNA replication factors, including the MCM helicase and PCNA, but also RAD18 and FANCD2, for DNA synthesis at ALT telomeres in G1.

### Proteome-wide identification of post-MiDAS factors by targeted quantitative proteomics

**Fig. 6:**
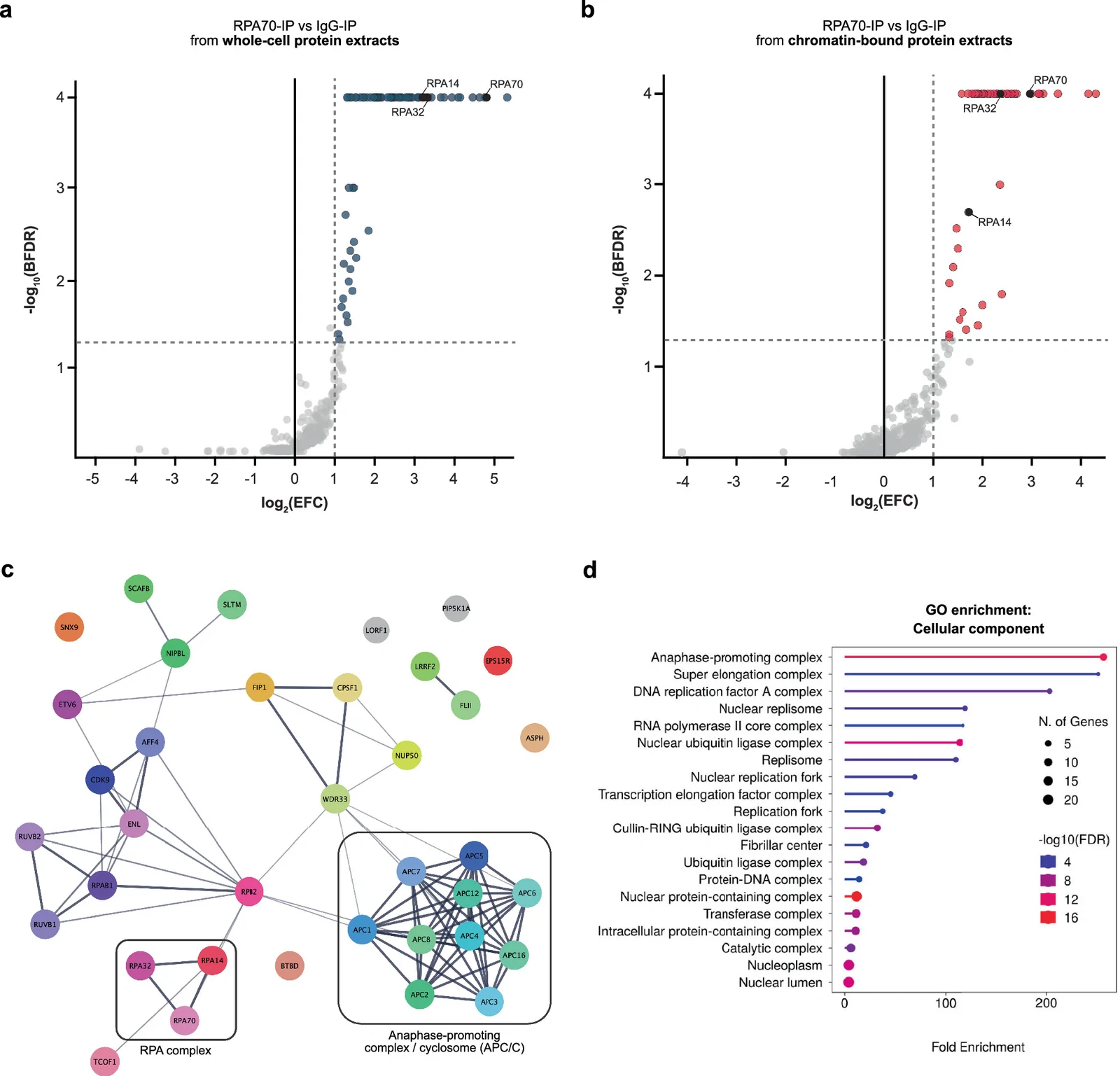
Targeted proteomics to identify putative post-MiDAS factors in G1. **a** Scatterplot of RPA70 interactors in G1 identified by RPA70-IP MS from whole-cell protein extracts of FACS-sorted G1-phase U-2 OS FUCCI cells. Data from quadruplicate samples per condition were analyzed by SAINT. Significantly enriched proteins (EFC ≥ 2; BFDR ≤ 0.05) are highlighted in blue. Components of the trimeric RPA complex are highlighted in black. **b** Scatterplot of RPA70 interactors in G1 identified by RPA70-IP MS from chromatin-bound protein extracts of FACS-sorted G1-phase U-2 OS FUCCI cells. Data from quadruplicate samples per condition were analyzed by SAINT. Significantly enriched proteins (EFC ≥ 2; BFDR ≤ 0.05) are highlighted in pink. Components of the trimeric RPA complex are highlighted in black. **c** STRING protein interaction network of the 37 overlapping significantly enriched proteins identified as RPA interactors in G1 by RPA70-IP MS from both whole-cell and chromatin-bound protein extracts of FACS-sorted G1-phase U-2 OS FUCCI cells. **d** Gene ontology (GO) analysis for “Cellular component” of the 37 overlapping significantly enriched proteins identified as RPA interactors in G1 by RPA70-IP MS from both whole-cell and chromatin-bound protein extracts of FACS-sorted G1-phase U-2 OS FUCCI cells. Source data are provided as a Source Data file.

To screen for post-MiDAS factors proteome-wide, we analyzed the G1-specific interactome of the post-MiDAS marker RPA^40^. To this end, we first enriched U-2 OS cells stably expressing FUCCI reporters^45^ in G1 using Palbociclib, and then FACS-sorted G1 cells based on their RFP-CDT1 fragment-positive and GFP-Geminin fragment-negative fluorescence signals and additionally based on their 2 N DNA content measured by Hoechst 33342. This approach yielded a highly pure population of > 99% G1-phase cells (Supplementary Fig. 8a–c). From this population, we immunoprecipitated endogenous RPA70 either from whole-cell lysates or, in an independent approach, from chromatin-bound protein fractions (Supplementary Fig. 8d) and analyzed the RPA interactome by label-free quantitative mass spectrometry. Compared to the IgG negative controls, this revealed a total of 76 and 47 specific RPA70 interactors in G1 from whole-cell and chromatin-bound extracts, respectively, with an overlap of 37 common interactors (empirical fold change (EFC) ≥ 2; Bayesian false discovery rate (BFDR) ≤ 0.05) (Fig. 6a–c, Supplementary Fig. 8e and Supplementary Data 2 and 3). The shared RPA70 interactors included all components of the heterotrimeric RPA complex. In addition, several components of the RNA Polymerase II transcription machinery, multiple proteins involved in pre-mRNA cleavage and processing, and 10 of the 14 subunits of the E3 ubiquitin ligase complex anaphase promoting complex/cyclosome (APC/C) were identified (APC1, APC2, APC3, APC4, APC5, APC6, APC7, APC8, APC12, and APC16) (Fig. 6c and Supplementary Fig. 8f, g). Consistently, gene ontology (GO) analysis of the 37 common RPA70 interactors identified from whole-cell and chromatin-bound protein extracts revealed a strong enrichment of the APC/C (Fig. 6d). We further validated these results orthogonally and extended them by quantitative proximity labeling proteomics based on TurboID-mediated biotinylation of proteins in the vicinity of the RPA complex in G1 cells. Similar to the RPA70 interactome analysis, we FACS-sorted highly pure G1 populations from U-2 OS FUCCI cells, which we engineered to additionally express TurboID-RPA32 (Supplementary Fig. 9a–e). This approach identified not only all three RPA subunits, but also 8 of the 14 components of the APC/C (Supplementary Fig. 9f). In addition, the sensitive TurboID approach revealed BLM, TOP3A, RMI1, RMI2, FANCM, SLX4, WRN, PML, PCNA, and RAD18 in proximity to RPA32 in G1 cells, consistent with their localization and function at RPA-marked post-MiDAS sites (Supplementary Fig. 9f and Supplementary Data 4). Comparing the sets of significantly enriched RPA interactors from the three complementary proteomics approaches revealed 29 shared and thus high-confidence interactors, including 7 subunits of the APC/C (Supplementary Fig. 9g, h).

### APC/C^CDH1^ keeps excessive post-MiDAS in G1 in check

**Fig. 7:**
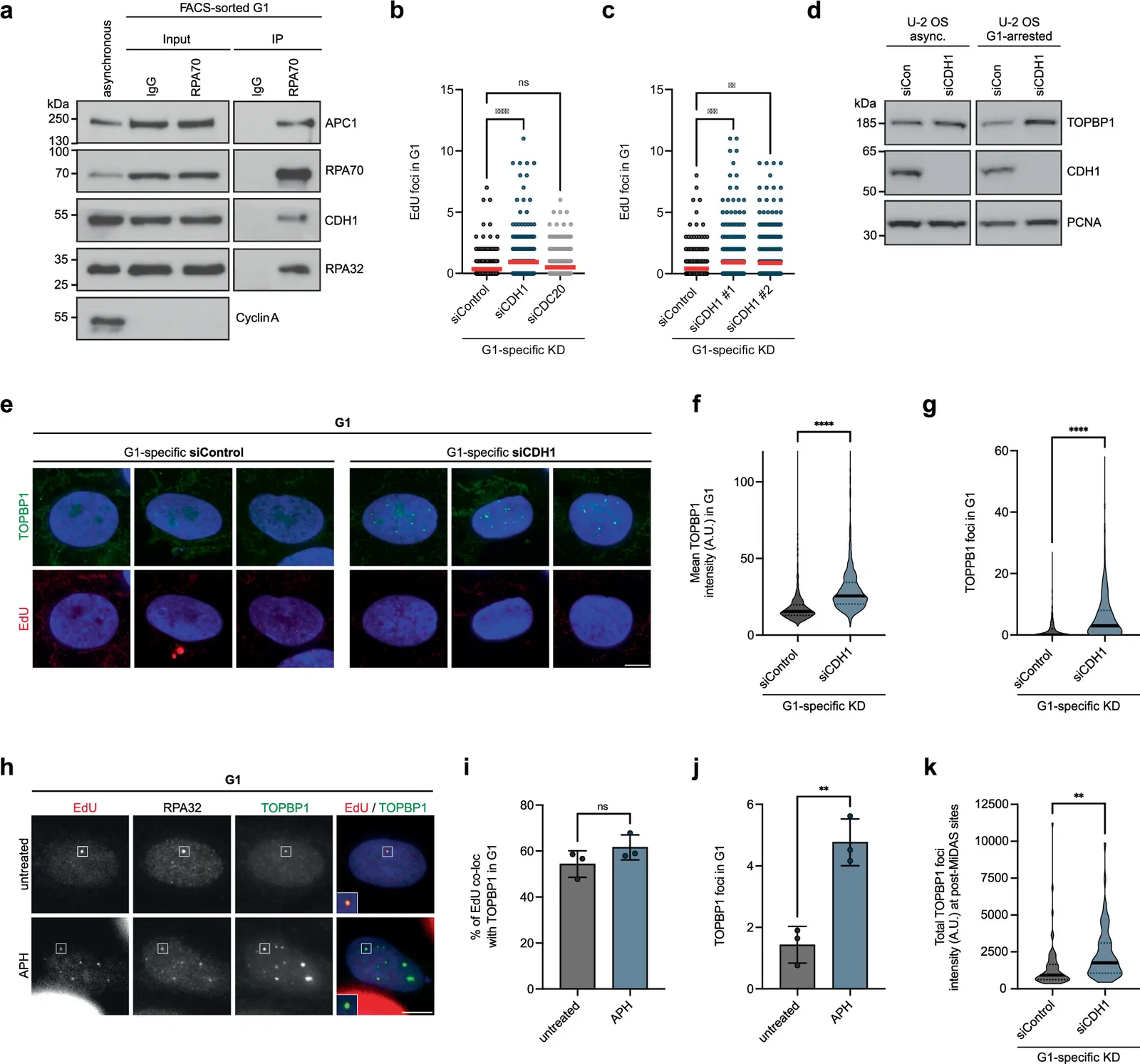
APC/C^CDH1^ restrains excessive post-MiDAS in G1. **a** Western blot analysis after immunoprecipitation of endogenous RPA70 from FACS-sorted G1-phase U-2 OS FUCCI cells (G1 sorting confirmed by Cyclin A). Control IgG and RPA70 immunoprecipitates were probed for endogenous RPA70, APC1, CDH1, and RPA32. **b**,** c** EdU foci count per G1-phase U-2 OS nucleus following G1-specific transfection with control, CDH1, or CDC20 siRNA (**b**), or control or two independent CDH1 (CDH1 #1 and CDH1 #2) siRNAs (**c**). Scatterplots show *n* = 300 cells per condition. Red lines represent the mean. **** *p* < 0.0001 and ns *p* = 0.3471 (**b**), and *** *p* = 0.0005 and ** *p* = 0.0017 (**c**) (one-way ANOVA with Tukey’s multiple-comparison test). **d** Western blot analysis of TOPBP1, CDH1, and PCNA protein levels in whole-cell lysates of asynchronous (async.) and G1-arrested U-2 OS cells following transfection with control or CDH1 siRNA. **e** Representative images of EdU and TOPBP1 foci co-localization in G1-phase U-2 OS cells following G1-specific transfection with control or CDH1 siRNA. **f**,** g** Violin plot of mean TOPBP1 intensity (A.U.) (**f**) and TOPBP1 foci count (**g**) per G1-phase U-2 OS nucleus following G1-specific transfection with control or CDH1 siRNA (*n* = 520 cells per condition). Solid lines represent the median; dashed lines indicate the upper and lower quartiles. **** *p* < 0.0001 (two-tailed unpaired *t* test). **h** Representative images of EdU, RPA32, and TOPBP1 foci co-localization in G1-phase U-2 OS cells, untreated or treated with APH (0.2 µM) for 24 h. **i**,** j** Percentage of EdU foci co-localizing with TOPBP1 (**i**) and average TOPBP1 foci count per nucleus (**j**) in G1-phase U-2 OS cells as in (**h**). Means ± SD of three independent experiments are shown (*n* = 100 (**i**); and *n*_1_ = 301, *n*_2_ = 204, and *n*_3_ = 161 (**j**); *n-*values indicate cells per experiment). ns *p* = 0.1885 (**i**), and ** *p* = 0.0040 (**j**) (two-tailed unpaired *t* test). **k** Violin plot of total TOPBP1 foci intensity (A.U.) at post-MiDAS sites per G1-phase U-2 OS nucleus following G1-specific transfection with control or CDH1 siRNA (*n* = 70 cells per condition). Solid lines represent the median; dashed lines indicate the upper and lower quartiles. ** *p* = 0.0014 (two-tailed unpaired *t* test). A.U., arbitrary units. Scale bars, 10 µm. Source data are provided as a Source Data file.

To investigate a potential function of the APC/C in post-MiDAS and to validate its interaction with RPA, we immunoprecipitated endogenous RPA70 from FACS-sorted G1 cells and could readily detect the scaffolding APC/C subunit APC1, along with RPA32, as RPA70 interactors, thereby confirming the mass spectrometry data (Fig. 7a). The APC/C uses two mutually exclusive adapter proteins and co-activators, cell division cycle protein 20 (CDC20) and CDC20 homolog 1 (CDH1). Whereas CDC20 predominantly associates with the APC/C from early mitosis until anaphase, CDH1 is mainly active in telophase and G1^66,67^. Accordingly, CDH1 was associated with RPA in RPA70-immunoprecipitations from FACS-sorted G1 phase cells (Fig. 7a). Moreover, CDH1, but not CDC20, was detected in the G1-specific TurboID-RPA32 proximity labeling proteomics analyses (Supplementary Fig. 9f). We therefore investigated the potential function of CDH1 and, as a control, CDC20 in post-MiDAS by G1-specific siRNA-mediated knockdowns. Interestingly, G1-specific CDH1 depletion increased post-MiDAS by more than 2.5-fold compared to the control, whereas CDC20 depletion had a very minor effect, despite comparable knockdown efficiencies (Fig. 7b and Supplementary Fig. 10a–d). These results were confirmed with a second, independent siRNA against CDH1 (Fig. 7c and Supplementary Fig. 10e, f). Moreover, a proteomics-inspired targeted siRNA screen, which included multiple APC/C components that were identified as RPA interactors in G1, showed increased post-MiDAS activity upon siRNA-mediated depletion of most APC/C subunits, including APC1-6, 8, 10, and 12 (Supplementary Fig. 10g and Supplementary Data 5). These data suggest that APC/C^CDH1^, rather than APC/C^CDC20^, associates with RPA in G1 and negatively regulates post-MiDAS.

The replication checkpoint kinase ATR is recruited to telomeres by RPA and was previously shown to be involved in ALT-associated telomere recombination^68^. ATR activation at RPA-bound DNA lesions depends on DNA topoisomerase 2-binding protein 1 (TOPBP1)^69,70^, which localizes to under-replicated DNA in mitosis^71,72^ and forms nuclear condensates around inherited DNA lesions in G1^71,73^. Furthermore, TOPBP1 condensation has been implicated in ATR activation^74^. Interestingly, in silico prediction identified TOPBP1 as a putative APC/C substrate^75^. We found that G1-specific depletion of CDH1 led to elevated TOPBP1 levels and increased nuclear TOPBP1 condensates in G1 cells (Fig. 7d–g and Supplementary Fig. 10h, i, n, o), without increasing TOPBP1 mRNA expression (Supplementary Fig. 10k, l). Moreover, we detected an interaction between endogenous TOPBP1 and the APC/C components APC3 (CDC27) and CDH1 (Supplementary Fig. 10m). TOPBP1 was recruited to around 60% of post-MiDAS sites in a replication stress-dependent manner (Fig. 7h–j), and the G1-specific depletion of CDH1 increased the total TOPBP1 foci intensity at post-MiDAS sites compared to control conditions (Fig. 7k and Supplementary Fig. 10j, p). The CDH1 depletion-induced increase in TOPBP1 levels, nuclear TOPBP1 condensates in G1, and post-MiDAS activity were also observed in ALT-positive SaOS-2, GM-847, and SW26 cells (Supplementary Fig. 11a–i). In summary, these data implicate TOPBP1 as a potential APC/C substrate involved in post-MiDAS.

**Fig. 8:**
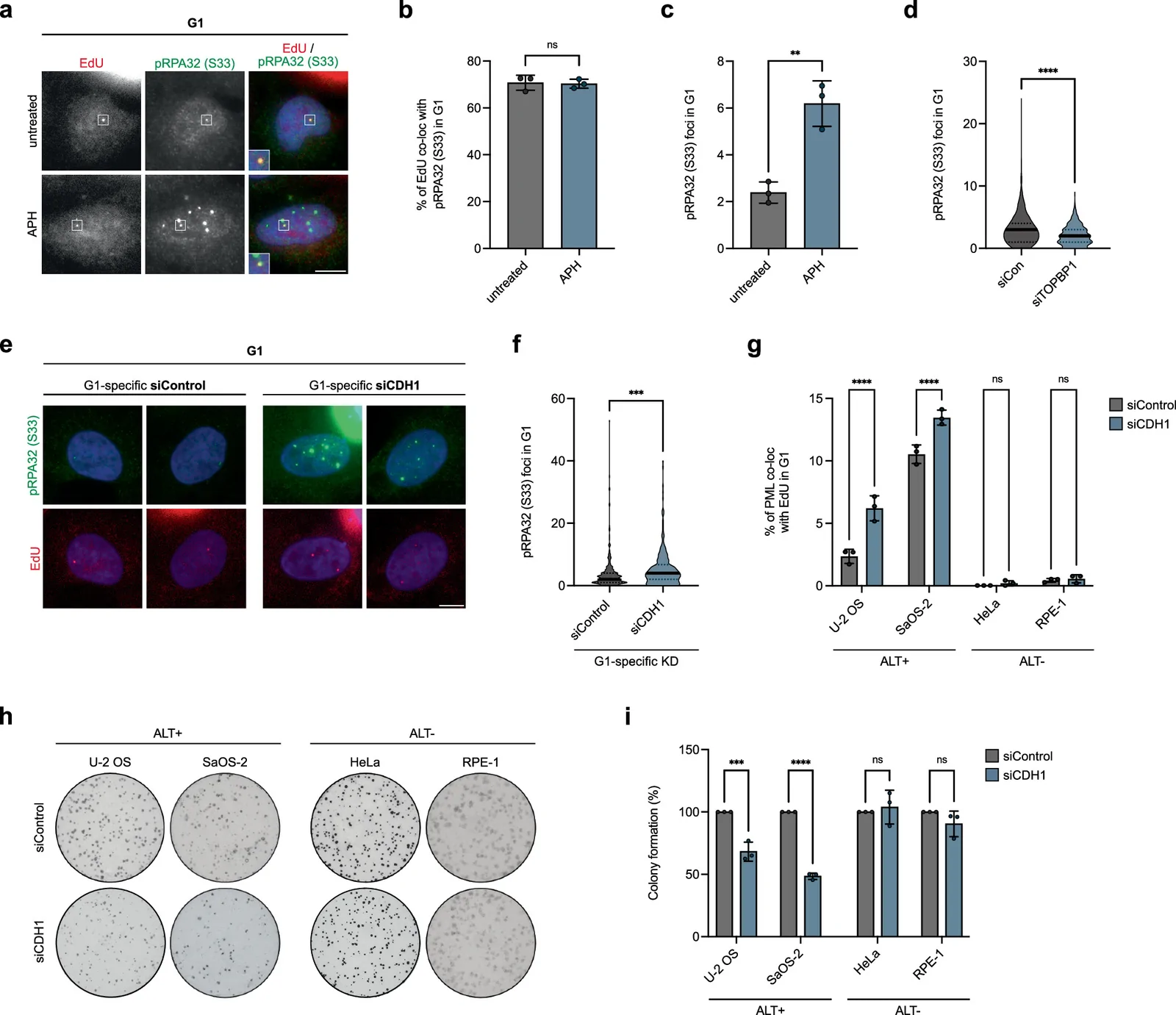
APC/C^CDH1^ activity is required in ALT-positive cancer cell lines. **a** Representative images of EdU and pRPA32 (S33) foci co-localization in G1-phase U-2 OS cells untreated or treated with APH (0.2 µM) for 24 h. **b**,** c** Percentage of EdU foci co-localizing with pRPA32 (S33) (**b**) and average pRPA32 (S33) foci count per nucleus (**c**) in G1-phase U-2 OS cells as in (**a**). Means ± SD of three independent experiments are shown (*n* = 100 (**b**); and *n*_1_ = 717, *n*_2_ = 286, and *n*_3_ = 481 (**c**); *n-*values indicate cells per experiment). ns *p* = 0.8661 (**b**) and ** *p* = 0.0036 (**c**) (two-tailed unpaired *t* test). **d** Violin plot of pRPA32 (S33) foci counts per G1-phase U-2 OS nucleus following transfection with control or TOPBP1 siRNA (*n* = 800 cells per condition). Solid lines represent the median; dashed lines indicate the upper and lower quartiles. **** *p* < 0.0001 (two-tailed unpaired *t* test). **e** Representative images of EdU and pRPA32 (S33) foci co-localization in G1-phase U-2 OS cells following G1-specific transfection with control or CDH1 siRNA. **f** Violin plot of pRPA32 (S33) foci count per G1-phase U-2 OS nucleus following G1-specific transfection with control or CDH1 siRNA (*n* = 300 cells per condition). Solid lines represent the median; dashed lines indicate the upper and lower quartiles. *** *p* = 0.0008 (two-tailed unpaired *t* test). **g** Percentage of PML foci co-localizing with EdU in G1-phase U-2 OS, SaOS-2, HeLa, and RPE-1 cells following transfection with control or CDH1 siRNA (*n* = 50 cells per experiment). Means ± SD of three independent experiments are shown. **** (U-2 OS and SaOS-2) *p* < 0.0001, ns (HeLa) *p* = 0.9866, and ns (RPE-1) *p* = 0.9973 (two-way ANOVA with Šídák’s multiple-comparison test). **h**,** i** Representative colony formation assays (**h**) and their quantification (**i**) with the indicated cell lines transfected with control or CDH1 siRNA. Colony formation was normalized to the siControl condition. Means ± SD of three independent experiments are shown. *** (U-2 OS) *p* = 0.0001, **** (SaOS-2) *p* < 0.0001, ns (HeLa) *p* = 0.9311, and ns (RPE-1) *p* = 0.3400 (two-way ANOVA with Šídák’s multiple-comparison test). Scale bars, 10 µm. Source data are provided as a Source Data file.

Considering the established role of TOPBP1 as an ATR activator^69,70^, we investigated whether non-canonical ATR activation might occur locally at post-MiDAS sites in G1. Indeed, we detected RPA32 phosphorylation at serine 33, a marker of ATR activity, at around 70% of post-MiDAS sites (Fig. 8a–c). Depletion of TOPBP1 impaired pRPA32 (S33) foci formation in G1 (Fig. 8d and Supplementary Fig. 12a, b), and acute ATR inhibition suppressed pRPA32 (S33) foci formation in G1 and reduced the elevated post-MiDAS activity upon FANCM depletion (Supplementary Fig. 12c–e). G1-specific depletion of the APC/C adapter CDH1, on the other hand, not only elevated TOPBP1 and post-MiDAS levels in G1 but also increased RPA32 phosphorylation at S33 in G1 cells (Fig. 8e, f and Supplementary Fig. 12f). Besides its known targets in G1^76^, APC/C^CDH1^ thus seems to regulate TOPBP1 levels and ATR activity in the context of post-MiDAS in ALT-positive cancer cells. Consistent with an ALT-specific function of CDH1 in the context of post-MiDAS, the co-localization of PML with EdU foci in G1 was increased upon CDH1 depletion in the ALT-positive cancer cell lines U-2 OS and SaOS-2, but not in the ALT-negative cell lines HeLa and RPE-1 (Fig. 8g). Notably, U-2 OS and SaOS-2 cells were also more sensitive to CDH1 depletion in colony formation assays than ALT-negative HeLa and RPE-1 cells (Fig. 8h, i), despite comparable knockdown efficiencies (Supplementary Fig. 12g), suggesting that APC/C^CDH1^ activity is particularly important for cancer cells that employ ALT and post-MiDAS for telomere maintenance.

## Discussion

DNA replication beyond S-phase is increasingly recognized as a mechanism for maintaining genome stability under conditions of elevated replication stress, particularly in cancer cells, and may help to complete replication of difficult-to-replicate genomic loci such as telomeres^77^. ALT telomeres, which experience high levels of replication stress, rely on extended DNA repair synthesis in G2^31^. In mitosis, MiDAS might serve as a last resort mechanism for under-replicated genomic regions, including ALT replication intermediates, before chromosome segregation^78,79^. The discovery of post-MiDAS at heritable RPA-marked ssDNA lesions in G1-phase extends the concept of unscheduled DNA replication beyond S-phase to the next cell generation and indicates that post-MiDAS may safeguard telomere integrity when MiDAS is incomplete or fails^40^.

Whether post-MiDAS also occurs at genomic loci other than telomeres had remained elusive. Here, we provide evidence that centromeres also undergo post-MiDAS in response to endogenous and experimentally enhanced replication stress. Although post-MiDAS at telomeres and centromeres can explain the EdU foci in G1 cells observed under the tested conditions, low levels of post-MiDAS activity might exist at additional, yet to be identified genomic regions. Like telomeres, centromeres contain long, highly repetitive sequences that form secondary structures, are heterochromatic and late-replicating, and are therefore susceptible to replication stress and centromere fragility^14,80^. Surprisingly, we found that telomeres and centromeres frequently co-localize at post-MiDAS sites in G1, suggesting that telomeres and centromeres with replication stress-derived ssDNA lesions can cluster during post-MiDAS. Their clustering in APBs may facilitate the recruitment of a shared post-MiDAS machinery acting at both types of genomic regions, with a potential for recombination between telomeric and centromeric DNA. Indeed, parallel work identified insertions of centromere-associated repetitive sequences at telomeres in ALT-positive cancer cell lines and primary pediatric neuroblastomas^81^. Considering the role of APBs in enriching clustered telomeres and BIR factors to drive ALT^27,50,82^, it will be interesting to investigate in future studies whether APB formation facilitates the clustering of telomeres and centromeres at post-MiDAS sites in G1. In support of this notion, telomeres undergoing post-MiDAS, triggered either by replication stress or FokI-induced telomeric DSBs, showed increased TRF2 and PML foci intensities. In line with previous studies that identified SUMOylation as a driver of APB formation^83^, we found that the majority of post-MiDAS sites are modified by SUMO1 and SUMO2/3. Consistently, inhibition of SUMOylation, which compromises APB formation^84^, reduced post-MiDAS in G1, confirming a functional role for SUMOylation and APB formation in promoting post-MiDAS. As APBs are a hallmark of the ALT pathway and their formation is promoted by the ALT helicase BLM^50,53,85^, which co-localizes with nearly all APBs at post-MiDAS sites, our data suggest a mechanistic link between the initiation of ALT in S/G2 and post-MiDAS in G1.

Interestingly, we found that post-MiDAS shares additional key factors and processes with ALT in S/G2 and MiDAS in mitosis, including the ALT replisome components POLD3 and PCNA, and the BIR antagonists FANCM and SLX4, which restrain BLM activity during BIR^28,54,56,58,86^. Furthermore, our findings indicate that post-MiDAS requires POLD3, PCNA, RAD18, and FANCD2, and that post-MiDAS sites are enriched for ubiquitylation. These observations suggest that post-MiDAS shares features with MiDAS, in which RAD18-dependent monoubiquitylation of PCNA promotes the recruitment of TLS polymerases to FANCD2-marked under-replicated DNA, followed by a POLD3-mediated polymerase switch, which enables processive DNA synthesis^64,65^. Thus, key DNA replication factors and BIR regulators acting beyond S-phase appear to be shared across ALT, MiDAS and post-MiDAS in distinct cell cycle phases.

Recent studies demonstrated that the helicase WRN is required for efficient ALT-mediated telomere synthesis in S/G2 and for MiDAS, whereas BLM appears dispensable for MiDAS at CFSs but has been implicated in telomeric MiDAS at clustered telomeres and is a well-established regulator of ALT in S/G2^50–55,87^. According to our data, BLM promotes post-MiDAS, whereas WRN seems to negatively impact EdU incorporation at post-MiDAS sites in G1. Although further experiments are required to corroborate and elucidate the role of WRN during post-MiDAS, these findings support cell cycle phase-specific regulation.

Consistent with this notion, the distinct protein environments in different cell cycle phases suggest that cell cycle phase-specific layers of regulation exist. For instance, MiDAS occurs during high CDK activity in mitosis, with both sister chromatids being present, whereas post-MiDAS in G1 takes place after sister chromatid segregation and under low CDK activity^88^. Such considerations led us to profile candidate post-MiDAS factors proteome-wide using RPA interactomics and proximity labeling proteomics. Consistent across the three complementary proteomics approaches that were employed, the ubiquitin ligase complex APC/C emerged as an interactor of RPA in G1 cells. Together with its co-activator CDH1, the APC/C appears to negatively regulate post-MiDAS in G1. In contrast, the second APC/C co-activator CDC20 showed no significant effect on post-MiDAS in G1 and, unlike CDH1, was not detected in the G1-specific RPA proximity labeling analyses. This is consistent with the established cell cycle-regulated activity of APC/C^CDC20^ at mitotic entry and APC/C^CDH1^ functioning from late mitosis through G1 until the G1/S-transition^66,67^. Thus, APC/C^CDH1^ is unlikely to regulate ALT in S/G2 and MiDAS in early mitosis and might represent a post-MiDAS-specific vulnerability of ALT cancer cells.

Beyond its established role in cell cycle control, APC/C^CDH1^ has emerged as an important tumor suppressor by promoting faithful DNA replication and coordinating cellular responses to DNA damage, and targeting the APC/C has potent anti-tumor capacity^89,90^. Our results align with growing evidence that roles beyond cell cycle regulation are important for the function of APC/C^CDH1^ to protect genome integrity and suggest that APC/C^CDH1^ restrains post-MiDAS activity to limit the excessive accumulation of toxic BIR-intermediates. In this way, APC/C^CDH1^ may safeguard genome stability by keeping post-MiDAS in check. Consistently, excessive ALT activity upon FANCM loss or upon deregulation of the telomeric repeat-containing noncoding RNA TERRA at ALT telomeres was previously shown to compromise telomere maintenance and cell survival^56–58,91^.

Although a full range of APC/C^CDH1^ substrates in the context of post-MiDAS in G1 remains to be identified, our results indicate that TOPBP1 could be a relevant target. TOPBP1 was predicted as a potential APC/C^CDH1^ substrate in silico^75^, and we obtained consistent experimental evidence in multiple ALT-positive cancer cell lines. Together with the elevated RPA32 phosphorylation at serine 33 (S33) at post-MiDAS sites after CDH1 depletion in G1, indicative of non-canonical local ATR activity, these findings support a model in which APC/C^CDH1^ restrains TOPBP1-mediated ATR signaling in G1 to prevent excessive, potentially toxic post-MiDAS activity. Consistently, we observed a survival defect in ALT-positive U-2 OS and SaOS-2 cancer cells upon CDH1 depletion, whereas ALT-negative HeLa and RPE-1 cells were less affected. Thus, targeting APC/C^CDH1^ and potentially other ALT, MiDAS, and post-MiDAS factors may unmask vulnerabilities of ALT-dependent cancers. Indeed, interfering with the ALT regulator FANCM by antisense oligonucleotides (ASOs) or recently developed cell-penetrating peptide (CPP) inhibitors shows promising ALT-specific effects in cells and mouse xenografts^92,93^, indicating a clinical potential for targeted hyper-activation of ALT-associated processes.

## Methods

### Cell culture

All cell lines used in this study were cultured under standard cell culture conditions at 37 °C in a humidified atmosphere in the presence of 5% CO_2_ and were routinely tested for *Mycoplasma* contamination. U-2 OS, U-2 OS FUCCI, U-2 OS GFP-H2B, HeLa, and SW26 cells were cultured in Dulbecco’s modified Eagle’s medium (DMEM, Gibco, 61965-026) supplemented with 10% FBS (Corning, 35-015-CV; Capricorn Scientific, FBS-16A) and 1% penicillin/streptomycin antibiotics (Gibco, 15140-122). GM-847 cells were cultured in Dulbecco’s modified Eagle’s medium (DMEM, Gibco, 61965-026) supplemented with 10% FBS (FBS Good, PAN Biotech, P40-37500) and 1% penicillin/streptomycin antibiotics (Gibco, 15140-122). SaOS-2 and hTERT-RPE-1 cells were cultured in Dulbecco’s modified Eagle’s medium/F-12 Nutrient Mixture (Ham) (DMEM/F12 1:1, Gibco, 31331-028) supplemented with 10% FBS (Corning, 35-015-CV; Capricorn Scientific, FBS-16A) and 1% penicillin/streptomycin antibiotics (Gibco, 15140-122).

Stable U-2 OS TRF1-FokI cells^28^ were maintained in DMEM (Gibco, 61965-026) supplemented with 10% FBS (Corning, 35-015-CV; Capricorn Scientific, FBS-16A), 1% penicillin/streptomycin antibiotics (Gibco, 15140-122) and 1 µg/ml puromycin (Gibco, A11138-03). TRF1-FokI expression was induced with 40 ng/ml doxycycline (Sigma-Aldrich, D9891) for 16 h. Telomeric DSB induction, nuclear translocation of TRF1-FokI and protein stabilization were achieved by treating cells with 1 µM 4-hydroxytamoxifen (4-OHT, Sigma-Aldrich, H7904) and 1 µM Shield-1 (Takara Bio, 632189) for 2 h.

Stable U-2 OS FUCCI TurboID-RPA32-HA cells were maintained in DMEM (Gibco, 61965-026) supplemented with 10% FBS (Corning, 35-015-CV; Capricorn Scientific, FBS-16A), 1% penicillin/streptomycin antibiotics (Gibco, 15140-122) and 400 µg/ml Geneticin (Gibco, 10131-035).

### Drug treatments

Cells were treated as indicated with the following compounds at the following final concentrations, unless stated otherwise. For replication stress induction, cells were treated with 1 µM ATR inhibitor AZ-20 (ATRi, Tocris Bioscience, 5198) for 24 h; 0.2 µM Aphidicolin (APH, Sigma-Aldrich, A0781) for 24 h; or the combination of 1 µM AZ-20 and 0.2 µM APH for 24 h. For acute inhibition, cells were treated with 1 µM SUMO inhibitor ML-792 (SUMOi, Selleckchem, S8697) for 2 h; 20 µM ATR inhibitor VE-821 (ATRi, Sigma-Aldrich, SML1415) or AZ-20 (ATRi, Tocris Bioscience, 5198) for 2 h; 2 µM BLM inhibitor BLM-IN-1 (BLMi, Med Chem Express, HY- 111756) for 2 h; or 1 µM ubiquitin E1 inhibitor TAK-243 (E1i, Med Chem Express, HY-100487) for 4 h.

### Cloning of pcDNA3.1(+)-TurboID-RPA32-HA plasmid

To generate the pcDNA3.1(+)-TurboID-RPA32-HA construct, mCherry-Cry2WT was deleted from the pcDNA5-TurboID-RPA2-mCherry-Cry2WT plasmid^94^ and replaced with an HA.1 tag using primers TurboID-RPA2_mCh_Cry2_del_HA-stop_fw and TurboID-RPA2_mCh_Cry2_del_rev, followed by blunt-end ligation. TurboID-RPA32-HA was then amplified from the resulting pcDNA5-TurboID-RPA2-HA plasmid using primers pcDNA31_to_TurboID-RPA2-GA_fw and pcDNA31_to_TurboID-RPA2_GA_rev, and the pcDNA3.1(+)-flag plasmid was linearized using primers pcDNA3.1_lin_fw and pcDNA3.1_lin_rv. The amplified TurboID-RPA32-HA fragment and the linearized pcDNA3.1(+)-flag vector were subsequently assembled using Gibson assembly and transformed into chemically competent bacteria generated in-house, derived from Library Efficiency^TM^ DH5α^TM^ Competent Cells (Thermo Fisher). Following plasmid isolation, correct cloning was verified by sequencing. All primers used for cloning and their sequences are listed in Supplementary Table 1. They were ordered and obtained from Microsynth AG, Switzerland.

### Generation of U-2 OS FUCCI TurboID-RPA32-HA cells

To generate the stable monoclonal U-2 OS FUCCI TurboID-RPA32-HA cell line, U-2 OS FUCCI cells were transfected with 2.5 µg of the pcDNA3.1(+)-TurboID-RPA32-HA plasmid using Lipofectamine P3000 (Thermo Fisher Scientific, L3000-008) following the manufacturer’s instructions. 48 h after plasmid transfection, 400 µg/ml Geneticin (Gibco, 10131-035) was added to the culture medium for selection. Cells were then seeded by limiting dilution into 96-well plates containing growth medium supplemented with 400 µg/ml Geneticin (Gibco, 10131-035). Individual clones were expanded and screened for expression of the TurboID-RPA32-HA construct.

### Individual siRNA transfections

Individual siRNA transfections were performed with Ambion Silencer Select siRNAs at a final concentration of 25 nM using Lipofectamine RNAiMAX (Thermo Fisher Scientific, 13778150) according to the manufacturer’s instructions. Asynchronously growing cells were transfected for 72 h. For G1-specific siRNA-mediated knockdowns, cells were arrested in G1 by a 24 h treatment with 1 µM Palbociclib (PD-0332991, Selleckchem, S1116) and subsequently transfected for 48 h in the continued presence of 1 µM Palbociclib (PD-0332991, Selleckchem, S1116) and 0.1 µg/ml KaryoMAX Colcemid (Thermo Fisher Scientific, 15212012), to prevent cells escaping the G1 arrest from re-entering G1. Silencer Select Negative Control #1 (s813) and #2 (s814) from Ambion were used as non-targeting controls. The following Ambion Silencer Select siRNAs were used: siBLM (s1999), siCDC20 (s2749), siCDH1 #1 (s27991), siCDH1 #2 (s27992), siFANCD2 (s4988), siFANCM (s33621), siMCM2 (s8587), siPOLD3 (s21044), siRAD18 (s322296), siRMI1 (s36812), siSLX4 (s39052), siTOP3A (s14311), siTOPBP1 (s21824), siTRF2 (s13991), and siWRN (s14909).

### siRNA screen in asynchronous cells

Asynchronously growing U-2 OS cells were transfected in 96-well imaging plates (Ibidi, 8962) for 72 h with a custom-designed siRNA library at a final concentration of 5 nM using HiPerFect Transfection Reagent (Qiagen, 301704) according to the manufacturer’s instructions. Silencer Select Negative Control #1 (s813) and #2 (s814) from Ambion were used as non-targeting controls, and a mock condition (transfection without siRNA) was included as an additional control. All siRNAs included in the siRNA screen are listed in Supplementary Data 5. All phenotypes relevant for the screen were scored in G1 cells identified by QIBC based on their 2 N DNA content and low EdU intensity.

### siRNA screen in G1-synchronized cells

Following a 24 h treatment with 1 µM Palbociclib (PD-0332991, Selleckchem, S1116) to arrest cells in G1, cells were transfected for 48 h in a 96-well imaging plate (Ibidi, 89626) with a custom-designed siRNA library at a final concentration of 5 nM using HiPerFect Transfection Reagent (Qiagen, 301704) according to the manufacturer’s instructions. To ensure G1-specific knockdowns, cells were maintained in the presence of 1 µM Palbociclib (PD-0332991, Selleckchem, S1116) and 0.1 µg/ml KaryoMAX Colcemid (Thermo Fisher Scientific, 15212012) throughout the 48 h siRNA transfection to prevent cells escaping the G1 arrest from completing the cell cycle and re-entering G1. Silencer Select Negative Control #1 (s813) and #2 (s814) from Ambion were used as non-targeting controls, and a mock condition (transfection without siRNA) was included. All siRNAs included in the siRNA screen in G1-synchronized cells are listed in Supplementary Data 1. All phenotypes relevant for the screen were scored in G1 cells identified by QIBC based on their 2 N DNA content and low EdU intensity.

### EdU labeling

Standard pulsed EdU (5-ethynyl-2’-deoxyuridine) labeling was performed by incubating cells with 10 µM EdU (Thermo Fisher Scientific) for 20 min. For the detection of EdU foci in G1-phase, cells were pre-treated with 50 ng/ml nocodazole (Sigma-Aldrich, SML1665) for 1 h and subsequently incubated with 100 µM EdU and 50 ng/ml nocodazole (Sigma-Aldrich, SML1665) for 1 h. The nocodazole was added to prevent G2/M cells from entering G1 during the EdU pulse. For experiments involving G1-specific siRNA-mediated knockdowns, cells were pulse-labeled with 100 µM EdU for 1 h in the continuous presence of 1 µM Palbociclib (PD-0332991, Selleckchem, S1116) and 0.1 µg/ml KaryoMAX Colcemid (Thermo Fisher Scientific, 15212012). To control for EdU staining specificity, 20 µM of the DNA polymerase inhibitor APH (Sigma-Aldrich, A0781) was added during the 1 h EdU pulse to prevent EdU incorporation. The detection of EdU was performed using Click-iT chemistry (Click-iT EdU Alexa Fluor Imaging Kit, Thermo Fisher Scientific) for 30 min according to the manufacturer’s instructions. EdU foci in G1 were scored by QIBC through automated image analysis as well as manually in QIBC-derived G1 cells.

### Immunofluorescence staining

Cells were cultured on sterile 12 mm glass coverslips or 96-well imaging plates (Ibidi, 89626), fixed in 3% formaldehyde (Sigma-Aldrich) in PBS for 15 min, permeabilized in 0.2% Triton-X-100 (Sigma-Aldrich) in PBS for 5 min, and incubated in blocking solution (filtered DMEM containing 10% FBS (Corning) and 0.02% sodium azide (Merck)) for 15 min at room temperature. For BrdU (5-bromo-2′-deoxyuridine) staining, cells were denatured in 2.5 M HCl for 10 min prior to the permeabilization step. For immunofluorescence staining of BLM, PML and PCNA, cells were pre-extracted with ice-cold pre-extraction buffer (20 mM HEPES pH 7.5, 20 mM NaCl, 5 mM MgCl_2_, 300 mM sucrose, 0.5% NP-40, 1 mM DTT, 1x complete protease inhibitor cocktail (Roche, 11873580001), PhosSTOP (Roche)) for 20 min on ice before fixation in 3% formaldehyde (Sigma-Aldrich) in PBS for 20 min at room temperature^95^. EdU Click-iT staining was performed according to the manufacturer’s instructions before primary antibody incubation. All primary antibodies were diluted in blocking solution and incubated for 2 h at room temperature. Secondary antibodies were diluted in blocking solution and incubated for 1 h at room temperature. Cells were counterstained with 0.5 mg/ml 4′,6-diamidino-2-phenylindole dihydrochloride (DAPI, Thermo Fisher Scientific, D1306) in PBS for 10 min at room temperature. Coverslips were briefly washed with ddH_2_O and mounted onto glass slides using Mowiol (Millipore, 475904). To visualize cell nuclei in representative microscopy images, the DAPI channel was included in overlay images. A list of all primary and secondary antibodies used for immunofluorescence staining is provided in Supplementary Table 2.

### Iterative indirect immunofluorescence imaging (4i)

For iterative indirect immunofluorescence imaging (4i)^49^, cells were grown in 96-well imaging plates (Ibidi, 89626), fixed with 4% formaldehyde (Sigma-Aldrich) in PBS for 20 min and permeabilized in 0.2% Triton-X-100 (Sigma-Aldrich) in PBS for 5 min at room temperature. Cells were blocked with sBS buffer (1% bovine serum albumin (Sigma-Aldrich), 150 mM maleimide (Sigma-Aldrich) in PBS, pH 7.4) for 1 h, EdU Click-iT staining was performed according to the manufacturer’s instructions for 30 min and cells were incubated with primary antibody diluted in cBS (1% BSA (Sigma-Aldrich) in PBS, pH 7.4) for 2 h at room temperature. Cells were then incubated with secondary antibody diluted in cBS for 1 h at room temperature. Nuclear staining was performed using 0.5 mg/ml DAPI (Thermo Fisher Scientific, D1306) for 10 min at room temperature. Imaging buffer (700 mM N-acetyl-cysteine (Sigma-Aldrich) in ddH_2_O, pH 7.4) was added before imaging. After the first imaging round, bound antibodies were removed by denaturation with elution buffer (0.5 M glycine (BioSolve Chemicals), 3 M urea (Eurobio Scientific), 3 M guanidine hydrochloride (Sigma-Aldrich), 50 mM TCEP (Sigma-Aldrich) in water, pH 2.5) twice for 10 min each. After three washes with ddH_2_O, cells were again blocked with sBS buffer for 1 h, and the above-described staining steps were repeated for the second staining and imaging round. All primary and secondary antibodies used for 4i are provided in Supplementary Table 2. Imaging was performed using the automated GE InCell Analyzer 2500HS widefield fluorescence imaging system equipped with an GE inverted microscope, a PCO-sCMOS camera system (16-bit, 2048 × 2048 pixels, pixel size 6.5 µm, read-out speed: 272 MHz), a seven-color Solid State Illuminator (SSI), two quad band pass polychroic mirror and single band pass emission filters using the polychroic beam splitter BGOFR_1 (DAPI (excitation BP 390/18, emission BP 432.5/47), FITC (excitation BP 475/28, emission BP 511.5/23), TRITC (excitation BP 542/27, emission BP 587.5/47), Cy5 (excitation BP 632/22, emission BP 676/48)) and a CFI Plan Apo Lambda 20x air objective (NA 0.75, WD 1.0 mm). Images were acquired under non-saturating conditions, with identical acquisition settings applied to all samples within the same experiment.

### Quantitative image-based cytometry (QIBC)

For quantitative image-based cytometry (QIBC)^43,44^, automated multichannel widefield imaging was performed. Images of cell populations were acquired on an Olympus ScanR High-Content Screening System (Olympus ScanR Image Acquisition software v.3.0.1, 3.2.0, 3.3.0, and 3.5) equipped with an inverted motorized Olympus IX83 microscope, a motorized stage, IR-laser hardware autofocus, a fast emission filter wheel with one set of bandpass filters for multi-wavelength acquisition (DAPI (excitation BP 395/25, emission BP 435/26), FITC (excitation BP 470/24, emission BP 515/30), TRITC (excitation BP 555/28, emission BP 595/40), Cy5 (excitation BP 640/30, emission BP 705/72)), a Hamamatsu ORCA-FLASH 4.0 V2 sCMOS camera (12-bit, 2048 x 2048 pixels, pixel size 6.5 x 6.5 µm), and a UPLSAPO 20x air objective (NA 0.75, WD 0.6 mm) or a UPLSAPO 40x air objective (NA 0.9, WD 0.18 mm). Images were acquired under non-saturating conditions, with identical acquisition settings applied to all samples within the same experiment. Image analysis was performed with the built-in Olympus ScanR Analysis software (v.3.0.1, 3.2.0, 3.3.0, 3.4.0 and 3.5.0) involving dynamic background correction, nuclei segmentation using an integrated intensity-based object detection module based on DAPI, and foci segmentation using an integrated spot detection module. For downstream analyses, properly detected interphase nuclei containing a 2N-4N DNA content were gated based on total and mean DAPI intensities. G1-phase nuclei were gated based on their 2 N DNA content (total DAPI intensity) and low mean EdU intensity, as exemplified in Supplementary Figs. 1, 2. Co-localization events in G1-phase nuclei were quantified manually. Similar cell numbers were compared across all samples within the same experiment. Color-coded cell cycle-resolved scatterplots of asynchronously growing cell populations were generated using TIBCO Spotfire (v.7.9.1 and 10.10.1). Mild jittering (random displacement of data points along the discrete data axes) was applied to reduce overlap of individual data points when visualizing discrete data in scatterplots.

### Confocal microscopy

Confocal imaging for telomere clustering analysis was performed on an Olympus IXplore SpinSR10 spinning disk confocal microscope equipped with a YOKOGAWA CSU-W1 50 µm pinhole disk. Sequential laser excitation was performed at 405 nm (50 mW), 488 nm (100 mW), 561 nm (100 mW), and 640 nm (100 mW). Fluorescence emission was detected through the following band-pass filters: DAPI (447/60), FITC (525/50), TRITC (617/73), and Cy5 (685/40), with a 2 x Hamamatsu ORCA-Fusion sCMOS camera system (16-bit, 2304 × 2304 pixels, pixel size 6.5 µm, up to 89.1 frames per second). Z-stacks were acquired with a UPLAN S Apo 40x air objective (NA 0.95, WD 0.18 mm). Images were acquired under non-saturating conditions, with identical acquisition settings applied to all samples within the same experiment. Image analysis was performed with the built-in Olympus ScanR Analysis software (v.3.0.1, 3.2.0, 3.3.0, 3.4.0 and 3.5.0) as described in the QIBC section above.

### Time-lapse imaging

For time-lapse microscopy, cells were seeded and treated in 96-well imaging plates (Ibidi, 89626). Prior to imaging, the growth medium was replaced with FluoroBrite DMEM (Thermo Fisher Scientific, A1896701) supplemented with 10% FBS (Corning), 1% penicillin/streptomycin (Gibco), and GlutaMAX (Gibco). Time-lapse imaging was performed under environmental control for temperature (37 °C) and gas (5% CO_2_ and 20% O_2_) using an automated GE InCell Analyzer 2500HS (version 7.4) widefield fluorescence imaging system equipped with an inverted microscope, a PCO-sCMOS camera system (16-bit, 2048 × 2048 pixels, pixel size 6.5 µm, read-out speed: 272 MHz), a seven-color Solid State Illuminator (SSI), two quad band-pass polychroic mirrors, and single band-pass emission filters using the polychroic beam splitter BGOFR_1 (DAPI (excitation BP 390/18, emission BP 432.5/47), FITC (excitation BP 475/28, emission BP 511.5/23)). Images were acquired with a CFI Plan Apo Lambda 20 x Air objective (NA 0.75, WD 1.0 mm) at 15 min intervals for 24 h under non-saturating conditions, with identical acquisition settings applied to all samples within the same experiment. Image stacks and movie stills were generated using Fiji/ImageJ 64-bit (version 2.00-rc54/1.51 h).

### Fluorescence-activated cell sorting (FACS) of G1-phase cells

U-2 OS FUCCI cells were arrested in G1 by a 24 h treatment with 1 µM Palbociclib (PD-0332991, Selleckchem, S1116) prior to FACS to improve sorting efficiency and reduce sorting time. For DNA content staining, 5 µg/ml Hoechst 33342 (Thermo Fisher Scientific, 62249) was added to the culture medium and cells were incubated for 30 min at 37 °C. Cells were then harvested by trypsinization, resuspended in OptiMEM (Gibco, 11058-021) containing 5 µg/ml Hoechst 33342 (Thermo Fisher Scientific, 62249) and passed through a 40 µm cell strainer (Corning) before FACS sorting on a BD FACSAria III 5 L (FACS Diva Software v.8.0.1) equipped with 405 nm (Hoechst), 488 nm (GFP) and 561 nm (RFP) laser lines and a 100 µm nozzle. G1-phase cells were sorted based on FUCCI fluorescence (RFP-CDT1-positive, GFP-Geminin-negative) combined with Hoechst DNA content staining (2 N). Sorted G1-phase cells were collected in OptiMEM (Gibco, 11058-021) and further processed for downstream applications. For proteomics experiments, more than 55 million G1-phase cells were sorted per experiment. Flow cytometry data were analyzed using FlowJo (version 10.10.0).

### Co-immunoprecipitation

Cells were harvested by trypsinization and washed twice with ice-cold PBS or, in the case of FACS-sorted G1-phase cells, pelleted by centrifugation at 500 x *g* for 5 min. For co-immunoprecipitation from whole-cell extracts, cell pellets were resuspended in TNE buffer with high salt concentration (50 mM Tris-HCl, pH 8.0, 500 mM NaCl, 0.1% Igepal CA630, 1 mM EDTA, 1 x complete protease inhibitor cocktail (Roche, 11873580001), PhosSTOP (Roche, 04906845001)) supplemented with 1 mM MgCl_2_ and 25 U/ml benzonase (Millipore, 70664-10KUN). Chromatin was sheared by passing the lysate 10 times through a 26-gauge needle. Cell lysates were then incubated for 5 min at room temperature and centrifuged at 10000 x *g* for 15 min at 4 °C. For co-immunoprecipitations from chromatin-bound protein extracts, cell pellets were incubated in ice-cold 0.2% Triton X-100 (Sigma-Aldrich) in PBS for 3 min on ice and then centrifuged at 500 x *g* for 10 min at 4 °C before proceeding with lysis as described above. After lysis, cell lysates were diluted in TNE buffer without salt (50 mM Tris-HCl, pH 8.0, 0.1% Igepal CA630, 1 mM EDTA, 1 x complete protease inhibitor cocktail (Roche, 11873580001), PhosSTOP (Roche, 04906845001)) to a final NaCl concentration of 150 mM for co-immunoprecipitation. A total of 800-1000 µg of cell lysate was pre-cleared by incubation with 20 µl equilibrated Protein G Sepharose 4 Fast Flow beads (Cytiva, 17-061801) for 1 h at 4 °C. Following bead removal, the pre-cleared cell lysate was incubated with 1 µg of RPA70 antibody (Abcam, ab79398), 4 µg of APC3 (CDC27) antibody (SantaCruz, sc-9972) or, as a negative control, 1 µg of FLAG M2 antibody (Sigma-Aldrich, F-3165) for 3 h at 4 °C. Antibody-bound protein complexes were precipitated by adding 30 µl of equilibrated Protein G Sepharose 4 Fast Flow beads (Cytiva, 17-061801) and incubating for 1 h at 4 °C. Beads were washed four times with TNE buffer (50 mM Tris-HCl pH 8.0, 150 mM NaCl, 0.1% Igepal CA630, 1 mM EDTA, 1 x complete protease inhibitor cocktail (Roche, 11873580001), PhosSTOP (Roche, 04906845001)) and either processed for liquid chromatography mass spectrometry (LC-MS) or eluted by boiling in 10 x SDS-PAGE loading buffer at 95 °C for 5 min prior to analysis by immunoblotting.

### Sample preparation and RPA70-IP LC-MS analysis

RPA70 and IgG control immunoprecipitations were each performed in quadruplicate from 55 million G1-phase cells per experiment. Captured proteins were digested on Protein G Sepharose 4 Fast Flow beads using 500 ng of sequencing-grade modified Trypsin (Promega, V5111), including reduction and alkylation of cysteines by Tris(2-carboxyethyl)phosphine hydrochloride (TCEP) and 2-Chloroacetamide (ClAA) addition, respectively. LC-MS analysis of the resulting peptide mixture was conducted on an Orbitrap Exploris 480 Mass Spectrometer (Thermo Fisher Scientific) directly coupled by electrospray ionization (ESI) to an ACQUITY UPLC M-Class System (Waters) configured for 75-micron scale single-pump trapping. Peptides were separated on a nanoEase HSS C18 T3, 100 Å, 75 µm x 250 mm analytical column (Waters, PN: 186008818) at a constant flow rate of 300 nl/min applying a piecewise linear gradient from 5 to 32% solvent B in 45 min (solvent A: water incl. 0.1% formic acid, solvent B: acetonitrile incl. 0.1% formic acid). MS data acquisition was conducted in a data-dependent mode. Data-dependent scans (MS2) were acquired in the Orbitrap mass analyzer at 30’000 resolution (norm. AGC target: 100%, maxIT: 119 ms) covering precursor ions in the scan range 350–1200 m/z and in charge states 2 to 7. Precursors were quadrupole isolated at 1.2 m/z and HCD fragmented at an NCE of 30. Precursor scans (MS1) were conducted in the Orbitrap mass analyzer at 120’000 resolution (AGC target: 300%, maxIT: 50 ms) with lock mass correction.

### RPA70-IP LC-MS data analysis

LC-MS data were processed using a scripted Philosopher workflow^96^ followed by label-free quantification using IonQuant^97^. In short, MS2 spectra were searched against the UniProt Homo sapiens reference proteome (UP000005640, taxonomy 9606) using the MSFragger search engine (version 3.4), allowing for one missed tryptic cleavage and fixed carbamidomethylation of cysteine, variable methionine oxidation, and variable acetylation of the protein N-terminus after methionine removal. Label-free quantification was performed using the FDR-controlled match-between-runs algorithm as implemented by IonQuant (version 1.7.17). Affinity enrichment analysis was performed using proLFQua^98^ by fitting a SAINT^99^ model to the protein abundance estimates. Prey proteins were filtered using a Bayesian FDR cutoff of 5% and an empirical fold-change cutoff of 2 to compile protein-protein interactions. Protein network analysis was performed with Cytoscape (version 3.10.3).

### Preparation of whole-cell protein extracts for immunoblotting

Cells were harvested by trypsinization and washed twice with ice-cold PBS. Cell pellets were resuspended in ice-cold RIPA buffer (50 mM Tris-HCl, pH 8.0, 150 mM NaCl, 1% NP-40, 0.1% sodium deoxycholate) supplemented with 1x complete protease inhibitor cocktail (Roche, 11873580001), PhosSTOP (Roche, 04906845001), 2 mM MgCl_2_, and 25 U/ml benzonase (Millipore, 70664-10KUN). Lysates were incubated for 5 min at room temperature and centrifuged at 15000 x *g* for 15 min at 4 °C. Protein concentrations were determined using the Bradford assay (Bio-Rad, 5000006) according to the manufacturer’s instructions.

### Western blotting

Cell protein extracts were separated by standard SDS-PAGE and transferred onto 0.45 µm PVDF membranes (Millipore, IPFL85R). After blocking with 5% milk in PBS-T (PBS + 0.1% Tween-20), membranes were incubated with primary antibodies diluted in 5% milk in PBS-T for 3 h at room temperature or overnight at 4 °C. Membranes were then washed three times with PBS-T and incubated with HRP-conjugated secondary antibodies diluted in 1% milk in PBS-T for 1 h at room temperature. For biotin detection, membranes were blocked with 2.5% BSA (Sigma-Aldrich) in PBS-T and incubated with Streptavidin-HRP antibody (Abcam, ab7403, 1:3700) diluted in 2.5% BSA in PBS-T for 3 h at room temperature. Membranes were washed three times with PBS-T, and protein signals were detected using Pierce ECL Western Blotting Substrate (Thermo Fisher Scientific, 32106) with either an OPTIMAX X-Ray Film Processor (PROTEC Medizintechnik GmbH & Co. KG) or a digital Fusion FX7 Absolute Chemiluminescence Imaging System, Model 2025 (Vilber). All primary and secondary antibodies used for Western blotting are listed in Supplementary Table 2.

### Proximity labeling by TurboID

U-2 OS FUCCI TurboID-RPA32-HA cells were arrested in G1 by a 24 h treatment with 1 µM Palbociclib (PD-0332991, Selleckchem, S1116). 30 min prior to biotin labeling, 50 ng/ml nocodazole (Sigma-Aldrich, SML1665) was added to prevent G2/M cells from entering G1 during the biotin labeling. Biotin labeling with 500 µM biotin (Sigma-Aldrich) was then performed for 4 h at 37 °C in the constant presence of 1 µM Palbociclib (PD-0332991, Selleckchem, S1116) and 50 ng/ml nocodazole (Sigma-Aldrich, SML1665). For downstream analysis of G1-phase cells, 5 µg/ml Hoechst 33342 (Thermo Fisher Scientific, 62249) was added to the culture medium during the final 30 min of biotin labeling, and 80 million G1-phase cells were subsequently FACS-sorted based on FUCCI fluorescence (RFP-CDT1-positive, GFP-Geminin-negative) and Hoechst DNA content staining (2 N) as described above.

For nuclear protein extractions, pelleted cells were resuspended in Buffer A (10 mM EDTA, 10 mM KCl, 10 mM HEPES, 1 mM DTT, 0.1% NP-40, 1 x complete protease inhibitor cocktail (Roche, 11873580001)), incubated for 10 min on ice and centrifuged at 10000 x *g* for 10 min at 4 °C. The nuclear pellet was then resuspended in Buffer B (0.85 mM EDTA, 340 mM NaCl, 17 mM HEPES, 0.85% glycerol, 1 mM DTT, 1 x complete protease inhibitor cocktail (Roche, 11873580001)), incubated for 2 h with shaking, and centrifuged at 21100 x *g* for 10 min at 4 °C. The supernatant containing the nuclear protein fraction was collected, and the protein concentration was determined using the Bradford assay (Bio-Rad, 5000006) according to the manufacturer’s instructions.

Equilibrated Dynabeads M-280 Streptavidin beads (Thermo Fisher Scientific, 10003D) were incubated with 150 µg nuclear extract in binding buffer (20 mM HEPES, 2 mM MgCl_2_, 20% glycerol, 0.2 mM EDTA, 0.5% NP-40, 150 mM NaCl, 0.5 mM DTT, 1x complete protease inhibitor cocktail (Roche, 11873580001)) at 4 °C overnight. Beads were washed twice in binding buffer, twice in PBS containing 0.5% NP-40, and twice in PBS. Beads were then either processed for liquid chromatography mass spectrometry (LC-MS) or eluted by boiling in 10 x SDS-PAGE loading buffer at 95 °C for 5 min prior to analysis by immunoblotting.

### Sample preparation and TurboID-RPA32 proximity labeling LC-MS analysis

TurboID-RPA32 proximity labeling samples with or without biotin labeling were prepared in duplicate from 40 million G1 cells per condition. Captured proteins were digested on beads using 500 ng of sequencing-grade modified Trypsin (Promega, V5111), including reduction and alkylation of cysteines by Tris(2-carboxyethyl)phosphine hydrochloride (TCEP) and 2-Chloroacetamide (ClAA) addition, respectively. LC-MS analysis of the resulting peptide mixture was conducted on an Orbitrap Exploris 480 Mass Spectrometer (Thermo Fisher Scientific) directly coupled to an ACQUITY UPLC M-Class System (Waters) configured for 75-micron scale single-pump trapping. Peptides were separated on a nanoEase HSS C18 T3, 100 Å, 75 µm x 250 mm analytical column (Waters, PN: 186008818) at a constant flow rate of 300 nl/min applying a piecewise linear gradient from 5 to 33% solvent B in 45 min (solvent A: water incl. 0.1% formic acid, solvent B: acetonitrile incl. 0.1% formic acid). MS data acquisition was conducted in data-independent mode. DIA scans were acquired in the Orbitrap mass analyzer at 15,000 resolution (norm. AGC target: 3000%, maxIT: 23 ms) covering the m/z range from 350 to 1050 in 70 non-overlapping isolation windows. Precursors were quadrupole isolated at 10 m/z and HCD fragmented at a NCE of 28 (default charge 3).

### TurboID-RPA32 proximity labeling LC-MS data analysis

LC-MS data were processed using DIA-NN (version 1.8.2) in library-free mode, including match-between-runs^100^. Spectral library prediction was initiated from the UniProt Homo sapiens reference proteome (UP000005640, taxonomy 9606), allowing for fully tryptic peptides of 6 to 30 amino acids, including fixed carbamidomethylation of cysteine and variable methionine oxidation (UniMod #35), and precursor charge states of 2 or 3. Quantification results were filtered using a 1% FDR cutoff. For interaction scoring, protein abundance estimates were generated by proLFQua^98^ based on the DIA-NN main report (minimum of 2 peptides per protein) and forwarded to SAINTexpress^101^. Potential prey proteins were filtered using a Bayesian FDR cutoff of 5% and an empirical fold-change cutoff of 1.9.

### Gene ontology analysis

Gene ontology (GO) analysis of the overlapping significantly enriched proteins identified by proteomics as RPA70 interactors (EFC ≥ 2; BFDR ≤ 0.05) and TurboID-RPA32 interactors (EFC ≥ 1.9; BFDR ≤ 0.05, peptide count ≥ 4) was performed with the ShinyGO tool (version 0.85.1)^102^ for cellular component, using all protein-coding genes in the human genome as the background.

### Colony formation assay

For colony formation assays, cells were seeded at a density of 300 cells (U-2 OS, HeLa, and RPE-1) or 1200 cells (SaOS-2) per well into 6-well plates 24 h after siRNA transfection. All conditions were plated in triplicate per biological replicate. After 7 days (U-2 OS, HeLa, and RPE-1) or 14 days (SaOS-2) of growth, cells were washed twice with PBS and stained with crystal violet solution (0.5% crystal violet in 20% ethanol) for 20 min at room temperature. After staining, cells were rinsed with ddH_2_O and air-dried at room temperature. Colony formation was quantified manually.

### RNA extraction, reverse transcription and quantitative PCR

RNA was purified using TRIzol reagent (Life Technologies). The obtained RNA was primed with random hexamers (Roche) and reverse-transcribed into cDNA using MultiScribe Reverse Transcriptase (Thermo Fisher Scientific). The qPCRs were performed with the KAPA SYBR FAST qPCR Kit (KAPA Biosystems) on a Rotor-Gene Q system (Qiagen) using Rotor-Gene Q Series Software (version 20.4.21). Samples were run in triplicate, normalized to RPS12, and the results are presented as relative fold changes. The qPCR primers were designed against exon-exon boundaries, whenever possible, validated by siRNA-mediated target knockdown, and their sequences are provided in Supplementary Table 3. They were ordered and obtained from Microsynth AG, Switzerland.

### Statistics and reproducibility

Unless specified in the figure legends, all microscopy images and Western blot data are representative of at least 3 independent experiments. Statistical analyses were performed using GraphPad Prism (versions 9 and 10). Data are presented as the mean ± standard deviation (SD) or, for violin plots, the median, unless stated otherwise. Sample sizes and the statistical tests applied are specified in the figure legends and provided below for all figure panels. The following *p*-values were considered statistically significant: * *p* ≤ 0.05, ** *p* ≤ 0.01, *** *p* ≤ 0.001, and **** *p* ≤ 0.0001.

Statistical tests, *p*-values, and confidence intervals (CIs) for individual figure panels are as follows: Fig. 1e: Two-tailed unpaired *t* test, *p* < 0.0001 and CI = 0.5744 to 0.7816. Fig. 1f: Two-tailed unpaired *t* test, *p* = 0.0015 and CI = 0.1354 to 0.5686. Fig. 1i: Two-tailed unpaired *t* test, *p* = 0.0209 and CI = 1.912 to 13.49. Fig. 1j: Two-tailed unpaired *t* test, *p* = 0.0038 and CI = 1.087 to 2.929. Fig. 1l: Two-tailed unpaired *t* test, *p* < 0.0001 and CI = 1.645 to 3.235. Fig. 2b: Two-tailed unpaired *t* test; *p* = 0.9888 and CI = − 6.833 to 6.907. Fig. 2c: Two-tailed unpaired *t* test; *p* = 0.0029 and CI = 0.3371 to 0.8384. Fig. 2e: Two-tailed unpaired *t* test; *p* = 0.1955 and CI = −1.702 to 6.022. Figure 2f: Two-tailed unpaired *t* test, *p* = 0.0010 and CI = 0.4063 to 0.7937. Figure 3b: One-way ANOVA with Dunnett’s multiple-comparison test; *p* = 0.0083 and CI = − 3.856 to − 0.5145 (1-3 EdU foci, siSLX4); *p* = 0.0043 and CI = − 13.08 to − 2.264 ( ≥ 3 EdU foci, siSLX4); *p* = 0.0012 and CI = − 4.411 to − 1.070 (1-3 EdU foci, siWRN); *p* = 0.0018 and CI = − 13.86 to − 3.004 ( ≥ 3 EdU foci, siWRN); *p* < 0.0001 and CI = − 7.708 to − 4.366 (1-3 EdU foci, siFANCM); *p* = 0.0003 and CI = − 15.41 to − 4.598 ( ≥ 3 EdU foci, siFANCM); *p* = 0.0123 and CI = 0.4034 to 3.745 (1-3 EdU foci, siTOP3A); *p* = 0.0062 and CI = 1.924 to 12.74 ( ≥ 3 EdU foci, siTOP3A); *p* = 0.0447 and CI = 0.03302 to 3.374 (1–3 EdU foci, siRMI1); *p* = 0.0110 and CI = 1.408 to 12.22 ( ≥ 3 EdU foci, siRMI1); *p* = 0.0207 and CI = 0.2552 to 3.597 (1-3 EdU foci, siBLM); *p* = 0.0106 and CI = 1.441 to 12.25 ( ≥ 3 EdU foci, siBLM); *p* = 0.0123 and CI = 0.4034 to 3.745 (1-3 EdU foci, siPOLD3); *p* = 0.0005 and CI = 4.33 to 15.14 ( ≥ 3 EdU foci, siPOLD3). Figure 3e: Two-tailed unpaired *t* test; *p* = 0.0538 and CI = − 0.1947 to 15.08. Figure 3f: Two-tailed unpaired *t* test; *p* = 0.0109 and CI = 0.7854 to 3.328. Figure 4a: One-way ANOVA with Tukey’s multiple-comparison test; *p* < 0.0001 and CI = − 616.3 to − 286.7 (untreated); *p* < 0.0001 and CI = − 685.1 to − 355.5 (APH + ATRi). Figure 4b: Two-tailed unpaired *t* test; *p* < 0.0001 and CI = 219.6 to 344.9. Figure 4e: Two-tailed unpaired *t* test; *p* = 0.0434 and CI = 0.4397 to 17.77. Figure 4f: Two-tailed unpaired *t* test; *p* = 0.0386 and CI = 0.5252 to 11.8. Figure 4g: Two-tailed unpaired *t* test; *p* = 0.2051 and CI = −5.979 to 20.28. Figure 4h: Two-tailed unpaired *t* test; *p* = 0.5449 and CI = − 2.803 to 1.725. Figure 4i: Two-tailed unpaired *t* test; *p* = 0.9134 and CI = − 9.152 to 9.949. Figure 4j: One-way ANOVA with Tukey’s multiple-comparison test; *p* < 0.0001 and CI = − 2360 to − 831.2 (untreated all PML foci vs. untreated EdU-positive PML foci); *p* < 0.0001 and CI = − 3257 to − 1728 (APH+ATRi all PML foci vs. APH+ATRi EdU-positive PML foci); *p* < 0.0001 and CI = − 2178 to − 648.8 (untreated EdU-positive telomeres vs. APH+ATRi EdU-positive telomeres). Figure 4m: Two-tailed unpaired *t* test; *p* = 0.0697 and CI = − 0.6956 to 11.51. Figure 4n: Two-tailed unpaired *t* test; *p* = 0.0331 and CI = 0.8489 to 12.18. Figure 4o: Two-tailed unpaired *t* test; *p* = 0.1941 and CI = − 2.003 to 7.131. Figure 4p: Two-tailed unpaired *t* test; *p* = 0.0159 and CI = 2.193 to 12.01. Fig. 4q: Two-tailed unpaired *t* test; *p* = 0.0056 and CI = − 0.5696 to − 0.1845. Fig. 5a: Two-tailed unpaired *t* test; *p* < 0.0001 and CI = − 0.5254 to − 0.3316. Fig. 5c: Two-tailed unpaired *t* test; *p* = 0.4319 and CI = − 9.159 to 17.56. Fig. 5d: Two-tailed unpaired *t* test; *p* = 0.0121 and CI = 0.6874 to 3.106. Fig. 5f: Two-tailed unpaired *t* test; *p* = 0.6839 and CI = − 4.977 to 6.843. Fig. 5g: Two-tailed unpaired *t* test; *p* = 0.0009 and CI = 11.23 to 21.55. Fig. 5h: Two-tailed unpaired *t* test; *p* < 0.0001 and CI = − 0.2774 to − 0.1366. Fig. 5j: Two-tailed unpaired *t* test; *p* = 0.1359 and CI = − 2.860 to 14.54. Fig. 5k: Two-tailed unpaired *t* test; *p* = 0.0228 and CI = 0.04033 to 0.3130. Fig. 5l: Two-tailed unpaired *t* test; *p* < 0.0001 and CI = − 0.5191 to − 0.2774. Fig. 7b: One-way ANOVA with Tukey’s multiple-comparison test; *p* < 0.0001 and CI = − 0.8412 to − 0.3454 (siCDH1); *p* = 0.3471 and CI = − 0.3946 to 0.1012 (siCDC20). Fig. 7c: One-way ANOVA with Tukey’s multiple-comparison test; *p* = 0.0005 and CI = −0.8494 to −0.1973 (siCDH1 #1); *p* = 0.0017 and CI = − 0.8060 to − 0.1540 (siCDH1 #2). Fig. 7f: Two-tailed unpaired *t* test; *p* < 0.0001 and CI = 9.908 to 13.02. Fig. 7g: Two-tailed unpaired *t* test; *p* < 0.0001 and CI = 3.477 to 4.831. Fig. 7i: Two-tailed unpaired *t* test; *p* = 0.1885 and CI = −5.460 to 19.96. Fig. 7j: Two-tailed unpaired *t* test; *p* = 0.0040 and CI = 1.783 to 4.884. Fig. 7k: Two-tailed unpaired *t* test; *p* = 0.0014 and CI = 361.3 to 1480. Fig. 8b: Two-tailed unpaired *t* test; *p* = 0.8661 and CI = − 6.380 to 5.604. Fig. 8c: Two-tailed unpaired *t* test; *p* = 0.0036 and CI = 2.076 to 5.524. Fig. 8d: Two-tailed unpaired *t* test; *p* < 0.0001 and CI = − 1.226 to − 0.8194. Fig. 8f: Two-tailed unpaired *t* test; *p* = 0.0008 and CI = 0.6501 to 2.470. Fig. 8g: Two-way ANOVA with Šídák’s multiple-comparisons test; *p* < 0.0001 and CI = − 5.102 to − 2.598 (U-2 OS); *p* < 0.0001 and CI = − 4.180 to − 1.676 (SaOS-2); *p* = 0.9866 and CI = − 1.452 to 1.051 (HeLa); *p* = 0.9973 and CI = − 1.383 to 1.121 (RPE-1). Fig. 8i: Two-way ANOVA with Šídák’s multiple-comparisons test; *p* = 0.0001 and CI = 16.54 to 47.13 (U-2 OS); *p* < 0.0001 and CI = 36.30 to 66.90 (SaOS-2); *p* = 0.9311 and CI = − 19.17 to 11.42 (HeLa); *p* = 0.3400 and CI = − 5.730 to 24.86 (RPE-1). Supplementary Fig. 3b: Two-tailed unpaired *t* test; *p* < 0.0001 and CI = 16.06 to 18.44. Supplementary Fig. 3c: Two-tailed unpaired *t* test; *p* < 0.0001 and CI = 29.69 to 32.28. Supplementary Fig. 3d: Two-tailed unpaired *t* test; *p* = 0.0018 and CI = 1.007 to 2.224. Supplementary Fig. 4b: One-way ANOVA with Tukey’s multiple-comparison test; *p* < 0.0001 and CI = − 3.462 to − 1.228 (24 h Palbociclib); *p* < 0.0001 and CI = − 4.607 to − 2.374 (72 h Palbociclib + 48 h Colcemid); *p* < 0.0001 and CI = − 10.76 to − 8.530 (2x Thymidine); *p* < 0.0001 and CI = − 43.74 to − 41.51 (24 h HU); *p* < 0.0001 and CI = − 26.54 to − 24.41 (24 h APH). Supplementary Fig. 4e: One-way ANOVA with Tukey’s multiple-comparison test; *p* < 0.0001 and CI = 13.44 to 16.32 (Mock vs. siTRF2); *p* < 0.0001 and CI = 14.16 to 17.03 (Control #1 vs. siTRF2); *p* < 0.0001 and CI = 14.17 to 17.04 (Control #2 vs. siTRF2). Supplementary Fig. 5a: One-way ANOVA with Dunnett’s multiple-comparison test; *p* = 0.0003 and CI = − 0.2592 to − 0.05638 (siSLX4); *p* < 0.0001 and CI = − 0.3403 to − 0.1375 (siWRN); *p* < 0.0001 and CI = − 0.4547 to − 0.2519 (siFANCM); *p* = 0.0045 and CI = 0.03009 to 0.2329 (siTOP3A); *p* = 0.0155 and CI = 0.01564 to 0.2184 (siRMI1); *p* = 0.0051 and CI = 0.02861 to 0.2341 (siBLM); *p* < 0.0001 and CI = 0.07416 to 0.2769 (siPOLD3). Supplementary Fig. 5b: Two-tailed unpaired *t* test; *p* < 0.0001 and CI = − 0.6677 to − 0.5847. Supplementary Fig. 5c: Two-tailed unpaired *t* test; *p* < 0.0001 and CI = − 1.069 to − 0.8872. Supplementary Fig. 5d: Two-tailed unpaired *t* test; *p* < 0.0001 and CI = − 0.8859 to −0.6947. Supplementary Fig. 5e: Two-tailed unpaired *t* test; *p* < 0.0001 and CI = − 0.8680 to −0.7482. Supplementary Fig. 5f: Two-tailed unpaired *t* test; *p* = 0.0001 and CI = − 1.152 to − 0.7716. Supplementary Fig. 5g: Two-tailed unpaired *t* test; *p* = 0.0001 and CI = − 1.118 to − 0.7640. Supplementary Fig. 5h: Two-tailed unpaired *t* test; *p* < 0.0001 and CI = − 1.042 to −0.9037. Supplementary Fig. 5i: One-way ANOVA with Tukey’s multiple-comparison test; *p* < 0.0001 and CI = − 1.912 to −1.100 (siControl vs. siFANCM); *p* < 0.0001 and CI = 0.2987 to 1.111 (siControl vs. siBLM); *p* < 0.0001 and CI = 0.4987 to 1.311 (siControl vs. siFANCM + siBLM); *p* < 0.0001 and CI = − 2.005 to 2.817 (siFANCM vs. siFANCM+siBLM). Supplementary Fig. 5j: One-way ANOVA with Tukey’s multiple-comparison test; *p* < 0.0001 and CI = − 1.269 to − 0.6578 (siControl vs. siFANCM); *p* = 0.0049 and CI = 0.1673 to 0.7784 (siControl vs. siBLM); *p* = 0.0064 and CI = 0.1467 to 0.7577 (siControl vs. siFANCM+siBLM); *p* < 0.0001 and CI = 1.110 to 1.721 (siFANCM vs. siFANCM+siBLM). Supplementary Fig. 5k: One-way ANOVA with Tukey’s multiple-comparison test; *p* < 0.0001 and CI = 0.7075 to 0.8099 (siFANCM); *p* < 0.0001 and CI = 0.6958 to 0.7983 (siFANCM + siBLM). Supplementary Fig. 5l: One-way ANOVA with Tukey’s multiple-comparison test; *p* < 0.0001 and CI = 0.7795 to 1.072 (siBLM); *p* < 0.0001 and CI = 0.8083 to 1.100 (siFANCM + siBLM). Supplementary Fig. 5m: Two-tailed unpaired *t* test; *p* < 0.0001 and CI = − 0.3035 to − 0.2254. Supplementary Fig. 6a: One-way ANOVA with Tukey’s multiple-comparison test; *p* = 0.0002 and CI = − 1.361 to − 0.5713 (untreated all telomeres vs. untreated EdU-positive telomeres); *p* < 0.0001 and CI = − 1.902 to − 1.113 (APH+ATRi all telomeres vs. APH + ATRi EdU-positive telomeres). Supplementary Fig. 6b: Two-tailed unpaired *t* test; *p* = 0.0192 and CI = 0.5588 to 3.604. Supplementary Fig. 6c: One-way ANOVA with Tukey’s multiple-comparison test; *p* = 0.0013 and CI = − 1.592 to − 0.5000 (untreated all PML foci vs. untreated PML foci at post-MiDAS sites); *p* < 0.0001 and CI = − 2.131 to − 1.039 (APH+ATRi all PML foci vs. APH + ATRi PML foci at post-MiDAS sites). Supplementary Fig. 6d: Two-tailed unpaired *t* test; *p* < 0.0001 and CI = − 93.35 to − 65.90. Supplementary Fig. 6e: Two-tailed unpaired *t* test; *p* < 0.0001 and CI = − 233.9 to − 208.6. Supplementary Fig. 6f: Two-tailed unpaired *t* test; *p* < 0.0001 and CI = − 8.003 to − 6.547. Supplementary Fig. 6g: Two-tailed unpaired *t* test; *p* < 0.0001 and CI = − 37.59 to − 34.71. Supplementary Fig. 6h: Two-tailed unpaired *t* test; *p* < 0.0001 and CI = − 4.782 to − 3.988. Supplementary Fig. 7a: Two-tailed unpaired *t* test; *p* = 0.0006 and CI = − 0.9466 to − 0.5343. Supplementary Fig. 7b: Two-tailed unpaired *t* test; *p* < 0.0001 and CI = − 10.85 to − 7.958. Supplementary Fig. 7c: Two-tailed unpaired *t* test; *p* < 0.0001 and CI = − 1.016 to − 0.9147. Supplementary Fig. 7e: Two-tailed unpaired *t* test; *p* < 0.0001 and CI = − 184.6 to − 172.6. Supplementary Fig. 7f: Two-tailed unpaired *t* test; *p* < 0.0001 and CI = − 18.60 to − 16.34. Supplementary Fig. 7g: Two-tailed unpaired *t* test; *p* = 0.0012 and CI = − 0.9037 to − 0.4490. Supplementary Fig. 7h: Two-tailed unpaired *t* test; *p* = 0.0001 and CI = − 0.9688 to − 0.6570. Supplementary Fig. 7i: Two-tailed unpaired *t* test; *p* = 0.0002 and CI = − 0.5468 to − 0.3470. Supplementary Fig. 7j: Two-tailed unpaired *t* test; *p* < 0.0001 and CI = − 0.3959 to − 0.2850. Supplementary Fig. 7k: Two-tailed unpaired *t* test; *p* = 0.0012 and CI = − 1.299 to − 0.6425. Supplementary Fig. 9c: Two-tailed unpaired *t* test; *p* < 0.0001 and CI = 1.701 to 2.693. Supplementary Fig. 10a: One-way ANOVA with Tukey’s multiple-comparison test; *p* = 0.0004 and CI = − 2.389 to − 1.102 (CDH1); *p* = 0.2011 and CI = − 1.056 to 0.2310 (CDC20). Supplementary Fig. 10b: One-way ANOVA with Tukey’s multiple-comparison test; *p* = 0.0115 and CI = − 0.6239 to − 0.1076 (CDH1); *p* = 0.8810 and CI = − 0.2989 to 0.2174 (CDC20). Supplementary Fig. 10c: Two-tailed unpaired *t* test; *p* = 0.0003 and CI = −1.116 to − 0.6816. Supplementary Fig. 10d: Two-tailed unpaired *t* test; *p* < 0.0001 and CI = − 1.055 to − 0.7535. Supplementary Fig. 10f: One-way ANOVA with Tukey’s multiple-comparison test; *p* = 0.0055 and CI = − 2.126 to − 0.5236 (CDH1 #1); *p* = 0.0044 and CI = − 2.183 to − 0.5814 (CDH1 #2). Supplementary Fig. 10h: Two-tailed unpaired *t* test; *p* = 0.0028 and CI = 0.2899 to 0.7181. Supplementary Fig. 10i: Two-tailed unpaired *t* test; *p* = 0.0027 and CI = 1.133 to 2.781. Supplementary Fig. 10j: Two-tailed unpaired *t* test; *p* = 0.0018 and CI = 0.3900 to 0.8579. Supplementary Fig. 10k: Two-tailed unpaired *t* test; *p* = 0.1431 and CI = −0.5773 to 0.1203. Supplementary Fig. 10l: Two-tailed unpaired *t* test; *p* = 0.2576 and CI = −0.4292 to 0.1528. Supplementary Fig. 10n: One-way ANOVA with Tukey’s multiple-comparison test; *p* < 0.0001 and CI = − 7.055 to − 4.572 (CDH1 #1); *p* < 0.0001 and CI = − 12.56 to − 10.08 (CDH1 #2). Supplementary Fig. 10o: One-way ANOVA with Tukey’s multiple-comparison test; *p* < 0.0001 and CI = − 3.121 to −1.468 (CDH1 #1); *p* < 0.0001 and CI = − 5.153 to − 3.500 (CDH1 #2). Supplementary Fig. 10p: One-way ANOVA with Tukey’s multiple-comparison test; *p* = 0.0015 and CI = − 1404 to − 276.1 (CDH1 #1); *p* = 0.0002 and CI = − 1527 to − 398.7 (CDH1 #2). Supplementary Fig. 11b: Two-tailed unpaired *t* test; *p* < 0.0001 and CI = 0.7080 to 1.052. Supplementary Fig. 11c: Two-tailed unpaired *t* test; *p* < 0.0001 and CI = 0.4351 to 1.073. Supplementary Fig. 11e: Two-tailed unpaired *t* test; *p* < 0.0001 and CI = 0.3279 to 0.8150. Supplementary Fig. 11f: Two-tailed unpaired *t* test; *p* = 0.0011 and CI = 0.5566 to 2.209. Supplementary Fig. 11h: Two-tailed unpaired *t* test; *p* < 0.0001 and CI = 0.9604 to 1.622. Supplementary Fig. 11i: Two-tailed unpaired *t* test; *p* = 0.0008 and CI = 0.3066 to 1.162. Supplementary Fig. 12a: Two-tailed unpaired *t* test; *p* < 0.0001 and CI = − 10.07 to − 9.726. Supplementary Fig. 12b: Two-tailed unpaired *t* test; *p* = 0.0003 and CI = − 0.9998 to − 0.6135. Supplementary Fig. 12c: One-way ANOVA with Šídák’s multiple-comparisons test; *p* < 0.0001 and CI = − 5.887 to − 2.563 (siControl untreated vs. siFANCM untreated); *p* < 0.0001 and CI = 2.755 to 6.079 (siFANCM untreated vs. siFANCM + AZ-20); *p* < 0.0001 and CI = 2.871 to 6.195 (siFANCM untreated vs. siFANCM + VE-821). Supplementary Fig. 12d: One-way ANOVA with Šídák’s multiple-comparisons test; *p* < 0.0001 and CI = − 3.300 to − 1.480 (siControl untreated vs. siFANCM untreated); *p* = 0.0039 and CI = 0.3204 to 2.140 (siFANCM untreated vs. siFANCM + AZ-20); *p* = 0.0015 and CI = 0.4204 to 2.240 (siFANCM untreated vs. siFANCM + VE-821). Supplementary Fig. 12e: One-way ANOVA with Šídák’s multiple-comparisons test; *p* = 0.0002 and CI = − 1.981 to − 0.8778 (siControl untreated vs. siFANCM untreated); *p* = 0.0298 and CI = 0.06389 to 1.167 (siFANCM untreated vs. siFANCM + AZ-20); *p* = 0.0103 and CI = 0.2008 to 1.304 (siFANCM untreated vs. siFANCM + VE-821). Supplementary Fig. 12f: Two-tailed unpaired *t* test; *p* = 0.0181 and CI = 0.07995 to 0.4887.

### Reporting summary

Further information on research design is available in the Nature Portfolio Reporting Summary linked to this article.

## Supplementary information

Supplementary Information

Description of Additional Supplementary Files

Supplementary Data 1

Supplementary Data 2

Supplementary Data 3

Supplementary Data 4

Supplementary Data 5

Supplementary Movie 1

Supplementary Movie 2

Supplementary Movie 3

Reporting Summary

Transparent Peer Review file

## Source data

Source data

## Data Availability

The mass spectrometry proteomics data generated in this study have been deposited to the ProteomeXchange Consortium via the PRIDE^103^ partner repository with the dataset identifiers PXD069592 (RPA70-IP LC-MS from chromatin-bound protein extracts) PXD069739 (RPA70-IP LC-MS from whole-cell protein extracts) and PXD069839 (TurboID-RPA32 proximity labeling LC-MS). Source data are provided in this paper.
