# Supplementary material for: Characterization of post-mitotic DNA synthesis and its regulation in human cells
Source: Nat Commun. 2026 Sep 28;17:11186. doi: 10.1038/s41467-026-78035-5 (PMC13713988; doi:10.1038/s41467-026-78035-5)
Supplement: Supplementary file 2 [file 41467_2026_78035_MOESM12_ESM.pdf]

# Peer Review File

## Characterization of post-mitotic DNA synthesis and its regulation in human cells

Corresponding Author: Dr Matthias Altmeyer

**This file contains all reviewer reports in order by version, followed by all author rebuttals in order by version.**

Version 0:

Reviewer comments:

Reviewer #1

(Remarks to the Author)
In the manuscript by Vornberger et al entitled 'Cellular determinants of replication stress-induced post-MiDAS', the authors aimed to identify genomic regions that undergo what they term 'post-MiDAS' and the cellular factors involved. Towards this goal, the authors performed most of their analysis in an ALT cell line (U2OS), as their previous study showed that post-MIDAS is most frequently found at telomeres of ALT-positive cancer cells (PMID: 34158486).

In the first section of the results, using IF-based methods to detect G1 phase DNA synthesis (labeled by EdU) and its co-localization with TRF1 or CREST, the authors concluded that both telomeres and centromeres are post-MiDAS regions. This analysis has two key weaknesses: 1) It is a candidate approach, and therefore other EdU foci that do not co-localize with TRF1 or CREST were overlooked. It was surprising that the authors did not use a well-established and unbiased method to detect all of the regions labelled with EdU in G1 phase cells (via EdU-sequencing; PMID: 36598851), which has been used to detect MiDAS loci previously (PMIDs: 32561861, 32561860). 2) Another conclusion from this section was that 'telomeres and centromeres with ssDNA lesions cluster together to facilitate post-MiDAS, likely by recruiting shared repair factors for recombination'. This is a bold claim based on a single methodology (interphase IF) which was not convincing. This claim, which would be exciting if were true, needs to be verified using a 3D genome mapping technology (HiC/MicroC).

In the second section of the results, the authors again used a candidate approach to test various factors or pathways that could play a role in post MiDAS. The common experimental set-up here was to use siRNA to deplete the target genes and then assess the frequency of EdU foci in G1 phase cells. This was then combined in some cases with IF analysis of candidate proteins. The factors tested here included a large panel of factors known to play roles in the DDR, ALT, MiDAS, APB body formation, SUMOylation, and TLS. The main conclusion from this part was that the BTR complex, FANCM-SLX4, APB body associated SUMOylation, and proteins involved in DNA translesion synthesis (TLS) and the Fanconi pathway all play a role in post-MiDAS. It is surprising that, at this point, the authors did not perform more analysis to validate one or more of the candidate proteins; rather, the authors instead searched for other factors by carrying out another screen to identify additional factors by determining the interactome of the post-MiDAS marker RPA (based on their previous work; PMID: 34158486). This Mass-Spec approach revealed that BLM, TOP3A, RMI1, RMI2, FANCM, SLX4, WRN, PML, PCNA as well as RAD18 can all be localized in the proximity of RPA32 in G1 cells, which is consistent with the results obtained from the 'candidate' approach' discussed above. One novel finding was that APC/C was enriched with RPA pull-down, and it seems that APC/CCdh1 activity can negatively regulate post-MiDAS. Taken together, the authors have identified a panel of candidates that could play a role in post-MiDAS. However, because no further validation was carried out for any of these candidate genes, no mechanistic insight has been provided in this study. In particular, it would be interesting to define which targets are important for the regulation of post-MiDAS by APC/CCDH1.

In summary, this manuscript provides a collection of screening data on the analysis of the genomic regions or proteins/pathways that could be associated in post-MiDAS, which is a very interesting topic. However, the data presented here do not come to a clear conclusion. This critical weakness is reflected on its rather clumsy and non-committal Title, and the rather vague Abstract and Discussion. In my opinion, this manuscript is not suitable for publication in Nature Communications in its current form.

Reviewer #2

(Remarks to the Author)
This study provides a well-executed analysis of replication stress–induced post-mitotic DNA synthesis (post-MiDAS), primarily at telomeres, with a more limited analysis at centromeres. Using a combination of quantitative imaging, cell-cycle–resolved assays, and complementary proteomic approaches, the authors establish telomeres and centromeres as prominent sites of post-MiDAS in G1. They identify a network of factors associated with post-MiDAS, including DNA damage tolerance pathways and the APC/C–Cdh1 complex. The finding that APC/C–Cdh1 negatively regulates excessive post-MiDAS is conceptually interesting and potentially important, particularly in the context of ALT-positive cancers.

Overall, the study is technically strong and makes a meaningful contribution to our understanding of how cells cope with inherited replication stress. The manuscript is well written and the figures are generally well crafted.

The points raised below are intended to clarify aspects of experimental design and physiological interpretation, and to further strengthen the biological conclusions rather than to challenge the validity of the data.

Major points
1. Relevance of APH + ATRi–induced post-MiDAS. A substantial fraction of the post-MiDAS analyses rely on combined treatment with aphidicolin (APH) and the ATR inhibitor AZ-20 (used at 1 µM and referred to as ATRi in the figures). I do not doubt the accuracy of the observations under these conditions. However, this experimental regime is expected to strongly suppress ATR-dependent intra-S and G2/M checkpoints. As a consequence, replication-associated DNA damage and repair intermediates that would normally induce ATR-dependent arrest may instead progress through mitosis and be inherited in G1. Thus, structures that would not normally be transmitted from S/G2 into G1 may be present in post-mitotic cells, potentially creating a non-physiological cellular context.

I appreciate that the APH + ATRi regimen robustly induces G1 post-MiDAS foci and facilitates mechanistic analysis. Nevertheless, there remains a possibility that some of the phenomena being investigated reflect post-MiDAS (or other repair processes) that occur specifically when checkpoint control is abrogated, rather than representing the typical outcome of replication stress in ALT cells under normal replication conditions.

To strengthen the relevance of the conclusions, it would be valuable to include a limited set of key experiments (e.g. from Figures 1, 2, 4, and/or 6, focusing only on key findings) performed under conditions of replication stress alone, or to examine endogenous post-MiDAS foci in the absence of APH and/or ATR inhibition.

2. Interpretation of prolonged G1-specific depletion experiments. The G1-specific depletion experiments are conceptually elegant, and the technical effort required is appreciated. However, clarification is needed regarding the biological state being interrogated under these conditions.

Based on the described protocol, cells are arrested in G1 with Palbociclib for approximately 24 hours, followed by an additional ~48 hours of Palbociclib treatment during siRNA-mediated depletion and colcemid exposure. Current models conceptualize post-MiDAS foci as lesions—often described as under-replicated DNA—carried over from the previous cell cycle. If so, the number of such foci would be expected to reflect the inherited lesion burden and not increase during G1 arrest. It is therefore unclear how foci abundance increases under prolonged G1 arrest, and whether the structures being quantified represent inherited under-replicated DNA, newly generated intermediates arising during extended G1 arrest, or dynamic remodeling of pre-existing foci that alters apparent foci number.

The prolonged arrest also raises the possibility of secondary effects, cell-cycle leakage, or low-level DNA synthesis that may complicate interpretation. Additional clarification or controls addressing replication activity and cell-cycle integrity during the arrest would help align the experimental design with the proposed model.

To address these points, I suggest the following:

A. Extend the controls in Extended Data Figure 2c–d to include
(i) FUCCI-based cell-cycle reporters, and
(ii) BrdU incorporation during the G1-specific knockdown regimen.

The FUCCI analysis would complement the GFP–H2B control by assessing potential cell-cycle abnormalities such as mitotic bypass, re-replication, or endoreduplication during the extended treatment. BrdU incorporation would directly test whether any DNA synthesis occurs during the arrest. Together, these experiments would help confirm that cells remain stably in G1 throughout the depletion period.

B. Where feasible, repeat selected key experiments (e.g. from Figures 2, 4, and/or 6 – again, focusing only on key findings) using more acute protein depletion strategies, such as degron-based approaches. This would allow targeting of proteins specifically in G1 without the need for prolonged Palbociclib arrest and would help clarify whether the observed phenotypes reflect acute loss of function in G1 rather than cumulative effects of extended arrest.

Additional minor points
3. It may be informative to repeat Extended Data Figure 7 in additional ALT cell lines to demonstrate broader relevance beyond U-2 OS. U-2 OS cells have several features that differ from other ALT-positive models (e.g. telomere length, p53 status). This may be relevant when considering therapeutic implications, but is not essential for revision.

4. The figures are generally well prepared; however, in several cases text and symbols are quite small. Enlarging labels associated with quantitative imaging data (e.g. “EdU foci counts per nucleus” and color coding in Extended Data Figure 1 and related figures) would improve readability.

5. Several dot plots are very dense (e.g. Extended Data Figure 1g–i). Use of violin plots or similar representations may improve data visualization and reader comprehension.

6. Minor typo: Line 222, Figures 3i–k are referred to; this appears to correspond to Figures 4i–k.

Reviewer #3

(Remarks to the Author)
In the present manuscript entitled “Cellular determinants of replication-stress–induced post-MIDAS at fragile genomic regions”, the authors investigate the molecular players involved in DNA synthesis occurring in G1-phase cells, referred to as post-MIDAS, following replication stress during the previous cell cycle.
Overall, I found this manuscript of interest, which reports the identification of new molecular players involved in G1 DNA synthesis, a process investigated here in cancerous ALT-positive osteosarcoma U2OS cells, which could contribute to genome stability. Notably, through the analysis of the RPA interactome in G1 cells, the authors identify APC/C–Cdh1 as a potential negative regulator of G1 DNA synthesis.

I have however several concerns:

• As the manuscript relies heavily on the identification of proteins co-localizing with EdU-positive foci in G1 cells by immunofluorescence (IF), controls demonstrating the specificity of the antibodies used by IF, such as against ubiquitin conjugates or SUMO, should be provided.

• The efficiency of siRNA-mediated depletion of several identified factors is not documented, with the exception of Cdh1. This information is missing for MCM2, RAD18, FANCD2, and Cdc20, and should be provided.

• The authors report the recruitment of TOPBP1, an ATR activator, to G1 DNA synthesis foci and propose that ATR is locally activated based on the detection of phosphorylated RPA32 by IF. This hypothesis should be further tested by experiments using a chemical inhibitor of ATR. In addition, does ATR activity prevent entry into S phase in this context, and is S-phase entry accelerated upon ATR inhibition?
• It is unclear whether APC/C–Cdh1 restrains G1 DNA synthesis through the regulation of TOPBP1 protein levels. This should be addressed by analyzing TOPBP1 expression by Western blot in a G1 cell population
• The identification of G1 cells is a critical point of the manuscript and is currently unclear. If G1 cells are defined based on QIBC analysis of DAPI and EdU signal intensities (Extended Data Figure 1), how is the EdU signal cutoff determined? Does this population correspond to Cyclin A–negative cells?
Conversely, in the Materials and Methods section, it is stated: “For the detection of EdU foci in G1-phase, cells were pre-treated with 50 ng/ml nocodazole (Sigma-Aldrich, SML1665) for 1 hour and subsequently incubated with 100 μM EdU and 50 ng/ml nocodazole for 1 hour.”
It is unclear to me how G1-phase cells can be obtained in the presence of nocodazole, which is expected to arrest cells in mitosis. This point requires clarification.

Minor comments:
• The term “total TRF2 foci intensity” at telomeres undergoing post-MIDAS (line 168) is rather confusing. Does this refer to the integrated signal intensity at each individual telomere undergoing post-MIDAS, or to the sum of intensities across all such telomeres? Please clarify.
• Figure 2d: replace “BML” with “BLM”.

Version 1:

Reviewer comments:

Reviewer #1

(Remarks to the Author)
In the revised manuscript by Vornberger et al entitled ‘Characterization of post-MiDAS at fragile genomic DNA regions in G1’, the authors have taken comments from the 3 reviewers on board and have improved the manuscript substantially. I found the revised manuscript to have more mechanistic data supporting some of the key conclusions. However, the manuscript still has some weaknesses and errors that would need to be addressed before publication.

1) In essence, the authors have identified a set of factors that promote or suppress post-MiDAS in G1 phase ALT cells. The current title of ‘Characterization of post-MiDAS at fragile genomic DNA regions in G1’ is not really suitable as the data presented only support the fact that post-MiDAS occurs at telomeres and centromeres. Most members of the replication stress community would equate ‘fragile’ with defined chromosome fragile sites, which are primarily at other loci. In addition, since there should be 15 words or fewer in the title and it should not contain technical terms and abbreviations (https://www.nature.com/ncomms/submit), I suggest revising the Title as: ‘Identification of factors regulating post-mitotic DNA

synthesis in human cells', and introducing 'Post-MiDAS in G1' in the Abstract and Introduction.

2) In the Abstract, the statement of 'Here, we provide evidence for post-MiDAS activity at centromeres and telomeres in cancer cells employing alternative lengthening of telomeres (ALT) and present data suggesting that these fragile genomic regions cluster during post-MiDAS in G1' is an overstatement. This is because, based on the data provided in the current Figure 2, only around 25% of post-MiDAS sites co-localized with both telomere and centromere foci in U-2OS cells (Figure 2d, e). It is therefore misleading to state that 'these fragile genomic regions cluster during post-MiDAS in G1'. In addition, considering that this analysis has its technical limitations, as I indicated in my first review, and that this analysis forms only a very small part of the study and does not affect the conclusions of this study, I suggest removing it from the manuscript to avoid any future misleading interpretations. This means the Abstract, main text, figure numbers etc would need to be modified accordingly. This would be helpful as this manuscript is already very long.

Relevant to this issue, I fully appreciate the technical challenges related to identifying the regions that undergo post-MiDAS. The study in this direction alone could be the focus of another manuscript. I am happy with the changes made in the text in this part in response to my earlier comment.

3) This manuscript has parts with bold and imprecise statements, which give a somewhat false impression about the data. For example, on page 12, the title of 'Canonical MiDAS factors and TLS act in post-MiDAS' is very broad and quite misleading. It is established that MiDAS is a DNA-repair based form of DNA synthesis requiring multiple processes. For example, a recent study indicates that DSB end resection factors and WRN play a role in MiDAS (https://pubmed.ncbi.nlm.nih.gov/40854910/). This means it is very difficult to pinpoint canonical MiDAS factors, and the authors have not analyzed most of them in this study anyway. Similarly, several key TLS pathway factors, such as REV1 and REV3, were not studied here either. The authors need to either include more experimental data here to match this statement, or modify the title and all the relevant text (in this section, Abstract, Discussion etc). A relevant additional note is: according to published data, BLM does not play a role in MiDAS, while WRN does. This is opposite of their roles in post-MiDAS according to this manuscript, which could be discussed in the manuscript as well.

4) The data related to APC/CCDH1's negative regulation of post-MiDAS is much more convincing in the revised manuscript. This finding is in line with previous findings of APC/CCDH1's role in tumorigenesis (for example: https://www.frontiersin.org/journals/genetics/articles/10.3389/fgene.2022.941565/full). To give the readers an idea of the significance of this data, a brief updated overview APC/CCDH1's role in tumorigenesis with relevant references should be provided in Discussion.

5) Much of the data presented in this manuscript was obtained from depletion of candidate proteins by siRNA. However, data on the efficiency of each siRNA was missing or incomplete. For example, in Figure 3a, a panel of siRNAs were used to deplete a set of proteins, but there are no WBs or qRT-PCR data to support that there was indeed efficient knockdown of those factors. Another example is: in Suppl. Figure 5, there was mRNA analysis of BLM and FANCM, to show the siRNA efficiency, but not for the other genes analyzed (SLX4, WRN, TOP3A, and POLD3). It is important that evidence for siRNA knockdown efficiency is provided in each relevant experiment.

6) Regarding data quantification, it needs to be made clear the number of replicates, and the number of cells analyzed in each condition in each replicate. Ideally, these numbers are shown in the quantification charts and then explained in the figure legend.

7) Additional errors in the text:
a. on line 50, ref 34 is not related to MiDAS, and should be removed
b. on line 52, refs 34 and 38 are not related to MiDAS, and should be removed
c. on line 229, (Figure 3q and Supplementary Figure 6e-h) should be (Figure 4q and Supplementary Figure 6e-h).

8) Regarding the Materials and Methods, the information given for antibodies used in IF and WB would be better presented in a table as well.

Reviewer #2

(Remarks to the Author)
Thank you to the authors for addressing my comments. My concerns have been addressed and I support publication.

Reviewer #3

(Remarks to the Author)

I thank the authors for the additional experiments and the revisions made to the manuscript, which have substantially reinforced the study.

**Open Access** This Peer Review File is licensed under a Creative Commons Attribution 4.0 International License, which permits use, sharing, adaptation, distribution and reproduction in any medium or format, as long as you give appropriate credit to the original author(s) and the source, provide a link to the Creative Commons license, and indicate if changes were made.
In cases where reviewers are anonymous, credit should be given to 'Anonymous Referee' and the source.
The images or other third party material in this Peer Review File are included in the article's Creative Commons license, unless indicated otherwise in a credit line to the material. If material is not included in the article's Creative Commons license and your intended use is not permitted by statutory regulation or exceeds the permitted use, you will need to obtain permission directly from the copyright holder.
To view a copy of this license, visit https://creativecommons.org/licenses/by/4.0/

We would like to thank the reviewers for their interest in our study and for having taken the time to thoroughly evaluate the manuscript and provide encouraging and constructive feedback. We were excited by their interest in our work and revised the manuscript carefully according to their helpful comments. Besides additional controls and clarifications about the experimental approach and results interpretation and revising the text, the newly identified role of APC/C$^{CDH1}$ in post-MiDAS was validated in additional cell lines and corroborated phenotypically and mechanistically. The revised manuscript contains 8 main figures, 12 supplementary figures, 3 movies, 7 supplementary tables, and a combined source data file. All new additions (13 new main and 32 new supplementary data panels) as well as all changes to the manuscript text are detailed in our point-by-point response below and highlighted in the manuscript file.

**REVIEWER COMMENTS**

Reviewer #1 (Remarks to the Author):

In the manuscript by Vornberger et al entitled 'Cellular determinants of replication stress-induced post-MiDAS', the authors aimed to identify genomic regions that undergo what they term 'post-MiDAS' and the cellular factors involved. Towards this goal, the authors performed most of their analysis in an ALT cell line (U2OS), as their previous study showed that post-MIDAS is most frequently found at telomeres of ALT-positive cancer cells (PMID: 34158486).

In the revised manuscript we validated key findings in additional ALT-positive cancer cell lines. The results corroborate and extend our findings on APC/C$^{CDH1}$ and post-MiDAS to ALT-positive SaOS-2, GM-847, and SW26 cells, and show differential effects between ALT-positive cancer cells and the ALT-negative, telomerase-positive cell lines HeLa and RPE-1 (new Figure 8g-i and Supplementary Figures 11a-i and 12g).

In the first section of the results, using IF-based methods to detect G1 phase DNA synthesis (labeled by EdU) and its co-localization with TRF1 or CREST, the authors concluded that both telomeres and centromeres are post-MiDAS regions. This analysis has two key weaknesses: 1) It is a candidate approach, and therefore other EdU foci that do not co-localize with TRF1 or CREST were overlooked. It was surprising that the authors did not use a well-established and unbiased method to detect all of the regions labelled with EdU in G1 phase cells (via EdU-sequencing; PMID: 36598851), which has been used to detect MiDAS loci previously (PMIDs: 32561861, 32561860). 2) Another conclusion from this section was that 'telomeres and centromeres with ssDNA lesions cluster together to facilitate post-MiDAS, likely by recruiting shared repair factors for recombination'. This is a bold claim based on a single methodology (interphase IF) which was not convincing. This claim, which would be exciting if were true, needs to be verified using a 3D genome mapping technology (HiC/MicroC).

We would like to clarify that we did not intend to claim in the manuscript that there are no additional genomic regions, apart from telomeres and centromeres, where post-MiDAS may occur. While the repetitive sequences at telomeres and centromeres, based on our data, are hotspots of post-MiADAS and together they explain all EdU foci that we observed in G1 cells by EdU labeling and fluorescence microscopy, it is formally possible that other regions of post-MiDAS are below the detection limit. To identify such hypothetical regions, the reviewer suggests EdU-seq, similar to MiDAS-seq, to map post-MiDAS sites in G1 cells

genome-wide. Although this may seem feasible at first glance, this is far from trivial in mammalian cancer cells and would be more prone to artefacts than MiDAS-seq: EdU-seq is already a complex experiment in asynchronous cells, which requires high cell numbers in the order of 5x10^8 cells per replicate. It is even more demanding for cells arrested in mitosis, and very few examples exist in the literature where this was done successfully. But more importantly, arresting cells in mitosis and collecting pure mitotic cell populations through mitotic shake-off, without interference from S-phase, is easier than for G1 cells: Mitotic cells are separated from S-phase through two gap phases, G1 and G2, which makes contamination from S-phase less likely; synchronization in mitosis is easier and well-established, and the mitotic shake-off greatly facilitates the separation of mitotic cells from attached interphase cells. Cultured G1 cells, on the other hand, are prone to transition into S-phase, even under synchronization conditions (e.g. thymidine- or double-thymidine-induced "G1" arrest actually stops cells at the G1/S transition) and, different from mitosis, it is technically difficult to keep mammalian cancer cells in G1 without individual cells escaping the arrest. Once replication has started, even in early S-phase, the EdU incorporation is 10-100-fold higher than in G1 (see Supplementary Figure 1b of our manuscript). The requirement of high cell numbers and thus very long FACS sorting times make it impossible to obtain G1 cells pure enough to rule out contributions from small percentages of early S-phase cells or G1-escapers to EdU-seq signals, which makes this approach highly prone to artefacts from S-phase replication (i.e measuring early S-phase origin activity rather than DNA synthesis in G1). The cell cycle resolved imaging approach, which we used in the manuscript, and which is described in more detail and clarity in the revised manuscript, deliberately avoids this. In the revised discussion, we acknowledge that post-MiDAS may occur at regions other than telomeres and centromeres, below our detection limit (page 18).

The reviewer also suggests HiC/MicroC experiments to validate the spatial proximity of two types of repetitive sequences, telomeres and centromeres, in G1 cells. This is even more demanding, faces the same challenges as EdU-seq from G1 cells, and would thus be similarly error-prone. Establishing HiC/MicroC for two types of repetitive DNA sequences in mammalian G1 cell populations, and doing so without introducing confounding artefacts, goes beyond the scope of our study. The corresponding co-localization analysis shown in our manuscript, although indeed novel and interesting, represent only one part of our study, and neither the analysis nor the interpretation was questioned by the other reviewers. However, we do acknowledge that the microscopy, which indicates clustering or telomeres and centromeres during post-MiDAS, has limitations, and we interpret these findings more carefully in the revised manuscript (revised abstract and results page 7).

In the second section of the results, the authors again used a candidate approach to test various factors or pathways that could play a role in post MiDAS. The common experimental set-up here was to use siRNA to deplete the target genes and then assess the frequency of EdU foci in G1 phase cells. This was then combined in some cases with IF analysis of candidate proteins. The factors tested here included a large panel of factors known to play roles in the DDR, ALT, MiDAS, APB body formation, SUMOylation, and TLS. The main conclusion from this part was that the BTR complex, FANCM-SLX4, APB body associated SUMOylation, and proteins involved in DNA translesion synthesis (TLS) and the Fanconi pathway all play a role in post-MiDAS. It is surprising that, at this point, the authors did not perform more analysis to validate one or more of the candidate proteins; rather, the authors instead searched for other factors by carrying out another screen to identify additional factors by determining the interactome of the post-MiDAS marker RPA (based on their previous work; PMID: 34158486). This Mass-Spec approach revealed that BLM, TOP3A, RMI1,

RMI2, FANCM, SLX4, WRN, PML, PCNA as well as RAD18 can all be localized in the proximity of RPA32 in G1 cells, which is consistent with the results obtained from the 'candidate' approach' discussed above. One novel finding was that APC/C was enriched with RPA pull-down, and it seems that APC/CCdh1 activity can negatively regulate post-MiDAS. Taken together, the authors have identified a panel of candidates that could play a role in post-MiDAS. However, because no further validation was carried out for any of these candidate genes, no mechanistic insight has been provided in this study. In particular, it would be interesting to define which targets are important for the regulation of post-MiDAS by APC/CCDH1.

The manuscript indeed combines candidate-based approaches with additional unbiased, proteome-wide approaches. The latter, involving three complementary quantitative label-free mass spectrometry experiments, identified APC/C$^{CDH1}$ as novel candidate regulator of post-MiDAS. A functional validation screen, using three independent siRNAs against the proteins identified in the three proteomics screens, confirmed a role of APC/C in post-MiDAS (Supplementary Figure 10g). Based on the comments from this reviewer and from the other reviewers, we extended the validation and molecular characterization of APC/C$^{CDH1}$ as post-MiDAS regulator. We now show in four different cell lines by Western blot that depletion of CDH1 increases TOPBP1 levels (new Figure 7d and new Supplementary Figures 11a, d, g). Consistent with G1-specific functions of CDH1, the effect on TOPBP1 protein levels was more pronounced when cells were arrested in G1 by Palbociclib (Figure 7d). In agreement with the U-2 OS results, TOPBP1 foci in G1 were enhanced upon CDH1 knockdown in SaOS-2, GM-847 and SW26 cells (new Supplementary Figures 11b, e, h), and also post-MiDAS activity was enhanced in these ALT-positive cancer cell lines after CDH1 loss (new Supplementary Figures 11c, f, i). The effect of CDH1 depletion on TOPBP1 and post-MiDAS was observed with two independent siRNAs against CDH1 (Figure 7c and Supplementary Figures 10e, f, n, o, p), and the knockdown of CDH1 did not result in increased TOPBP1 mRNA levels (new Supplementary Figures 10k, l), consistent with post-translational regulation of TOPBP1 by APC/C$^{CDH1}$. We also observed an interaction by co-immunoprecipitation between endogenous APC/C$^{CDH1}$ and endogenous TOPBP1, despite such enzyme-substrate interactions often being very transient in cells. Collectively, these experimental data suggest that TOPBP1 is a target of APC/C$^{CDH1}$ in G1, consistent with prior *in silico* prediction of APC/C targets (PMID: 33306668). In our initial submission, we provided evidence that TOPBP1 is required for RPA32 phosphorylation at S33 at post-MiDAS sites (now Figure 8a-d) and that CDH1 depletion has the opposite effect (now Figure 8e, f). These results suggest that TOPBP1 promotes local ATR activity at post-MiDAS sites. In the revised manuscript, we additionally show that ATR inhibition, using two different ATR inhibitors, suppresses RPA phosphorylation at S33 and post-MiDAS activity (new Supplementary Figures 12c-e). Thus, APC/C$^{CDH1}$ controls TOPBP1 levels and constrains local ATR activation and post-MiDAS activity. In line with CDH1-constrained post-MiDAS activity, we show in our revised manuscript that CDH1 depletion increases APB formation in two ALT-positive cell lines (U-2 OS and SaOS-2) but not in ALT-negative cells (HeLa and RPE-1) (new Figure 8g). Similar to U-2 OS cells, we found SaOS-2 to be sensitive to CDH1 depletion in colony formation assays (Figure 8h, i). Together, these additional results corroborate the role of APC/C$^{CDH1}$ and, in our view, significantly strengthen the manuscript.

In summary, this manuscript provides a collection of screening data on the analysis of the genomic regions or proteins/pathways that could be associated in post-MiDAS, which is a very interesting topic. However, the data presented here do not come to a clear conclusion. This critical weakness is reflected on its rather clumsy and non-committal Title, and the

rather vague Abstract and Discussion. In my opinion, this manuscript is not suitable for publication in Nature Communications in its current form.

We appreciate that the reviewer considers post-MiDAS a very interesting topic. Apart from our previous study, very little is known about this process. Our current study was thus aimed at clarifying whether known ALT and MiDAS factors are involved in post-MiDAS in G1, and at identifying novel factors that may be specific for post-MiDAS. APC/C together with its G1-specific co-activator CDH1 was identified as such a factor, supported by the fact that CDC20, the co-activator of APC/C in mitosis, does not affect post-MiDAS (Figure 7b and Supplementary Figure 10d). This is an important finding, in our opinion, because it suggests that although certain mechanisms are shared between post-MiDAS in G1, MiDAS in mitosis, and ALT in S/G2, distinct and cell cycle-specific regulation exists as well. We hope that with the new additions and the revised text (including revised title and abstract), the key findings and conclusions are conveyed more clearly. The revised manuscript also includes several additional controls and validation experiments, which corroborate the experimental approach and support our interpretations (please see our response to reviewers #2 and #3 below).

Reviewer #2 (Remarks to the Author):

This study provides a well-executed analysis of replication stress–induced post-mitotic DNA synthesis (post-MiDAS), primarily at telomeres, with a more limited analysis at centromeres. Using a combination of quantitative imaging, cell-cycle–resolved assays, and complementary proteomic approaches, the authors establish telomeres and centromeres as prominent sites of post-MiDAS in G1. They identify a network of factors associated with post-MiDAS, including DNA damage tolerance pathways and the APC/C–Cdh1 complex. The finding that APC/C–Cdh1 negatively regulates excessive post-MiDAS is conceptually interesting and potentially important, particularly in the context of ALT-positive cancers.

Overall, the study is technically strong and makes a meaningful contribution to our understanding of how cells cope with inherited replication stress. The manuscript is well written and the figures are generally well crafted.

We were thrilled to read that the reviewer considered our study well-executed, technically strong and making a meaningful contribution to the field. This was very encouraging, and the manuscript was revised to enhance clarity and provide previously missing explanations and additional controls, as suggested by the reviewer.

The points raised below are intended to clarify aspects of experimental design and physiological interpretation, and to further strengthen the biological conclusions rather than to challenge the validity of the data.

Major points

1. Relevance of APH + ATRi–induced post-MiDAS. A substantial fraction of the post-MiDAS analyses rely on combined treatment with aphidicolin (APH) and the ATR inhibitor AZ-20 (used at 1 µM and referred to as ATRi in the figures). I do not doubt the accuracy of the observations under these conditions. However, this experimental regime is expected to strongly suppress ATR-dependent intra-S and G2/M checkpoints. As a consequence, replication-associated DNA damage and repair intermediates that would normally induce

ATR-dependent arrest may instead progress through mitosis and be inherited in G1. Thus, structures that would not normally be transmitted from S/G2 into G1 may be present in post-mitotic cells, potentially creating a non-physiological cellular context.

I appreciate that the APH + ATRi regimen robustly induces G1 post-MiDAS foci and facilitates mechanistic analysis. Nevertheless, there remains a possibility that some of the phenomena being investigated reflect post-MiDAS (or other repair processes) that occur specifically when checkpoint control is abrogated, rather than representing the typical outcome of replication stress in ALT cells under normal replication conditions.

To strengthen the relevance of the conclusions, it would be valuable to include a limited set of key experiments (e.g. from Figures 1, 2, 4, and/or 6, focusing only on key findings) performed under conditions of replication stress alone, or to examine endogenous post-MiDAS foci in the absence of APH and/or ATR inhibition.

The reviewer is absolutely correct that it is important to analyze post-MiDAS both in the presence and absence of exogenous replication stress (e.g. upon APH and/or ATRi). We may have missed to point out in the initial submission that in almost all of the experiments both conditions – unchallenged cells without exogenous replication stress and cells exposed to APH+ATRi – are shown. Post-MiDAS foci can be seen in unperturbed, asynchronously growing U-2 OS cells, and the treatment with APH+ATRi enhances this phenotype. Please see, for example, revised Figure 1a-d, e-f, g, h, i, j, l. The same applies to the other figures and IF images, where post-MiDAS-associated events are shown (e.g. revised Figures 2a, 2d, 3d, 4c, 4d, 4k, 4l, 5b, 5e, 5i, 7h, 8a, and related quantifications).

We would like to point out that, unless a replication stress treatment (e.g. APH or APH+ATRi) is specifically indicated in the figure or figure legend, no treatment was performed. For instance, no APH+ATRi treatment was performed for the siRNA screens (revised Figure 3a-c and S10g) and for the proteomics experiments and the APC/$C^{CDH1}$ analyses (Figure 6, Figure 7a-g, Figure 8d-I, Figures S8-S12).

In the revised manuscript text, at the beginning of the results section (page 6), we explain the experimental approach in more detail, and whenever applicable we clarified if untreated cells were analyzed or if they were compared to treated cells.

2. Interpretation of prolonged G1-specific depletion experiments. The G1-specific depletion experiments are conceptually elegant, and the technical effort required is appreciated. However, clarification is needed regarding the biological state being interrogated under these conditions.

Based on the described protocol, cells are arrested in G1 with Palbociclib for approximately 24 hours, followed by an additional ~48 hours of Palbociclib treatment during siRNA-mediated depletion and colcemid exposure. Current models conceptualize post-MiDAS foci as lesions—often described as under-replicated DNA—carried over from the previous cell cycle. If so, the number of such foci would be expected to reflect the inherited lesion burden and not increase during G1 arrest. It is therefore unclear how foci abundance increases under prolonged G1 arrest, and whether the structures being quantified represent inherited under-replicated DNA, newly generated intermediates arising during extended G1 arrest, or dynamic remodeling of pre-existing foci that alters apparent foci number.

The prolonged arrest also raises the possibility of secondary effects, cell-cycle leakage, or low-level DNA synthesis that may complicate interpretation. Additional clarification or controls addressing replication activity and cell-cycle integrity during the arrest would help align the experimental design with the proposed model.

To address these points, I suggest the following:

A. Extend the controls in Extended Data Figure 2c–d to include
(i) FUCCI-based cell-cycle reporters, and
(ii) BrdU incorporation during the G1-specific knockdown regimen.

The FUCCI analysis would complement the GFP–H2B control by assessing potential cell-cycle abnormalities such as mitotic bypass, re-replication, or endoreduplication during the extended treatment. BrdU incorporation would directly test whether any DNA synthesis occurs during the arrest. Together, these experiments would help confirm that cells remain stably in G1 throughout the depletion period.

We appreciate these comments and the suggestions from the reviewer. To address these points, we (a) revised the manuscript text to better explain the experimental approach (page 6 and 8), (b) we revised Figure 1 and Supplementary Figure 1 to include additional graphs explaining the G1-specific analysis in untreated and replication stress conditions, (c) we included a new Supplementary Figure 2, in which G1 cell identification based on DAPI and EdU is correlated with FUCCI cell cycle reports and additionally with Cyclin A to discriminate G1 (Cylin A low) from S/G2 (Cyclin A high), (d) we included time-lapse movies of GFP-H2B cells (new Supplementary Movies 1-3), which show that upon Palbociclib and colcemid treatment no cells exit mitosis and enter G1, (e) we performed the suggested BrdU incorporation experiment (new Supplementary Figure 4h-j), which shows that almost no cells (less than 3%) become BrdU-positive during the arrest, and (f) we performed a 72 hour G1 arrest with Palbociclib followed by an 8 hour release, which shows that the arrest is reversible and that even after 72 hours in Palbociclib cells readily re-enter S-phase upon release (new Supplementary Figure 4f, g). Please also note that the microscopy-based readouts by QIBC are deliberately focused on G1 cells with a 2N DNA content (Supplementary Figure 4a), excluding the possibility that events such as mitotic bypass, re-replication, or endoreduplication would skew the analysis.

B. Where feasible, repeat selected key experiments (e.g. from Figures 2, 4, and/or 6 – again, focusing only on key findings) using more acute protein depletion strategies, such as degron-based approaches. This would allow targeting of proteins specifically in G1 without the need for prolonged Palbociclib arrest and would help clarify whether the observed phenotypes reflect acute loss of function in G1 rather than cumulative effects of extended arrest.

We agree that acute protein depletion using degrons would be an elegant strategy to confirm knockdown results. Unfortunately, examples of homozygous endogenous protein tagging with degrons in ALT-positive cell lines are rare. ALT-positive cancer cells are often hyperploid, complicating efficient degron-mediated protein depletion, and it was not possible during the period of the revision to generate cell lines for acute, degron-based depletion of the identified post-MiDAS factors. However, and inspired by the reviewer's comment, we show in the revised manuscript that acute inhibition of BLM impairs EdU incorporation at acutely damaged ALT telomeres (new Supplementary Figure 5f), consistent with the siRNA-mediated knockdown results.

Additional minor points

1. It may be informative to repeat Extended Data Figure 7 in additional ALT cell lines to demonstrate broader relevance beyond U-2 OS. U-2 OS cells have several features that differ from other ALT-positive models (e.g. telomere length, p53 status). This may be relevant when considering therapeutic implications, but is not essential for revision.

We have extended these analyses to ALT-positive SaOS-2 cells and show in the revised manuscript that CDH1 depletion similarly affects U-2 OS and SaOS-2 cells, both at the level of colony formation (Figure 8h, i) and APB formation in G1 (new Figure 8g), despite their differences in p53 status. Additionally, we show that SaOS-2 cells as well as ALT-positive GM-847 and SW26 cells have elevated TOPBP1 levels and increased post-MiDAS activity upon CDH1 depletion (new Supplementary Figure 11a-i), similar to the U-2 OS results.

2. The figures are generally well prepared; however, in several cases text and symbols are quite small. Enlarging labels associated with quantitative imaging data (e.g. "EdU foci counts per nucleus" and color coding in Extended Data Figure 1 and related figures) would improve readability.

Thank you. We increased the text size and symbols to improve readability.

3. Several dot plots are very dense (e.g. Extended Data Figure 1g–i). Use of violin plots or similar representations may improve data visualization and reader comprehension.

We exchanged dense dot plots to violin plots as suggested.

4. Minor typo: Line 222, Figures 3i–k are referred to; this appears to correspond to Figures 4i–k.

Thank you very much, has been corrected.

Reviewer #3 (Remarks to the Author):

In the present manuscript entitled "Cellular determinants of replication-stress–induced post-MIDAS at fragile genomic regions", the authors investigate the molecular players involved in DNA synthesis occurring in G1-phase cells, referred to as post-MIDAS, following replication stress during the previous cell cycle.
Overall, I found this manuscript of interest, which reports the identification of new molecular players involved in G1 DNA synthesis, a process investigated here in cancerous ALT-positive osteosarcoma U2OS cells, which could contribute to genome stability. Notably, through the analysis of the RPA interactome in G1 cells, the authors identify APC/C–Cdh1 as a potential negative regulator of G1 DNA synthesis.

We appreciate the interest in the topic and the constructive comments on our manuscript.

I have however several concerns:

As the manuscript relies heavily on the identification of proteins co-localizing with EdU-positive foci in G1 cells by immunofluorescence (IF), controls demonstrating the specificity

of the antibodies used by IF, such as against ubiquitin conjugates or SUMO, should be provided.

The requested antibody specificity controls for IF have been included: SUMO1 in Supplementary Figure 6e, f; SUMO2/3 in Supplementary Figure 6g, h; ubiquitin in Supplementary Figure 7e, f. Additional antibody specificity controls for IF can be found in Supplementary Figures 4e, 6d, 7b, and 12a.

The efficiency of siRNA-mediated depletion of several identified factors is not documented, with the exception of Cdh1. This information is missing for MCM2, RAD18, FANCD2, and Cdc20, and should be provided.

The requested knockdown controls have been included: MCM2 in Supplementary Figure 7c; RAD18 in Supplementary Figure 7h; FANCD2 in Supplementary Figure 7k; CDC20 in Supplementary Figure 10d. Additional knockdown controls by qPCR can be found in Supplementary Figures 5d, e, and 12b.

The authors report the recruitment of TOPBP1, an ATR activator, to G1 DNA synthesis foci and propose that ATR is locally activated based on the detection of phosphorylated RPA32 by IF. This hypothesis should be further tested by experiments using a chemical inhibitor of ATR. In addition, does ATR activity prevent entry into S phase in this context, and is S-phase entry accelerated upon ATR inhibition?

We assessed ATR activity in the context of post-MiDAS using two chemical inhibitors (AZ-20 and VE-821). Upon induced post-MiDAS following FANCM depletion, ATR inhibition clearly suppressed pRPA S33 foci in G1 and reduced post-MiDAS activity (new Supplementary Figure 12c-e). Inhibition of ATR also induced accelerated S-phase entry, as measured upon release from Palbociclib-induced G1 arrest. We provide these results for the reviewers (Figure R1).

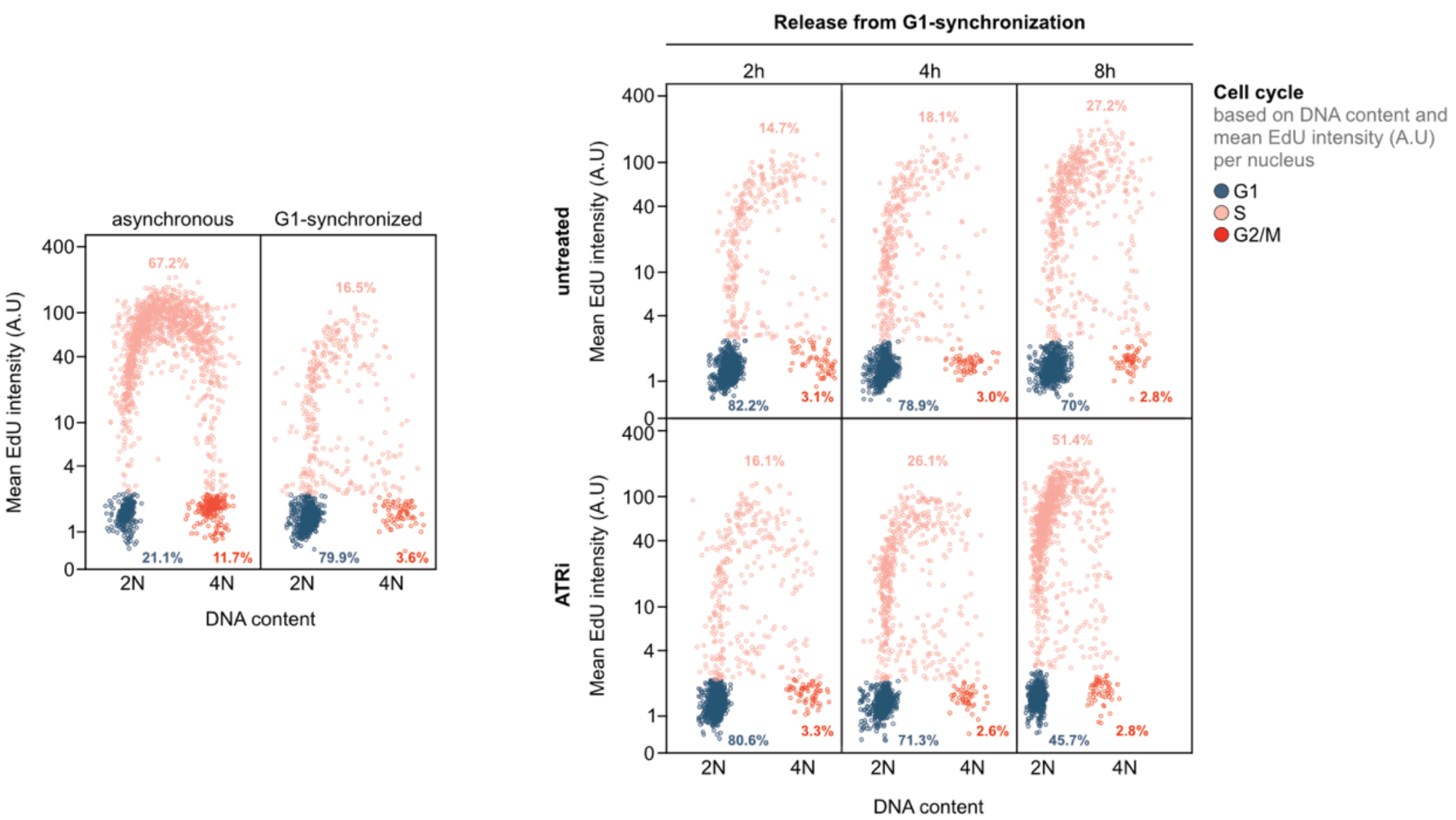


**Figure R1: ATR inhibition accelerates G1/S transition.** U-2 OS cells were treated with 1 µM Palbociclib for 24 h and then released into fresh medium without or with ATRi (1 µM AZ-20) for 2, 4, or 8 h. S-phase entry was analyzed by QIBC. The percentages of cells in G1, S and G2/M are indicated. Note that the G1 population decreases faster and that the S-phase population increases faster in the presence of ATRi.

It is unclear whether APC/C–Cdh1 restrains G1 DNA synthesis through the regulation of TOPBP1 protein levels. This should be addressed by analyzing TOPBP1 expression by Western blot in a G1 cell population

As suggested, we analyzed TOPBP1 levels by Western blot, both in asynchronous cells and in G1 cell populations. Consistent with the IF data, TOPBP1 levels were increased upon CDH1 depletion, and this effect was more pronounced in G1 cells (new Figure 7d). TOPBP1 mRNA levels, however, were not increased upon CDH1 depletion (Supplementary Figure 10k, l), in agreement with post-translational regulation of TOPBP1 by APC/$C^{CDH1}$.

The identification of G1 cells is a critical point of the manuscript and is currently unclear. If G1 cells are defined based on QIBC analysis of DAPI and EdU signal intensities (Extended Data Figure 1), how is the EdU signal cutoff determined? Does this population correspond to Cyclin A–negative cells?

We explain the experimental approach and G1 identification in more detail in the revised manuscript text (page 6) and provide additional data graphs to illustrate the G1 identification (Figure 1a-d, Supplementary Figure 1a-d, Supplementary Figure 2a, b, Supplementary Figure 4a). The color-code in these figures illustrates how the cutoff was set. Because EdU incorporation increases rapidly upon S-phase entry, the G1 population can be easily distinguished from S-phase cells (see Supplementary Figure 2a, for example). The G1 cells identified in that manner correspond to G1 cells identified by FUCCI cell cycle markers (RFP-CDT1 and GFP-Geminin), which in turn correspond to Cyclin A-negative cells with a 2N (G1) DNA content (Supplementary Figure 2a, b).

Conversely, in the Materials and Methods section, it is stated: “For the detection of EdU foci in G1-phase, cells were pre-treated with 50 ng/ml nocodazole (Sigma-Aldrich, SML1665) for 1 hour and subsequently incubated with 100 µM EdU and 50 ng/ml nocodazole for 1 hour.” It is unclear to me how G1-phase cells can be obtained in the presence of nocodazole, which is expected to arrest cells in mitosis. This point requires clarification.

This is now explained better in the revised manuscript text (page 6). The EdU pulse labeling was performed in the presence of nocodazole to avoid mitotic cells from entering G1 during the period of the 1 h EdU pulse. Hence, any EdU incorporation comes from G1 cells, and not from mitotic cells that entered G1 during the EdU pulse.

The nocodazole was added slightly before the EdU pulse was started to ensure that exit from mitosis is blocked before cells can start to incorporate EdU. The short (2 h) nocodazole treatment does not arrest/synchronize the asynchronous cell population and does not lead to a significant depletion of the G1 population (different from longer nocodazole treatments). It does make the experiment cleaner, in our opinion, because EdU incorporation during MiDAS can be excluded as a confounding factor in the analyzed G1 population.

Minor comments:

The term “total TRF2 foci intensity” at telomeres undergoing post-MIDAS (line 168) is rather confusing. Does this refer to the integrated signal intensity at each individual telomere undergoing post-MIDAS, or to the sum of intensities across all such telomeres? Please clarify.

It refers to the former and has been revised to "Total TRF2 intensity (A.U.) at TRF2 foci in G1" in the figure axis labelling (Figure 4a, b) and in the text.

Figure 2d: replace "BML" with "BLM".

Has been corrected, thank you.

We thank the reviewers for their interest and for having taken the time to evaluate the revised manuscript. We were thrilled to read that reviewer #2 and reviewer #3 were satisfied with the revision and support publication of our work in *Nature Communications.* This was very encouraging and we appreciate their constructive comments on our initial submission. We were equally exited that reviewer #1 also concluded that the manuscript was improved substantially following their helpful advice, and we have done our best to address the remaining remarks.

Reviewer #1 (Remarks to the Author):

In the revised manuscript by Vornberger et al entitled 'Characterization of post-MiDAS at fragile genomic DNA regions in G1', the authors have taken comments from the 3 reviewers on board and have improved the manuscript substantially. I found the revised manuscript to have more mechanistic data supporting some of the key conclusions. However, the manuscript still has some weaknesses and errors that would need to be addressed before publication.

1) In essence, the authors have identified a set of factors that promote or suppress post-MiDAS in G1 phase ALT cells. The current title of 'Characterization of post-MiDAS at fragile genomic DNA regions in G1' is not really suitable as the data presented only support the fact that post-MiDAS occurs at telomeres and centromeres. Most members of the replication stress community would equate 'fragile' with defined chromosome fragile sites, which are primarily at other loci. In addition, since there should be 15 words or fewer in the title and it should not contain technical terms and abbreviations (https://www.nature.com/ncomms/submit), I suggest revising the Title as: 'Identification of factors regulating post-mitotic DNA synthesis in human cells', and introducing 'Post-MiDAS in G1' in the Abstract and Introduction.

Thank you for this suggestion. We agree that the title should be concise and should not contain technical terms or uncommon abbreviations. Based on and in line with the reviewer's comments we have changed the title to: "Characterization of post-mitotic DNA synthesis and its regulation in human cells" and introduce post-MiDAS in G1 in the abstract and introduction.

2) In the Abstract, the statement of 'Here, we provide evidence for post-MiDAS activity at centromeres and telomeres in cancer cells employing alternative lengthening of telomeres (ALT) and present data suggesting that these fragile genomic regions cluster during post-MiDAS in G1' is an overstatement. This is because, based on the data provided in the current Figure 2, only around 25% of post-MiDAS sites co-localized with both telomere and centromere foci in U-2OS cells (Figure 2d, e). It is therefore misleading to state that 'these fragile genomic regions cluster during post-MiDAS in G1'. In addition, considering that this analysis has its technical limitations, as I indicated in my first review, and that this analysis forms only a very small part of the study and does not affect the conclusions of this study, I suggest removing it from the manuscript to avoid any future misleading interpretations. This means the Abstract, main text, figure numbers etc would need to be modified accordingly. This would be helpful as this manuscript is already very long.

The co-localization analyses suggest an interesting, novel and potentially quite relevant link between repair processes at telomeres and centromeres. Moreover, a recent article that was published online ahead of print in *Nature* during the revision of our manuscript strongly supports

our conclusions and demonstrates that DNA sequences are exchanged between telomeres and centromeres during ALT recombination (Bhargava et al. *Nature*. 2026 Jun 3. doi: 10.1038/s41586-026-10598-1. Online ahead of print: Centromeric footprints preserve telomere integrity in ALT cancers). Publishing our co-localization results, which are complementary to the data provided by Bhargava et al., provides independent support for the notion that telomeres and centromeres recombine in ALT cells. We had hypothesized already in 2021 that centromeres, like telomeres, might undergo post-MiDAS, and found in 2022 that "telomeres and centromeres undergoing post-MiDAS sometimes cluster together" (PhD thesis committee meeting report from October 24th, 2022). We are convinced that it is beneficial and of interest for the field to see independently obtained evidence for this unexpected and largely unexplored mechanism. In response to the reviewer's concern, we specified in the revised abstract that we "…present data suggesting that *a subset of* these fragile genomic regions *can* cluster during post-MiDAS in G1".

Relevant to this issue, I fully appreciate the technical challenges related to identifying the regions that undergo post-MiDAS. The study in this direction alone could be the focus of another manuscript. I am happy with the changes made in the text in this part in response to my earlier comment.

3) This manuscript has parts with bold and imprecise statements, which give a somewhat false impression about the data. For example, on page 12, the title of 'Canonical MiDAS factors and TLS act in post-MiDAS' is very broad and quite misleading. It is established that MiDAS is a DNA-repair based form of DNA synthesis requiring multiple processes. For example, a recent study indicates that DSB end resection factors and WRN play a role in MiDAS (https://pubmed.ncbi.nlm.nih.gov/40854910/). This means it is very difficult to pinpoint canonical MiDAS factors, and the authors have not analyzed most of them in this study anyway. Similarly, several key TLS pathway factors, such as REV1 and REV3, were not studied here either. The authors need to either include more experimental data here to match this statement, or modify the title and all the relevant text (in this section, Abstract, Discussion etc). A relevant additional note is: according to published data, BLM does not play a role in MiDAS, while WRN does. This is opposite of their roles in post-MiDAS according to this manuscript, which could be discussed in the manuscript as well.

We omitted the term "canonical" in the context of MiDAS, made the suggested text changes related to TLS, and briefly discuss distinct roles of BLM and WRN during MiDAS and post-MiDAS. All changes are highlighted in the revised manuscript text (abstract, pages 12-13 and 19-20) and they include the above-mentioned reference on the role of WRN in MiDAS.

4) The data related to APC/CCDH1's negative regulation of post-MiDAS is much more convincing in the revised manuscript. This finding is in line with previous findings of APC/CCDH1's role in tumorigenesis (for example: https://www.frontiersin.org/journals/genetics/articles/10.3389/fgene.2022.941565/full). To give the readers an idea of the significance of this data, a brief updated overview APC/CCDH1's role in tumorigenesis with relevant references should be provided in Discussion.

Thank you for this suggestion. We included this aspect in the discussion and refer the interested reader to two comprehensive reviews, including the one that the reviewer mentioned (page 20).

5) Much of the data presented in this manuscript was obtained from depletion of candidate proteins by siRNA. However, data on the efficiency of each siRNA was missing or incomplete. For example, in Figure 3a, a panel of siRNAs were used to deplete a set of proteins, but there are no WBs or qRT-PCR data to support that there was indeed efficient knockdown of those factors. Another example is: in Suppl. Figure 5, there was mRNA analysis of BLM and FANCM, to show the siRNA efficiency, but not for the other genes analyzed (SLX4, WRN, TOP3A, and POLD3). It is important that evidence for siRNA knockdown efficiency is provided in each relevant experiment.

Figure 3a shows the results of a targeted siRNA screen (see Table S6 for a complete list of the 85 individual siRNAs that were used). It is typically not feasible to control knockdown efficiencies in screening settings. Indeed, most if not all RNAi and CRISPR screens in the scientific literature do not verify the efficacy of each individual siRNA or sgRNA simply because this is not possible, given the cost and the logistical and technical limitations of doing so. Moreover, low levels of residual expression can be sufficient for protein functions in specific processes. Different from the positive ‘hits’ that the screen revealed, negative results (i.e. no observed phenotype in a particular readout) should be interpreted cautiously, in our opinion, and are often not fully conclusive. This arguably applies to RNAi, CRISPR and drug screens as well as to various kinds of proteomics experiments (absence of evidence is not evidence of absence). For the individual knockdowns of SLX4, WRN, TOP3A, and POLD3 shown in Supplementary Figure 5a, we now provide qRT-PCR results confirming the siRNA-mediated knockdowns (new Supplementary Figure 5b-h; the new qRT-PCR primers were included in Supplementary Table 8).

6) Regarding data quantification, it needs to be made clear the number of replicates, and the number of cells analyzed in each condition in each replicate. Ideally, these numbers are shown in the quantification charts and then explained in the figure legend.

Information about cell numbers and replicates were included in the reporting summary, the source data file, and in the figure legends wherever applicable. The source data file comprises close to 150 individual sheets, corresponding to individual results panels shown in the main and supplementary figures, and we would be happy to work with the editorial office to ensure that the manuscript complies with the latest journal reporting and formatting guidelines.

7) Additional errors in the text:
a. on line 50, ref 34 is not related to MiDAS, and should be removed

Reference 34 “How unfinished business from S-phase affects mitosis and beyond” from Ian Hickson discusses the processes involved in what is now known as MiDAS, and the article is related to how “cells attempt to complete DNA replication at these [under-replicated] sites in early mitosis” (lines 49-50). We would thus prefer to keep this reference.

b. on line 52, refs 34 and 38 are not related to MiDAS, and should be removed

References were removed as suggested.

c. on line 229, (Figure 3q and Supplementary Figure 6e-h) should be (Figure 4q and Supplementary Figure 6e-h).

Was corrected, thank you for spotting this.

8) Regarding the Materials and Methods, the information given for antibodies used in IF and WB would be better presented in a table as well.

As suggested, we prepared an additional supplementary table for all antibodies that were used in IF and WB (new Supplementary Table 7).

Reviewer #2 (Remarks to the Author):

Thank you to the authors for addressing my comments. My concerns have been addressed and I support publication.

We are grateful to the reviewer for constructive comments on our manuscript and for supporting its publication.

Reviewer #3 (Remarks to the Author):

I thank the authors for the additional experiments and the revisions made to the manuscript, which have substantially reinforced the study.

We are grateful to the reviewer for constructive comments on our manuscript and for supporting its publication.
