# Supplementary material for: Characterization of post-mitotic DNA synthesis and its regulation in human cells
Source: Nat Commun. 2026 Sep 28;17:11186. doi: 10.1038/s41467-026-78035-5 (PMC13713988; doi:10.1038/s41467-026-78035-5)
Supplement: Supplementary file 3 [file 41467_2026_78035_MOESM1_ESM.pdf]

Supplementary Information

Characterization of post-mitotic DNA synthesis and its regulation in human cells

Julia Vornberger[1,2], Ralph Imhof[1,#], Virginia Pasti[1,2,#], Beatrice Bianchi[1,2], Mingxuan Wei[1,2], Jeannette Alisha Straw[1], Sandhya Manohar[1], Matthias Altmeyer[1,*]

[1] Department of Molecular Mechanisms of Disease, University of Zurich (UZH), Zurich, Switzerland

[2] Molecular Life Sciences PhD Program, Life Science Zurich Graduate School, Zurich, Switzerland

[#] These authors contributed equally

* Correspondence: matthias.altmeyer@uzh.ch

Content:

Supplementary Figures 1-12

Supplementary Tables 1-3

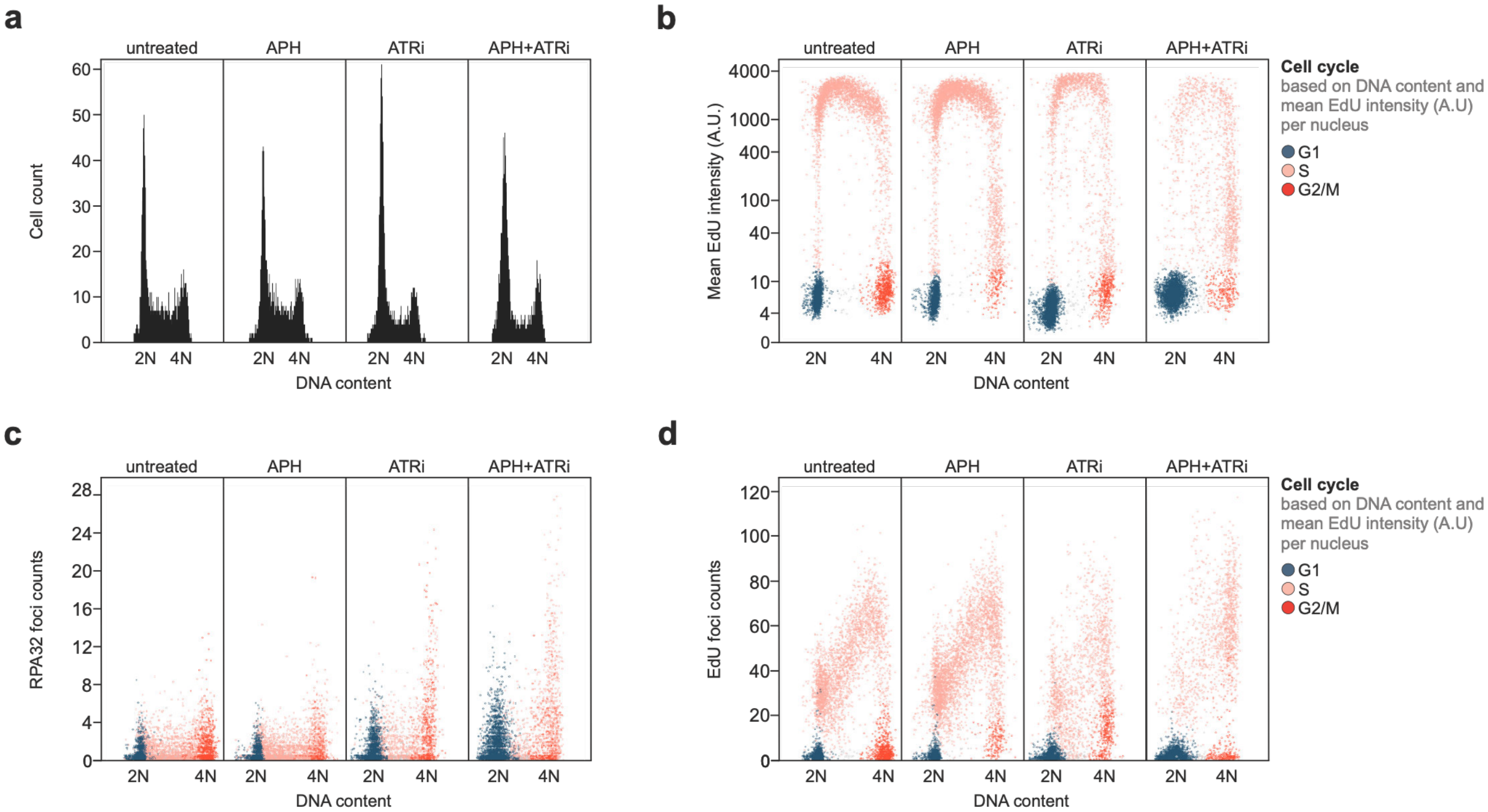

Supplementary Figure 1: QIBC-based cell cycle-resolved analysis of RPA and EdU foci formation following replication stress. a High-content microscopy-derived cell cycle profiles based on DNA content (total DAPI intensity (A.U.)) per U-2 OS nucleus. Cells were untreated or treated with APH (0.2 µM), ATRi (1 µM), or both for 24 hours (n = 5000 cells per condition). **b** Cell cycle profiles based on total DAPI intensity (A.U.) and EdU incorporation (mean EdU intensity (A.U.)) per U-2 OS nucleus as in (a). The color code indicates cell cycle phase defined by DNA content and mean EdU intensity (n = 5000 cells per condition). **c, d** Cell cycle-resolved RPA32 (c) and EdU (d) foci formation based on total DAPI intensity (A.U.) per U-2 OS nucleus as in (a). The color code indicates cell cycle phase as defined in (b) (n = 5000 cells per condition). The data of the untreated and APH+ATRi conditions correspond to those shown in Figure 1a–f. A.U., arbitrary units. Source data are provided as a Source Data file.

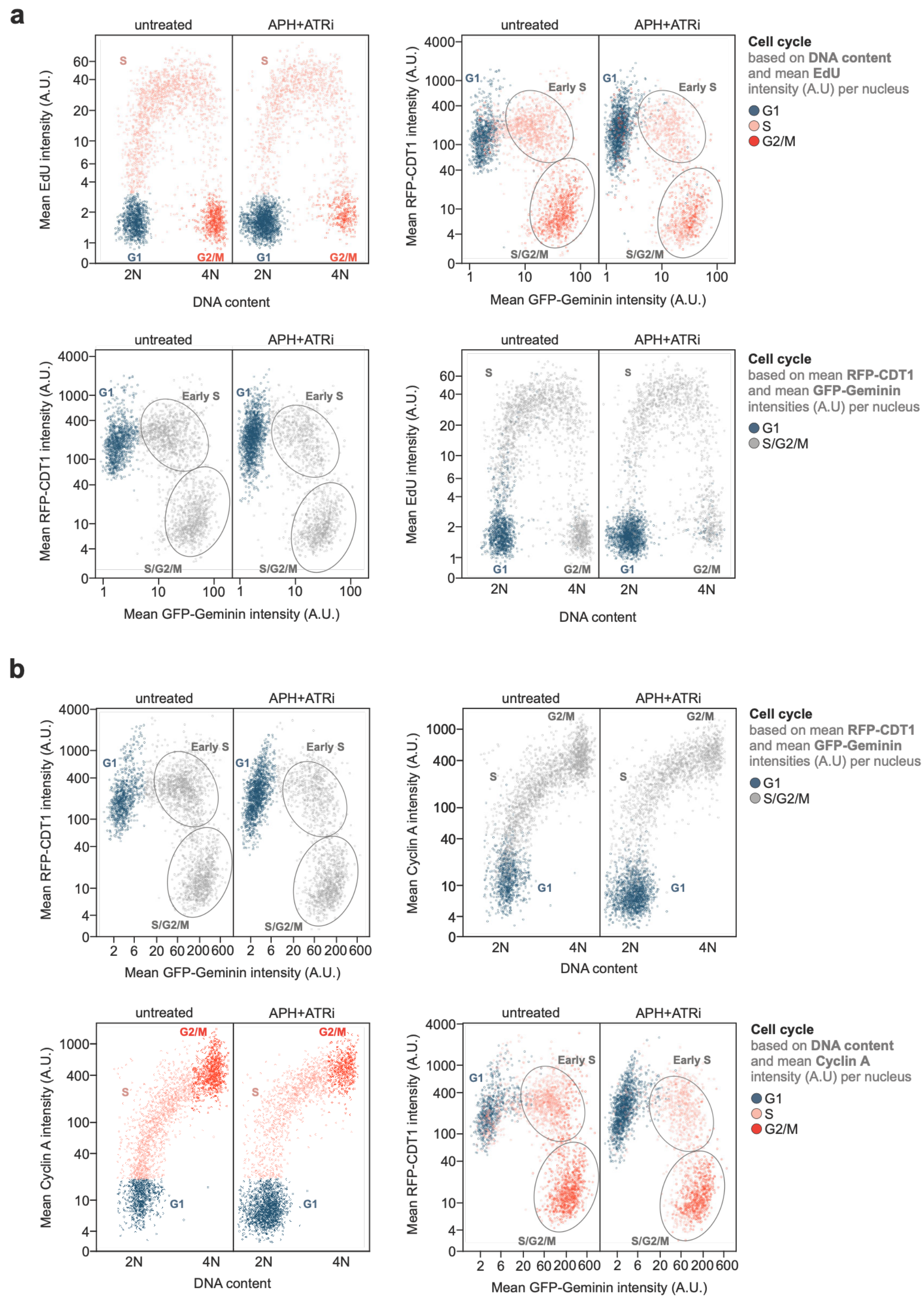

Supplementary Figure 2: G1-phase identification by DNA content, EdU incorporation, FUCCI markers, and Cyclin A. a High-content microscopy-derived cell cycle profiles based on DNA content (total DAPI intensity (A.U.)) and EdU incorporation (mean EdU intensity (A.U.)) or GFP-Geminin (mean GFP-Geminin intensity (A.U.)) and RFP-CDT1 expression (mean RFP-CDT1 intensity (A.U.)) per U-2 OS FUCCI nucleus. Cells were untreated or treated with APH (0.2 µM) and ATRi (1 µM) for 24 hours. Color code and gating indicate the cell cycle phase defined by DNA content and mean EdU intensity or by FUCCI signals (n = 2800 cells per condition). **b** Cell cycle profiles based on GFP-Geminin (mean GFP-Geminin intensity (A.U.)) and RFP-CDT1 expression (mean RFP-CDT1 intensity (A.U.)) or DNA content (total DAPI intensity (A.U.)) and Cyclin A expression (mean Cyclin A intensity (A.U.)) per U-2 OS FUCCI nucleus as in (a). Color code and gating indicate the cell cycle phase defined by FUCCI signals or by DNA content and mean Cyclin A intensity (A.U.) per nucleus (n = 2800 cells per condition). A.U., arbitrary units. Source data are provided as a Source Data file.

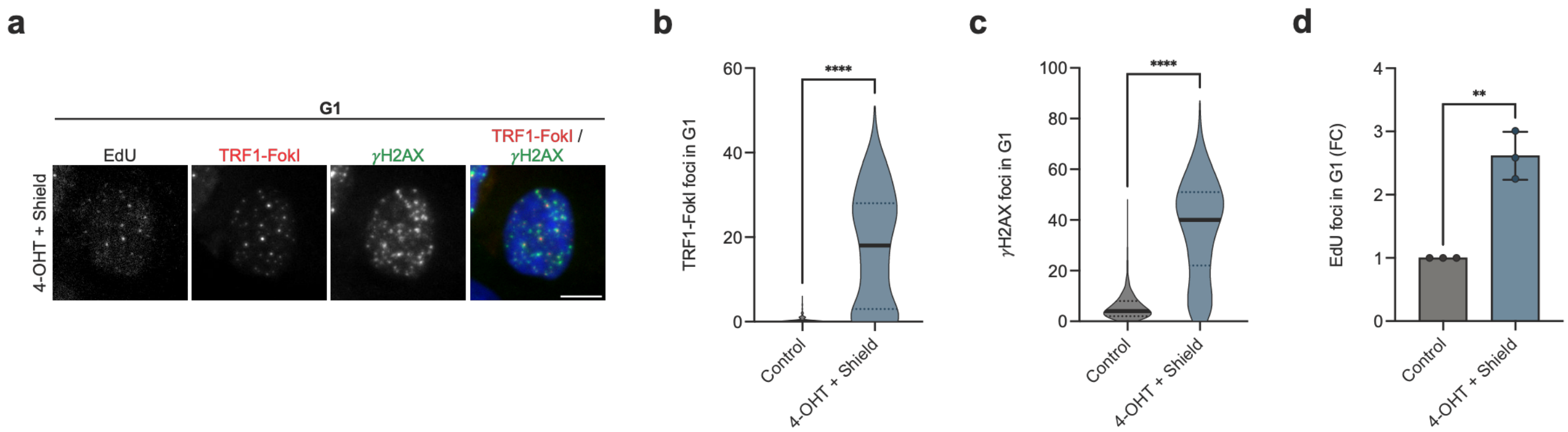

Supplementary Figure 3: TRF1-FokI induces DNA damage and post-MiDAS at telomeres. a Representative images of EdU, TRF1-FokI, and γH2AX foci co-localization in G1-phase U-2 OS TRF1-FokI cells treated with 4-OHT (1 µM) and Shield-1 (1 µM) for 2 hours. **b, c** Violin plots of TRF1-FokI foci count (b) and γH2AX foci count (c) per G1-phase U-2 OS TRF1-FokI nucleus untreated or treated with 4-OHT (1 µM) and Shield-1 (1 µM) for 2 hours (n = 490 (b) and n = 900 (c); n values indicate cells per experiment). Solid lines represent the median; dashed lines indicate the upper and lower quartiles. **** $p < 0.0001$ (two-tailed unpaired t-test). **d** Normalized EdU foci count (fold change (FC)) per G1-phase U-2 OS TRF1-FokI nucleus treated as in (b). Means ± SD of three independent experiments normalized to the untreated control are shown (n = 100 cells per experiment). ** $p = 0.0018$ (two-tailed unpaired t-test). Scale bar, 10 µm. Source data are provided as a Source Data file.

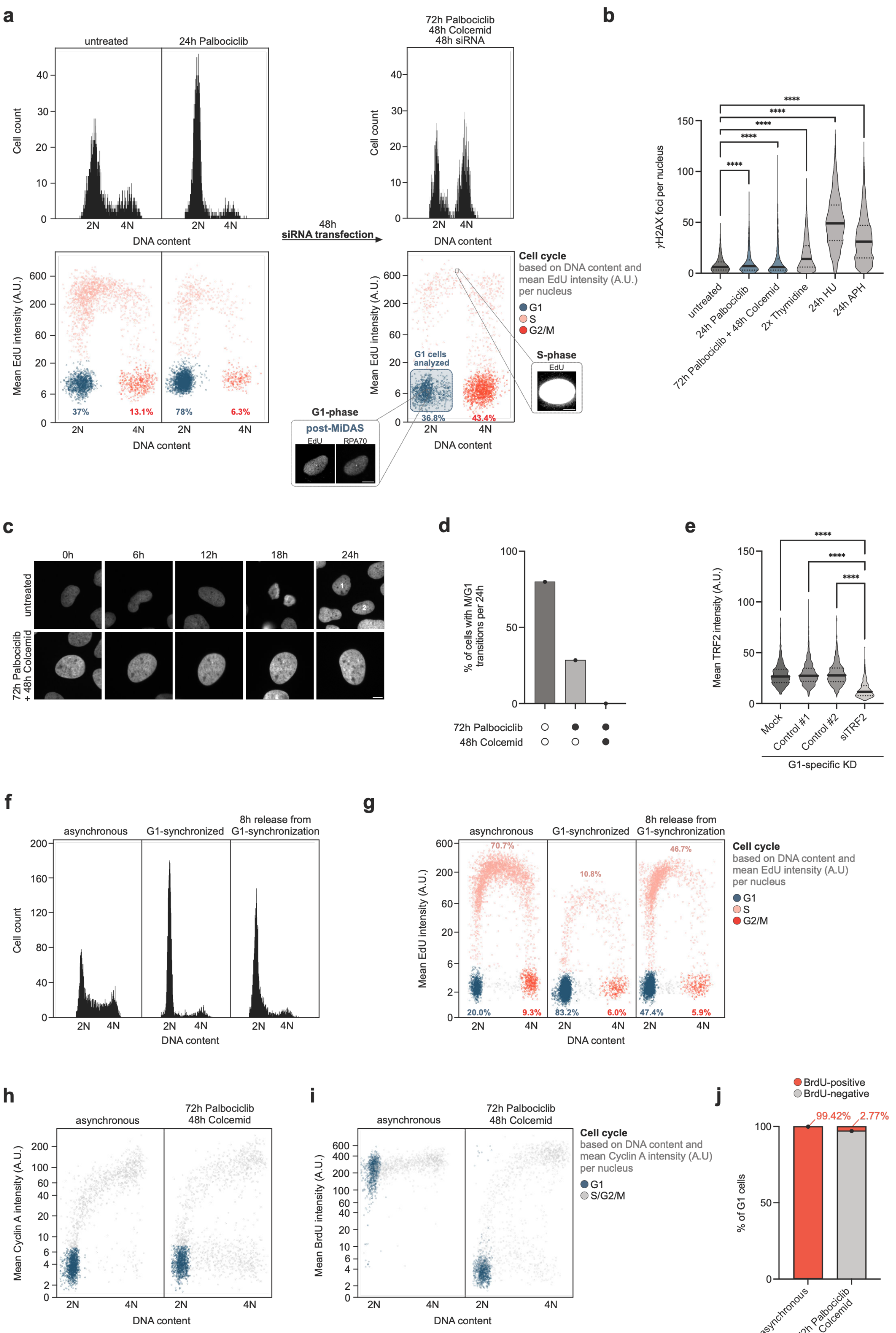

a
untreated
24h Palbociclib
Cell count
DNA content
Mean EdU intensity (A.U.)
37%
13.1%
78%
6.3%
48h
siRNA transfection
72h Palbociclib
48h Colcemid
48h siRNA
Cell cycle
based on DNA content and mean EdU intensity (A.U.) per nucleus
G1
S
G2/M
S-phase
EdU
G1-phase
post-MiDAS
EdU
RPA70
G1 cells analyzed
36.8%
43.4%
b
γH2AX foci per nucleus
****
untreated
24h Palbociclib
72h Palbociclib + 48h Colcemid
2x Thymidine
24h HU
24h APH
c
0h
6h
12h
18h
24h
untreated
72h Palbociclib + 48h Colcemid
d
% of cells with M/G1 transitions per 24h
72h Palbociclib
48h Colcemid
e
Mean TRF2 intensity (A.U.)
Mock
Control #1
Control #2
siTRF2
G1-specific KD
f
asynchronous
G1-synchronized
8h release from G1-synchronization
Cell count
DNA content
g
70.7%
10.8%
46.7%
20.0%
9.3%
83.2%
6.0%
47.4%
5.9%
Mean EdU intensity (A.U.)
Cell cycle
based on DNA content and mean EdU intensity (A.U) per nucleus
G1
S
G2/M
h
asynchronous
72h Palbociclib
48h Colcemid
Mean Cyclin A intensity (A.U.)
DNA content
i
Mean BrdU intensity (A.U.)
Cell cycle
based on DNA content and mean Cyclin A intensity (A.U) per nucleus
G1
S/G2/M
j
BrdU-positive
BrdU-negative
99.42%
2.77%
% of G1 cells
asynchronous
72h Palbociclib
48h Colcemid

Supplementary Figure 4: Experimental validation of the G1-targeted siRNA screening strategy. a High-content microscopy-derived cell cycle profiles based on DNA content (total DAPI intensity (A.U.)) and EdU incorporation (mean EdU intensity (A.U.)) per U-2 OS nucleus. Cells were untreated, arrested in G1 with Palbociclib (1 µM) for 24 hours, or treated with Palbociclib (1 µM) for 24 hours followed by 48-hour mock siRNA transfection in the presence of colcemid (0.1 µg/ml) and Palbociclib (1 µM). EdU (100 µM) was added during the last hour before fixation. The color code indicates cell cycle phase defined by DNA content and mean EdU intensity (A.U.) (n = 2800 cells per condition). The analyzed G1 cell population is highlighted, and representative S-phase and post-MiDAS-positive G1-phase U-2 OS cells with EdU/RPA70 co-localization are shown. **b** Violin plot of γH2AX foci count per nucleus in untreated or G1-synchronized U-2 OS cells using Palbociclib (1 µM) for 24 hours, Palbociclib (1 µM) for 72 hours with colcemid (0.1 µg/ml) added during the last 48 hours, a double thymidine block (18-hour thymidine (2 mM), 8-hour release, 16-hour thymidine treatment (2 mM)), HU (4 mM) for 24 hours, or APH (20 µM) for 24 hours (n = 4000 cells per condition). Solid lines represent the median; dashed lines indicate the upper and lower quartiles. **** $p < 0.0001$ (one-way ANOVA with Tukey's multiple-comparison test). **c** Representative time-lapse images of U-2 OS H2B-GFP cells untreated or treated with Palbociclib (1 µM) for 72 hours with colcemid (0.1 µg/ml) added during the last 48 hours. Cell cycle synchronization in this way was performed in > 3 independent experiments; time-lapse imaging under these conditions was performed once. **d** Percentage of U-2 OS H2B-GFP cells undergoing M/G1 transitions during 24 hours of time-lapse imaging. Cells were untreated, treated with Palbociclib (1 µM) for 72 hours, or treated as in (c) (n = 500 cells per condition). **e** Violin plot of mean TRF2 intensity (A.U.) in U-2 OS cells following G1-specific transfection with no siRNA (Mock), control siRNAs (Control #1 and #2), or TRF2 siRNA from the targeted siRNA screen (n = 700 cells per condition). Solid lines represent the median; dashed lines indicate the upper and lower quartiles. **** $p < 0.0001$ (one-way ANOVA with Tukey's multiple-comparison test). **f, g** Cell cycle profiles based on DNA content (total DAPI intensity (A.U.)) (f) or DNA content and EdU incorporation (mean EdU intensity (A.U.)) per U-2 OS nucleus (g). Cells were untreated, arrested in G1 with Palbociclib (1 µM) for 72 hours, or treated with Palbociclib (1 µM) for 72 hours followed by an 8-hour release. The color code and percentages in (g) indicate cell cycle phase defined by DNA content and mean EdU intensity (A.U.) (n = 3900 cells per condition). **h, i** Cell cycle profiles based on DNA content (total DAPI intensity (A.U.)) and mean Cyclin A intensity (A.U.) (h) or BrdU incorporation (mean BrdU intensity (A.U.)) (i) per U-2 OS nucleus. Cells were treated as in (c). BrdU (10 µM) was added during the last 48 hours before fixation in both conditions. The color code indicates cell cycle phase defined by DNA content and mean Cyclin A intensity (A.U.) (n = 2100 cells per condition). **(j)** Percentage of BrdU-positive and -negative G1-phase U-2 OS cells. G1-phase cells were identified based on 2N DNA content and the absence of detectable Cyclin A expression. Cells were treated as in (h, i) (n = 2100 cells per condition). A.U., arbitrary units. Scale bars, 10 µm. Source data are provided as a Source Data file.

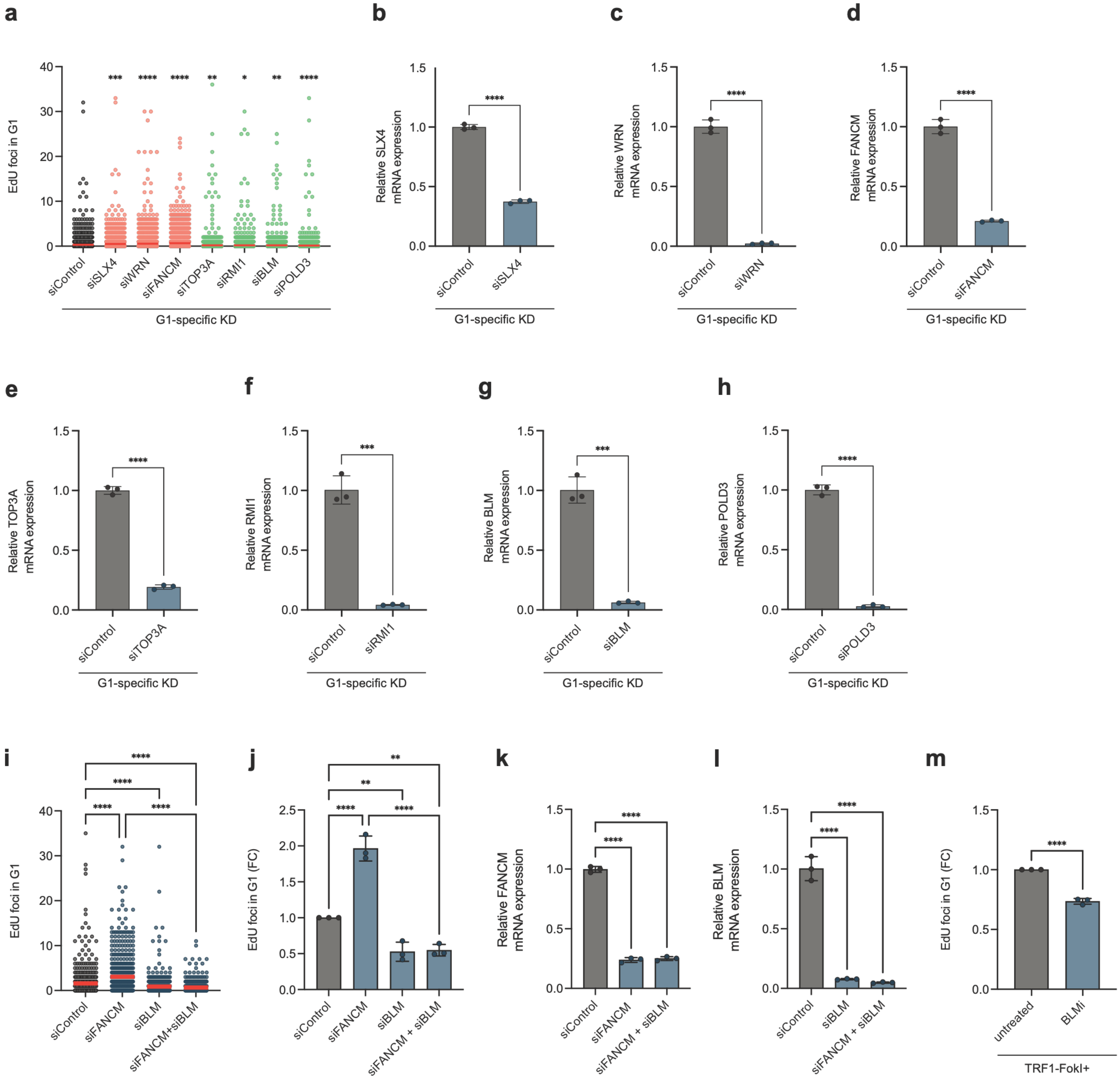

Supplementary Figure 5: ALT-associated factors regulate post-MiDAS. a EdU foci count per G1-phase U-2 OS nucleus following G1-specific transfection with control, SLX4, WRN, FANCM, TOP3A, RMI1, BLM, or POLD3 siRNA. Scatterplots show $n = 2700$ cells per condition. Red lines represent the mean. *** (siSLX4) $p = 0.0003$, **** (siWRN) $p < 0.0001$, **** (siFANCM) $p < 0.0001$, ** (siTOP3A) $p = 0.0045$, * (siRMI1) $p = 0.0155$, ** (siBLM) $p = 0.0051$, and **** (siPOLD3) $p < 0.0001$ (one-way ANOVA with Dunnett's multiple-comparison test). **b-h** Relative SLX4 (b), WRN (c), FANCM (d), TOP3A (e), RMI1 (f), BLM (g), and POLD3 (h) mRNA expression in G1-arrested U-2 OS cells following G1-specific transfection with control or the indicated siRNAs. Individual values and means ± SD normalized to the siControl condition are shown. **** $p < 0.0001$ (b–e, h) and *** $p = 0.0001$ (f, g) (two-tailed unpaired *t*-test). **i** EdU foci count per G1-phase U-2 OS nucleus following transfection with control, FANCM, BLM, or FANCM+BLM siRNA. Scatterplots show $n = 650$ cells per condition. Red lines represent the mean. **** $p < 0.0001$ (one-way ANOVA with Tukey's multiple-comparison test). **j** Normalized EdU foci count (FC) per G1-phase U-2 OS nucleus following transfection with control, FANCM, BLM, or FANCM+BLM siRNAs. Means ± SD of three independent experiments normalized to the siControl condition are shown ($n = 650$ cells per experiment). **** (siControl vs. siFANCM) $p < 0.0001$, ** (siControl vs. siBLM) $p = 0.0049$, ** (siControl vs. siFANCM + siBLM) $p = 0.0064$, and **** (siFANCM vs. siFANCM + siBLM) $p < 0.0001$ (one-way ANOVA with Tukey's multiple-comparison test). **k, l** Relative FANCM (k) and BLM (l) mRNA expression in

U-2 OS cells following transfection with control, FANCM, BLM, or FANCM+BLM siRNA. Individual values and means ± SD normalized to the siControl condition are shown. **** $p < 0.0001$ (one-way ANOVA with Tukey's multiple-comparison test). **m** Normalized EdU foci count (FC) per TRF1-FokI foci-positive G1-phase U-2 OS TRF1-FokI nucleus untreated or treated with BLMi (BLM-IN-1, 2 μM) for 2 hours (n = 500 cells per experiment). Means ± SD of three independent experiments normalized to the untreated control are shown. **** $p < 0.0001$ (two-tailed unpaired t-test). Source data are provided as a Source Data file.

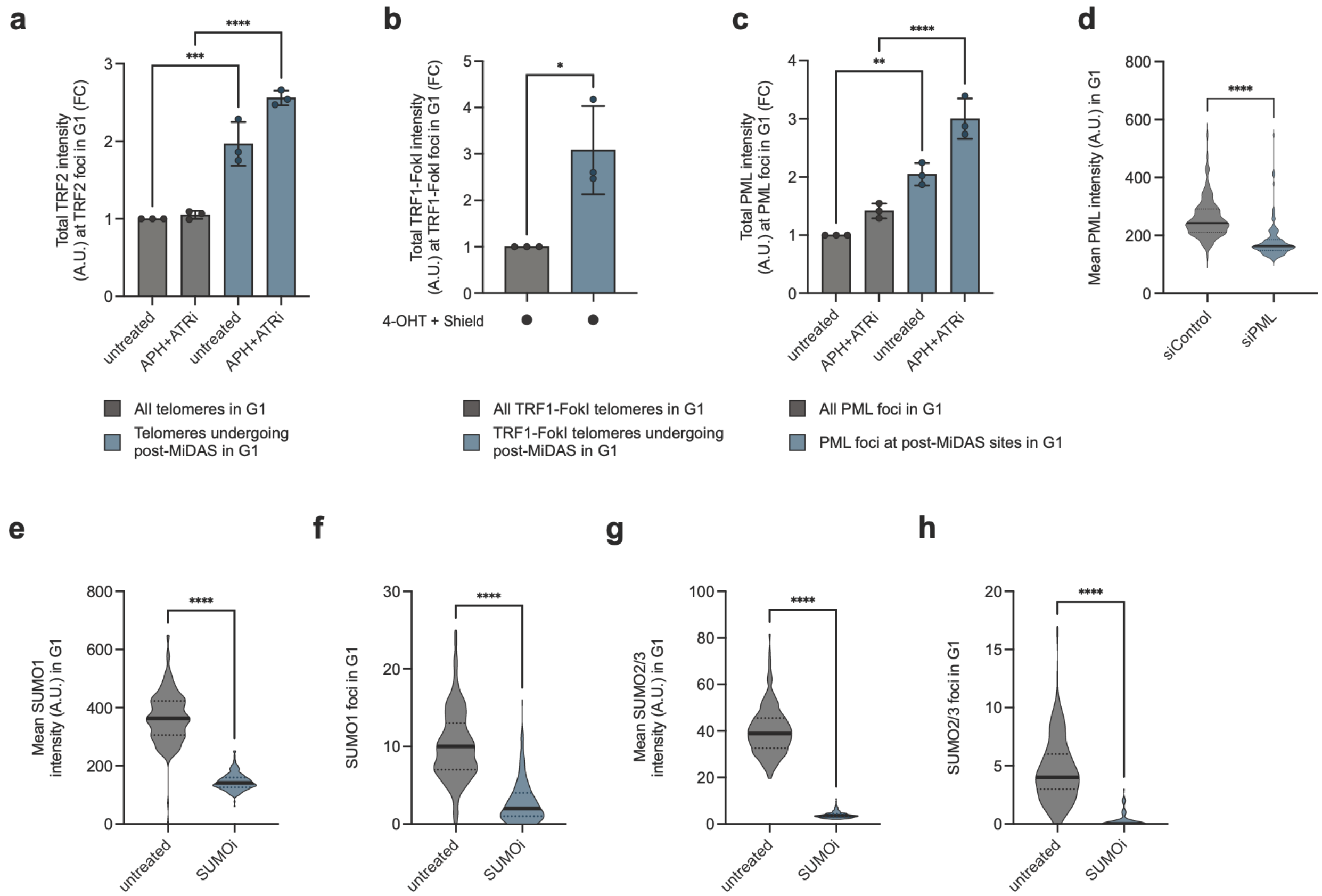

Supplementary Figure 6: SUMOylation and telomere clustering in APBs during post-MiDAS. a Normalized total TRF2 intensity (A.U.) (FC) at EdU-positive and all detected TRF2 foci in G1-phase U-2 OS cells left untreated or treated with APH (0.2 µM) and ATRi (1 µM) for 24 hours (n_1 = 58, n_2 = 114, and n_3 = 137 foci per experiment). Means ± SD of three independent experiments normalized to the untreated control (all detected TRF2 foci in G1) are shown. *** $p = 0.0002$ and **** $p < 0.0001$ (one-way ANOVA with Tukey's multiple-comparison test). **b** Normalized total TRF1-FokI intensity (A.U.) (FC) at EdU-positive and all detected TRF1-FokI foci in G1-phase U-2 OS TRF1-FokI cells treated with 4-OHT (1 µM) and Shield-1 (1 µM) for 2 hours (n = 100 foci per experiment). Means ± SD of three independent experiments normalized to the average total TRF1-FokI foci intensity (A.U.) across all detected TRF1-FokI foci in G1 are shown. * p = 0.0192 (two-tailed unpaired *t*-test). **c** Normalized total PML intensity (A.U.) (FC) at EdU-positive and all detected PML foci in G1-phase U-2 OS cells treated as in (a). Means ± SD of three independent experiments normalized to the untreated control (all detected PML foci in G1) are shown (n_1 = 150, n_2 = 157, and n_3 = 178 foci per experiment). ** p = 0.0013 and **** $p < 0.0001$ (one-way ANOVA with Tukey's multiple-comparison test). **d** Violin plot of mean PML intensity (A.U.) per G1-phase U-2 OS nucleus following transfection with control or PML siRNA (n = 151 cells per condition). Solid lines represent the median; dashed lines indicate the upper and lower quartiles. **** $p < 0.0001$ (two-tailed unpaired *t*-test). **e-h** Violin plots of mean SUMO1 intensity (A.U.) (e), SUMO1 foci count (f), mean SUMO2/3 intensity (A.U.) (g), and SUMO2/3 foci count (h) per G1-phase U-2 OS nucleus. Cells were left untreated or treated with SUMOi (ML-792, 1 µM) for 2 hours (n = 200 cells per condition). Solid lines represent the median; dashed lines indicate the upper and lower quartiles. **** $p < 0.0001$ (two-tailed unpaired *t*-test). A.U., arbitrary units. Source data are provided as a Source Data file.

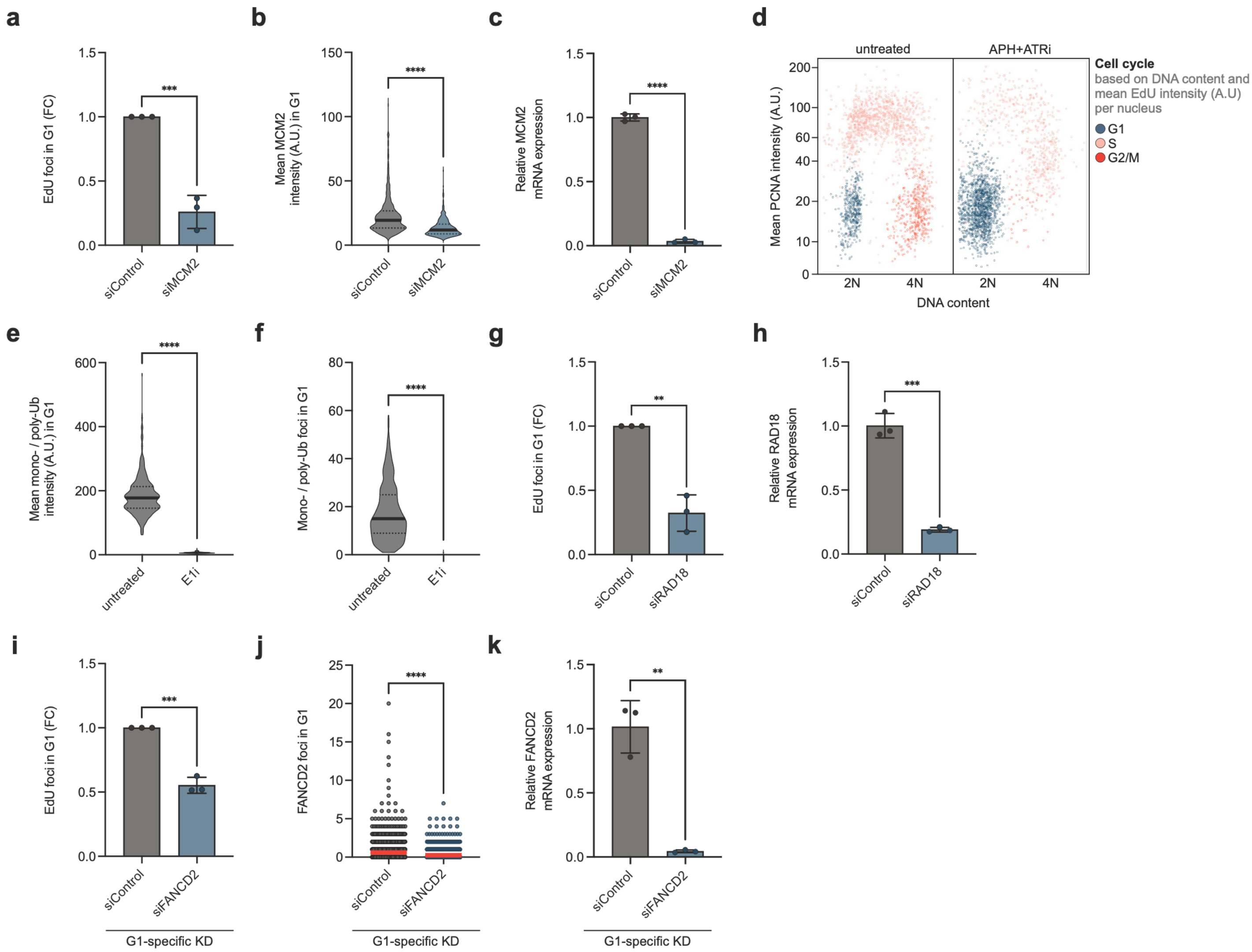

Supplementary Figure 7: Factors associated with MiDAS and DNA damage tolerance contribute to post-MiDAS. a Normalized EdU foci count (FC) per G1-phase U-2 OS nucleus following transfection with control or MCM2 siRNA. Means ± SD of three independent experiments normalized to the siControl condition are shown ($n_1 = 2000$, $n_2 = 2000$, and $n_3 = 480$ cells per experiment). *** $p = 0.0006$ (two-tailed unpaired *t*-test). **b** Violin plot of mean MCM2 intensity (A.U.) per G1-phase U-2 OS nucleus following transfection with control or MCM2 siRNA ($n = 480$ cells per condition). Solid lines represent the median; dashed lines indicate the upper and lower quartiles. **** $p < 0.0001$ (two-tailed unpaired *t*-test). **c** Relative MCM2 mRNA expression in U-2 OS cells following transfection with control or MCM2 siRNA. Individual values and means ± SD normalized to the siControl condition are shown. **** $p < 0.0001$ (two-tailed unpaired *t*-test). **d** High-content microscopy-derived cell cycle-resolved mean PCNA intensity (A.U.) in pre-extracted U-2 OS cells based on DNA content (total DAPI intensity (A.U.)) per nucleus. Cells were left untreated or treated with APH (0.2 µM) and ATRi (1 µM) for 24 hours ($n = 1984$ cells per condition). Color coding indicates the cell cycle phase defined by DNA content and mean EdU intensity per nucleus. **e-f** Violin plot of mean mono- and polyubiquitin conjugate intensity (A.U.) (e) and mono- and polyubiquitin conjugate foci count (f) per G1-phase U-2 OS nucleus. Cells were either untreated or treated with E1i (TAK-243, 1 µM) for 4 hours ($n = 400$ cells per condition). Solid lines represent the median; dashed lines indicate the upper and lower quartiles. **** $p < 0.0001$ (two-tailed unpaired *t*-test). **g** Normalized EdU foci count (FC) per G1-phase U-2 OS nucleus following transfection with control or RAD18 siRNA. Means ± SD of three independent experiments normalized to the siControl condition are shown ($n_1 = 3000$, $n_2 = 2400$, and $n_3 = 3000$ cells per experiment). ** $p = 0.0012$ (two-tailed unpaired *t*-test). **h** Relative RAD18 mRNA expression in U-2 OS cells following transfection with control or RAD18 siRNA. Individual values and means ± SD normalized to the siControl condition are shown. *** $p = 0.0001$ (two-tailed unpaired *t*-test). **i** Normalized EdU foci count (FC) per G1-phase U-2 OS nucleus following G1-specific transfection with control or FANCD2 siRNA. Means ± SD of three independent experiments normalized to the siControl condition are shown ($n_1 =$

2700, $n_2 = 2300$, and $n_3 = 2300$ cells per experiment). *** $p = 0.0002$ (two-tailed unpaired *t*-test). **j** FANCD2 foci count per G1-phase U-2 OS nucleus following G1-specific transfection with control or FANCD2 siRNA. Scatterplots show $n = 2300$ cells per condition. Red lines represent the mean. **** $p < 0.0001$ (two-tailed unpaired *t*-test). **k** Relative FANCD2 mRNA expression in G1-arrested U-2 OS cells following G1-specific transfection with control or FANCD2 siRNA. Individual values and means ± SD normalized to the siControl condition are shown. ** $p = 0.0012$ (two-tailed unpaired *t*-test). A.U., arbitrary units. Source data are provided as a Source Data file.

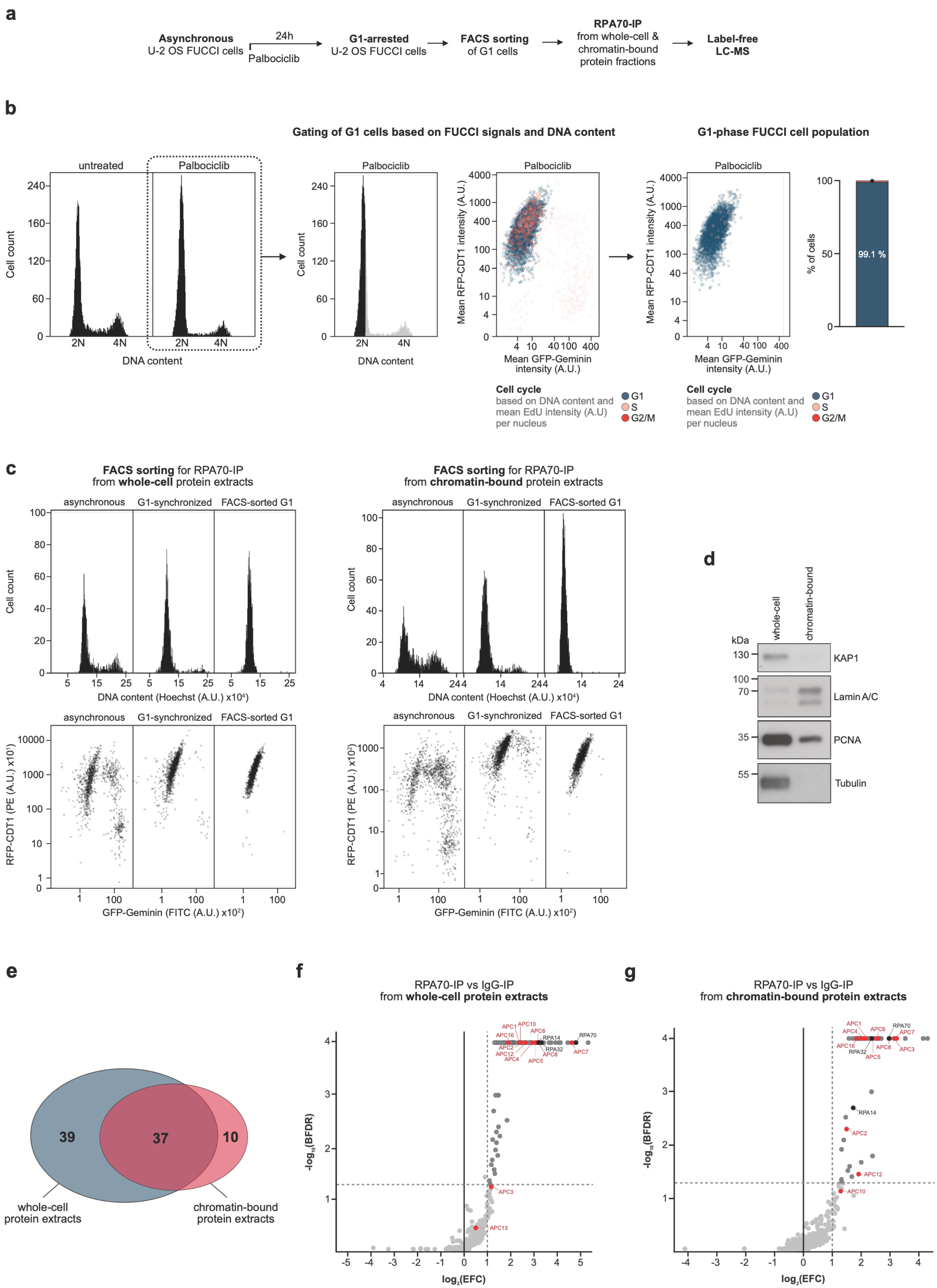

a
Asynchronous U-2 OS FUCCI cells
24h
Palbociclib
G1-arrested U-2 OS FUCCI cells
FACS sorting of G1 cells
RPA70-IP from whole-cell & chromatin-bound protein fractions
Label-free LC-MS
b
Gating of G1 cells based on FUCCI signals and DNA content
G1-phase FUCCI cell population
untreated
Palbociclib
Cell count
DNA content
Mean RFP-CDT1 intensity (A.U.)
Mean GFP-Geminin intensity (A.U.)
% of cells
99.1 %
Cell cycle
based on DNA content and mean EdU intensity (A.U) per nucleus
G1
S
G2/M
c
FACS sorting for RPA70-IP from whole-cell protein extracts
FACS sorting for RPA70-IP from chromatin-bound protein extracts
asynchronous
G1-synchronized
FACS-sorted G1
DNA content (Hoechst (A.U.) x10⁴)
RFP-CDT1 (PE (A.U.) x10¹)
RFP-CDT1 (PE (A.U.) x10²)
GFP-Geminin (FITC (A.U.) x10²)
d
whole-cell
chromatin-bound
kDa
KAP1
Lamin A/C
PCNA
Tubulin
e
39
37
10
whole-cell protein extracts
chromatin-bound protein extracts
f
RPA70-IP vs IgG-IP from whole-cell protein extracts
g
RPA70-IP vs IgG-IP from chromatin-bound protein extracts
-log10(BFDR)
log2(EFC)

Supplementary Figure 8: Targeted quantitative proteomics to identify the RPA interactome in G1. a Schematic of the RPA70-IP MS experiment from whole-cell and chromatin-bound protein extracts of FACS-sorted G1-phase U-2 OS FUCCI cells. **b** High-content microscopy analysis of asynchronously growing (untreated) and G1-synchronized U-2 OS FUCCI cells (treated with Palbociclib (1 µM) for 24 hours), illustrating the gating strategy used to sort a pure G1-phase cell population by three-parameter FACS. G1-phase cells were identified by 2N DNA content and FUCCI signals (high RFP-CDT1, low GFP-Geminin). The G1 cell population selected for sorting is highlighted, and the bar chart shows the percentage of G1 cells within this population. The color code indicates the cell cycle phase defined by DNA content and mean EdU intensity (A.U.) per nucleus (n = 5600 cells per condition). **c** FACS analysis of asynchronously growing (untreated), G1-synchronized (treated with Palbociclib (1 µM) for 24 hours), and FACS-sorted G1-phase U-2 OS FUCCI cells based on DNA content staining with Hoechst 33342 and FUCCI signals (n = 1121 (asynchronous, whole-cell protein extracts), n = 1208 (G1-synchronized, whole-cell protein extracts), n = 1290 (FACS-sorted G1, whole-cell protein extracts), n = 1222 (asynchronous, chromatin-bound protein extracts), n = 1402 (G1-synchronized, chromatin-bound extracts), and n = 1430 (FACS-sorted G1, chromatin-bound protein extracts); n values indicate cells per condition). **d** Western blot analysis of KAP1, Lamin A/C, PCNA, and Tubulin levels in whole-cell and chromatin-bound protein extracts from U-2 OS cells. **e** Venn diagram showing significantly enriched proteins (EFC ≥ 2; BFDR ≤ 0.05) identified by RPA70-IP MS from whole-cell and chromatin-bound protein extracts of FACS-sorted G1-phase U-2 OS FUCCI cells. **f** Scatterplot of RPA70 interactors in G1 identified by RPA70-IP MS from whole-cell protein extracts of FACS-sorted G1-phase U-2 OS FUCCI cells. Data from quadruplicate samples per condition were analyzed by SAINT. Significantly enriched proteins (EFC ≥ 2; BFDR ≤ 0.05) are highlighted in dark grey. Components of the trimeric RPA complex and the APC/C are highlighted in black and red, respectively. **g** Scatterplot of RPA70 interactors in G1 identified by RPA70-IP LC-MS from chromatin-bound protein extracts of FACS-sorted G1-phase U-2 OS FUCCI cells. Data from quadruplicate samples per condition were analyzed by SAINT. Significantly enriched proteins (EFC ≥ 2; BFDR ≤ 0.05) are highlighted in dark grey. Components of the trimeric RPA complex and the APC/C are highlighted in black and red, respectively. A.U., arbitrary units. Source data are provided as a Source Data file.

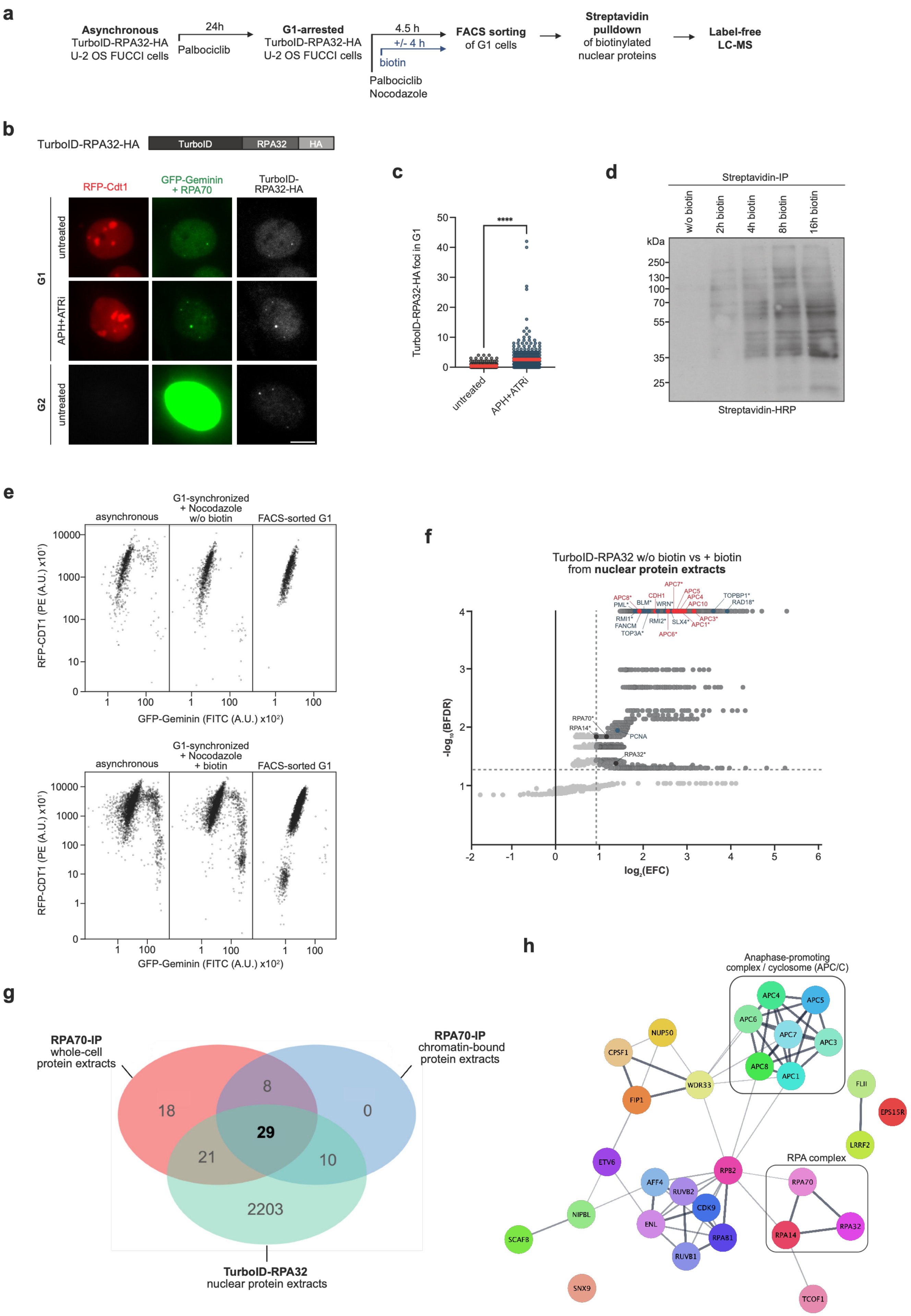
a
Asynchronous
TurboID-RPA32-HA
U-2 OS FUCCI cells
24h
Palbociclib
G1-arrested
TurboID-RPA32-HA
U-2 OS FUCCI cells
4.5 h
+/- 4 h
biotin
Palbociclib
Nocodazole
FACS sorting
of G1 cells
Streptavidin
pulldown
of biotinylated
nuclear proteins
Label-free
LC-MS
b
TurboID-RPA32-HA
TurboID
RPA32
HA
RFP-Cdt1
GFP-Geminin
+ RPA70
TurboID-
RPA32-HA
G1
untreated
APH+ATRi
G2
untreated
c
TurboID-RPA32-HA foci in G1
****
untreated
APH+ATRi
d
Streptavidin-IP
w/o biotin
2h biotin
4h biotin
8h biotin
16h biotin
kDa
250
130
100
70
55
35
25
Streptavidin-HRP
e
asynchronous
G1-synchronized
+ Nocodazole
w/o biotin
FACS-sorted G1
RFP-CDT1 (PE (A.U.) x10¹)
GFP-Geminin (FITC (A.U.) x10²)
asynchronous
G1-synchronized
+ Nocodazole
+ biotin
FACS-sorted G1
RFP-CDT1 (PE (A.U.) x10¹)
GFP-Geminin (FITC (A.U.) x10²)
f
TurboID-RPA32 w/o biotin vs + biotin
from nuclear protein extracts
APC7*
APC8*
CDH1
APC5
APC4
TOPBP1*
PML*
BLM*
WRN*
APC10
RAD18*
RMI1*
FANCM
TOP3A*
RMI2*
SLX4*
APC3*
APC1*
APC6*
RPA70*
RPA14*
PCNA
RPA32*
-log₁₀(BFDR)
log₂(EFC)
g
RPA70-IP
whole-cell
protein extracts
RPA70-IP
chromatin-bound
protein extracts
18
8
0
29
21
10
2203
TurboID-RPA32
nuclear protein extracts
h
Anaphase-promoting
complex / cyclosome (APC/C)
APC4
APC5
APC6
APC7
APC3
APC8
APC1
NUP50
CPSF1
WDR33
FIP1
FLII
EPS15R
LRRF2
ETV6
RPB2
RPA complex
RPA70
AFF4
RUVB2
CDK9
NIPBL
ENL
RPAB1
RPA32
RPA14
SCAF8
RUVB1
SNX9
TCOF1

Supplementary Figure 9: TurboID labeling proteomics to identify the proximity proteome of RPA in G1. a Schematic of the RPA32-TurboID LC-MS experiment from nuclear protein extracts of FACS-sorted G1-phase U-2 OS FUCCI TurboID-RPA32-HA cells. **b** Schematic of the TurboID-RPA32-HA construct expressed in U-2 OS FUCCI cells. Representative images of TurboID-RPA32-HA and RPA70 co-localization in G1- and G2-phase U-2 OS FUCCI TurboID-RPA32-HA cells untreated or treated with APH (0.2 μM) and ATRi (1 μM) for 24 hours. **c** TurboID-RPA32-HA foci count per G1-phase U-2 OS FUCCI TurboID-RPA32-HA nucleus as in (b). Scatterplots show $n = 320$ cells per condition. Red lines represent the mean. **** $p < 0.0001$ (two-tailed unpaired t-test). **d** Western blot analysis of streptavidin pulldowns of biotinylated proteins from U-2 OS FUCCI TurboID-RPA32-HA cells detected with Streptavidin-HRP. Biotin labeling (500 μM) was performed for 2, 4, 8, and 16 hours and compared to an unlabeled control (without (w/o) biotin). The biotinylation time course was performed once. **e** FACS analysis of asynchronously growing, G1-synchronized (treated with Palbociclib (1 μM) for 24 hours), and FACS-sorted G1-phase U-2 OS FUCCI TurboID-RPA32-HA cells. After the 24-hour Palbociclib treatment, cells were treated with Palbociclib (1 μM) and nocodazole (50 ng/ml) for 4 hours with or without biotin (500 μM) before FACS sorting. G1 cells were identified by DNA content staining with Hoechst 33342 and FUCCI signals (high RFP-CDT1, low GFP-Geminin). Upper and lower panels show conditions without and with biotin labeling, respectively (n = 1005 (asynchronous, without biotin labeling), n = 999 (G1-synchronized, without biotin labeling), n = 1010 (FACS-sorted G1, without biotin labeling), n = 3807 (asynchronous, with biotin labeling), n = 4077 (G1-synchronized, with biotin labeling), and n = 4290 (FACS-sorted G1, with biotin labeling); n values indicate cells per condition). **f** Scatterplot of RPA32 interactors in G1 identified by TurboID-RPA32 LC-MS from nuclear protein extracts of FACS-sorted G1-phase U-2 OS FUCCI TurboID-RPA32-HA cells. Data from duplicate samples per condition were analyzed by SAINT. Significantly enriched proteins (EFC ≥ 1.9; BFDR ≤ 0.05) are highlighted in dark grey. Trimeric RPA complex components, APC/C components, and additional post-MiDAS factors are highlighted in black, red, and dark blue, respectively. * indicates highlighted proteins also significantly enriched in a second, independent TurboID-RPA32 LC-MS dataset generated from nuclear protein extracts of duplicate samples per condition. **g** Venn diagram showing significantly enriched proteins identified by RPA70-IP LC-MS analysis of whole-cell and chromatin-bound protein extracts of FACS-sorted G1-phase U-2 OS FUCCI cells (EFC ≥ 2; BFDR ≤ 0.05) and by RPA32-TurboID LC-MS analysis of nuclear protein extracts of FACS-sorted G1-phase U-2 OS FUCCI TurboID-RPA32-HA cells (EFC ≥ 1.9; BFDR ≤ 0.05; peptide count ≥ 4). **h** STRING protein interaction network of the 29 overlapping significantly enriched RPA interactors in G1 identified by RPA70-IP LC-MS of whole-cell and chromatin-bound protein extracts and by RPA32-TurboID LC-MS of nuclear protein extracts of FACS-sorted G1-phase cells. A.U., arbitrary units. Scale bar, 10 μm. Source data are provided as a Source Data file.

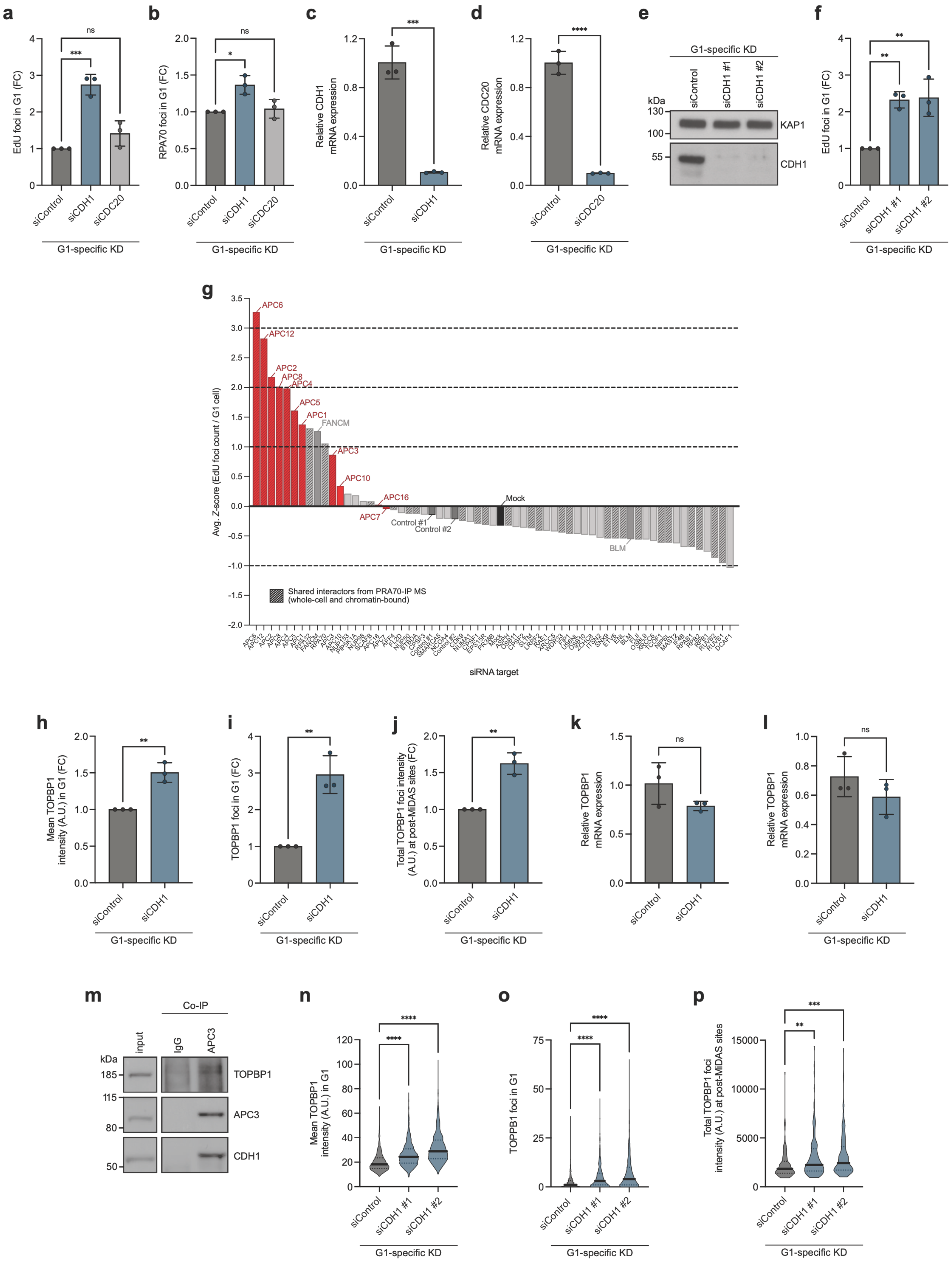
a
ns
***
EdU foci in G1 (FC)
siControl
siCDH1
siCDC20
G1-specific KD
b
ns
*
RPA70 foci in G1 (FC)
siControl
siCDH1
siCDC20
G1-specific KD
c
***
Relative CDH1 mRNA expression
siControl
siCDH1
G1-specific KD
d
****
Relative CDC20 mRNA expression
siControl
siCDC20
G1-specific KD
e
G1-specific KD
kDa
siControl
siCDH1 #1
siCDH1 #2
KAP1
CDH1
f
**
**
EdU foci in G1 (FC)
siControl
siCDH1 #1
siCDH1 #2
G1-specific KD
g
APC6
APC12
APC2
APC8
APC4
APC5
APC1
FANCM
APC3
APC10
APC16
APC7
Control #1
Control #2
Mock
BLM
Avg. Z-score (EdU foci count / G1 cell)
Shared interactors from PRA70-IP MS (whole-cell and chromatin-bound)
siRNA target
h
**
Mean TOPBP1 intensity (A.U.) in G1 (FC)
siControl
siCDH1
G1-specific KD
i
**
TOPBP1 foci in G1 (FC)
siControl
siCDH1
G1-specific KD
j
**
Total TOPBP1 foci intensity (A.U.) at post-MiDAS sites (FC)
siControl
siCDH1
G1-specific KD
k
ns
Relative TOPBP1 mRNA expression
siControl
siCDH1
l
ns
Relative TOPBP1 mRNA expression
siControl
siCDH1
G1-specific KD
m
Co-IP
input
IgG
APC3
kDa
TOPBP1
APC3
CDH1
n
****
****
Mean TOPBP1 intensity (A.U.) in G1
siControl
siCDH1 #1
siCDH1 #2
G1-specific KD
o
****
****
TOPPB1 foci in G1
siControl
siCDH1 #1
siCDH1 #2
G1-specific KD
p
***
**
Total TOPBP1 foci intensity (A.U.) at post-MiDAS sites
siControl
siCDH1 #1
siCDH1 #2
G1-specific KD

Supplementary Figure 10: APC/C^{CDH1} restrains excessive post-MiDAS in G1. a, b Normalized EdU (a) and RPA70 (b) foci count (FC) per G1-phase U-2 OS nucleus following G1-specific transfection with control, CDH1, or CDC20 siRNA. Means ± SD of three independent experiments normalized to the siControl condition are shown (n = 100 cells per experiment). *** p = 0.0004 and ns p = 0.2011 (a), and * p = 0.0115 and ns p = 0.8810 (b) (one-way ANOVA with Tukey's multiple-comparison test). **c, d** Relative CDH1 (c) and CDC20 (d) mRNA expression in G1-arrested U-2 OS cells following G1-specific transfection with control or the indicated siRNAs. Individual values and means ± SD normalized to the siControl condition are shown. *** p = 0.0003 (c) and **** p < 0.0001 (d) (two-tailed unpaired *t*-test). **e** Western blot analysis of CDH1 and KAP1 protein levels in whole-cell lysates of G1-arrested U-2 OS cells following G1-specific transfection with control siRNA or two independent CDH1 siRNAs (CDH1 #1 and #2). The knockdown validation by Western blot was performed once. **f** Normalized EdU foci count (FC) per G1-phase U-2 OS nucleus following G1-specific transfection with control siRNA or two independent CDH1 siRNAs (CDH1 #1 and #2). Means ± SD of three independent experiments normalized to the siControl condition are shown (n = 100 cells per experiment). ** (siCDH1 #1) p = 0.0055 and ** (siCDH1 #2) p = 0.0044 (one-way ANOVA with Tukey's multiple-comparison test). **g** Ranked average *Z*-scores of the average EdU foci count per G1-phase U-2 OS nucleus following the siRNA-mediated knockdown of 60 different target genes identified as RPA70 interactors by RPA70-IP LC-MS using 2-3 independent siRNAs per target, including FANCM- and BLM-targeting siRNAs as positive controls. Negative control siRNAs (Control #1 and #2) and a mock transfection were included (n > 680 cells per condition). Shared interactors identified in both RPA70-IP LC-MS datasets (whole-cell and chromatin-bound protein extracts) are indicated by striped bars, and APC/C components are highlighted in red. **h-j** Normalized mean TOPBP1 intensity (A.U.) (FC) (h), average TOPBP1 foci count (FC) (i), and total TOPBP1 foci intensity (A.U.) (FC) at post-MiDAS sites (j) per G1-phase U-2 OS nucleus following G1-specific transfection with control or CDH1 siRNA. Means ± SD of three independent experiments normalized to the siControl condition are shown (n_1 = 300, n_2 = 380, and n_3 = 520 cells per experiment (h, i); n = 70 foci per experiment (j)). ** p = 0.0028 (h), ** p = 0.0027 (i), and ** p = 0.0018 (j) (two-tailed unpaired *t*-test). **k, l** Relative TOPBP1 mRNA expression in asynchronous (k) and G1-arrested (l) U-2 OS cells following transfection with control or CDH1 siRNA. Individual values and means ± SD normalized to the siControl condition are shown. ns p = 0.1431 (k) and ns p = 0.2576 (l) (two-tailed unpaired *t*-test). **m** Western blot analysis after immunoprecipitation of endogenous APC3 from U-2 OS cells. Control IgG and APC3 immunoprecipitates were probed for endogenous APC3, TOPBP1, and CDH1. **n-p** Violin plot of mean TOPBP1 intensity (A.U.) per G1-phase U-2 OS nucleus (n), TOPBP1 foci count per G1-phase U-2 OS nucleus (o), and total TOPBP1 foci intensity (A.U.) at post-MiDAS sites in G1 (p) following G1-specific transfection with control siRNA or two independent siRNAs targeting CDH1 (CDH1 #1 and #2) (n = 750 cells (n, o) and n = 140 foci (p) per condition). Solid lines represent the median; dashed lines indicate the upper and lower quartiles. **** p < 0.0001 (n, o), and ** p = 0.0015 and *** p = 0.0002 (p) (one-way ANOVA with Tukey's multiple-comparison test). A.U., arbitrary units. Source data are provided as a Source Data file.

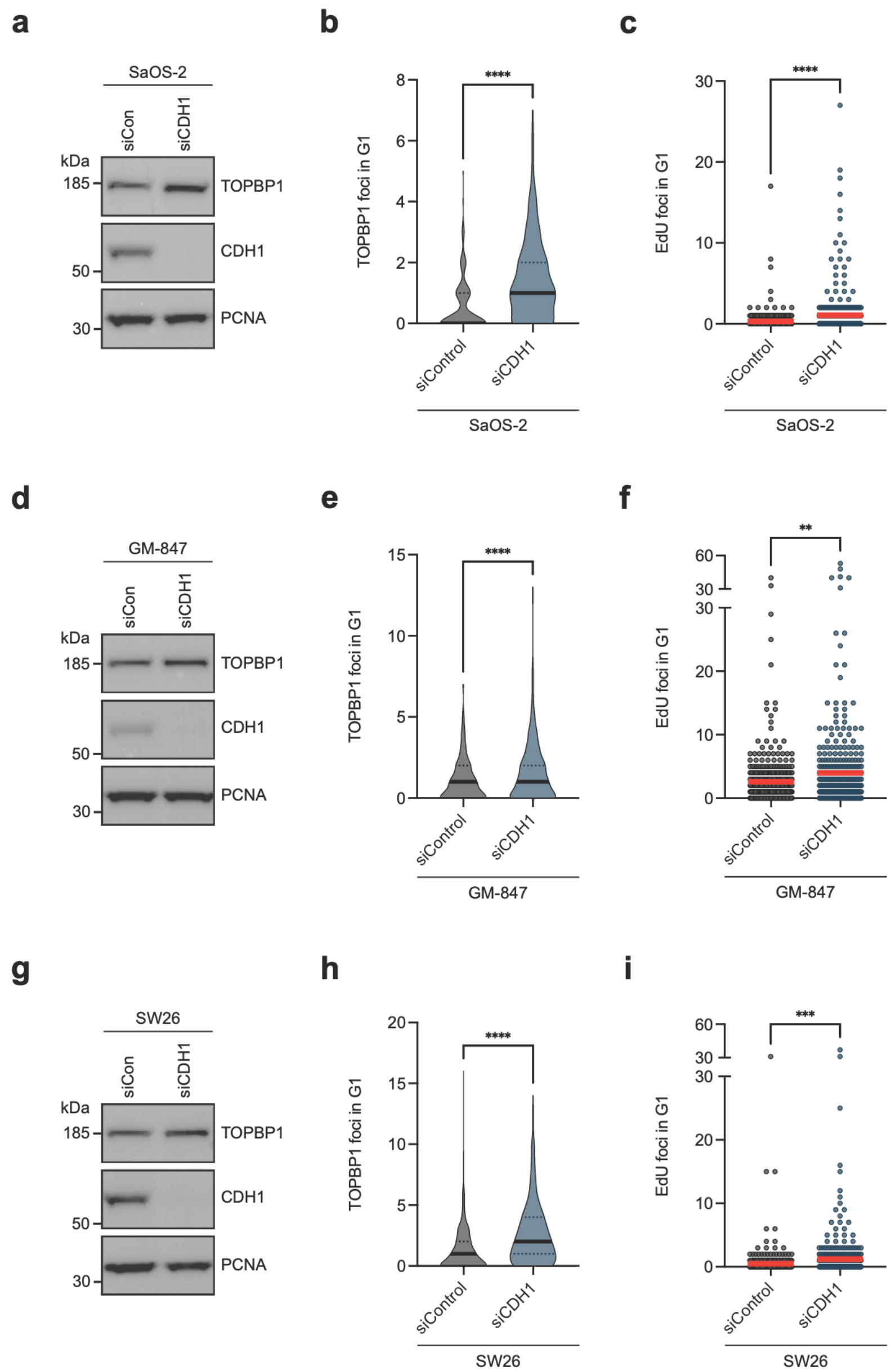

Supplementary Figure 11: APC/C^{CDH1} restrains post-MiDAS in multiple ALT-positive cancer cell lines. a Western blot analysis of TOPBP1, CDH1, and PCNA protein levels in whole-cell lysates of SaOS-2 cells following transfection with control or CDH1 siRNA. **b** Violin plot of TOPBP1 foci count per G1-phase SaOS-2 nucleus following transfection with control or CDH1 siRNA (n = 350 cells per condition). Solid lines represent the median; dashed lines indicate the upper and lower quartiles. **** $p < 0.0001$ (two-tailed unpaired t-test). **c** EdU foci count per G1-phase SaOS-2 nucleus following transfection with control or CDH1 siRNA. Scatterplots show n = 350 cells per condition. Red lines represent the mean. **** $p < 0.0001$ (two-tailed unpaired t-test). **d** Western blot analysis of TOPBP1, CDH1, and PCNA protein levels in whole-cell lysates of GM-847 cells following transfection with control or CDH1 siRNA. **e** Violin plot of TOPBP1 foci count per G1-phase GM-847 nucleus following transfection with control or CDH1 siRNA (n = 350 cells per condition). Solid lines represent the median; dashed lines indicate the upper and lower quartiles. **** $p < 0.0001$ (two-tailed unpaired t-test). **f** EdU foci count per G1-phase GM-847 nucleus following transfection with control or CDH1 siRNA. Scatterplots show n = 350 cells per condition. Red lines represent the mean. ** $p = 0.0011$ (two-tailed unpaired t-test). **g** Western blot analysis of TOPBP1, CDH1, and PCNA protein levels in whole-cell lysates of SW26 cells following transfection with control or CDH1 siRNA. **h** Violin plot of TOPBP1 foci count per G1-phase SW26 nucleus following transfection with control or CDH1 siRNA (n = 350 cells per condition). Solid lines represent the median; dashed lines indicate the upper and lower quartiles. **** $p < 0.0001$ (two-tailed unpaired t-test). **i** EdU foci count per G1-phase SW26 nucleus following transfection with control or CDH1 siRNA. Scatterplots show n = 350 cells per condition. Red lines represent the mean. *** $p = 0.0008$ (two-tailed unpaired t-test). Source data are provided as a Source Data file.

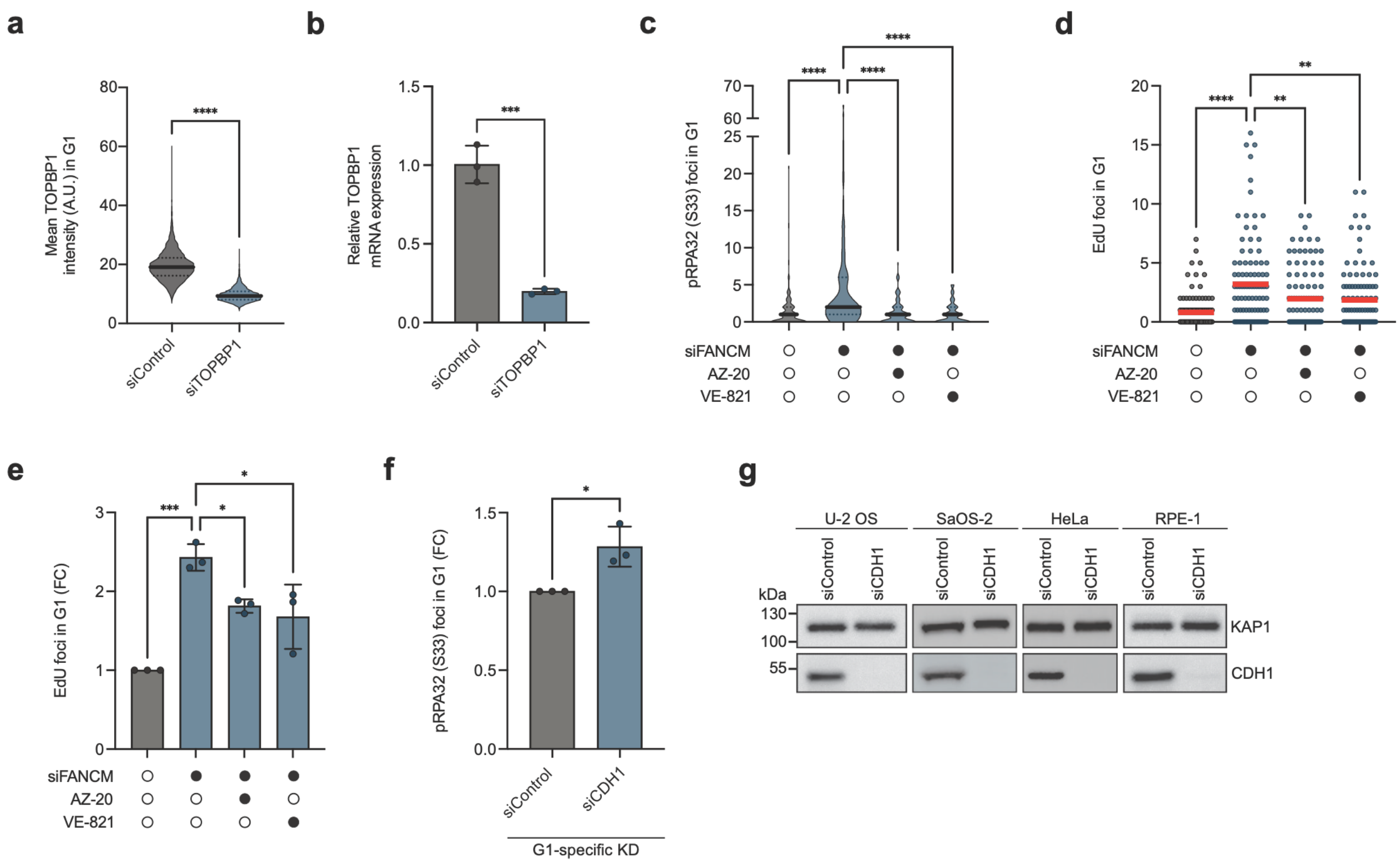

Supplementary Figure 12: APC/C^{CDH1} regulates TOPBP1 in the context of post-MiDAS in ALT-positive cancer cells. **a** Violin plot of mean TOPBP1 intensity (A.U.) per G1-phase U-2 OS nucleus following transfection with control or TOPBP1 siRNA (n = 4000 cells per condition). Solid lines represent the median; dashed lines indicate the upper and lower quartiles. **** $p < 0.0001$ (two-tailed unpaired t-test). **b** Relative TOPBP1 mRNA expression in U-2 OS cells following transfection with control or TOPBP1 siRNA. Individual values and means ± SD normalized to the siControl condition are shown. *** $p = 0.0003$ (two-tailed unpaired t-test). **c** Violin plot of pRPA32 (S33) foci count per G1-phase U-2 OS nucleus. Cells were transfected with control or FANCM siRNA, as indicated, and left untreated or treated with two independent ATR inhibitors (AZ-20 or VE-821, 20 μM) for 2 hours (n = 120 cells per condition). Solid lines represent the median; dashed lines indicate the upper and lower quartiles. **** $p < 0.0001$ (one-way ANOVA with Sidak's multiple-comparison test). **d** EdU foci count per G1-phase U-2 OS nucleus. Cells were transfected and treated as in (c). Scatterplots show n = 100 cells per condition. Red lines represent the mean. **** (siControl untreated vs. siFANCM untreated) $p < 0.0001$, ** (siFANCM untreated vs. siFANCM + AZ-20) $p = 0.0039$, and ** (siFANCM untreated vs. siFANCM + VE-821) $p = 0.0015$ (one-way ANOVA with Sidak's multiple-comparison test). **e** Normalized EdU foci count (FC) per G1-phase U-2 OS nucleus. Cells were transfected and treated as in (c). Means ± SD of three independent experiments normalized to the untreated siControl condition are shown. *** (siControl untreated vs. siFANCM untreated) $p = 0.0002$, * (siFANCM untreated vs. siFANCM + AZ-20) $p = 0.0298$, and * (siFANCM untreated vs. siFANCM + VE-821) $p = 0.0103$ (one-way ANOVA with Sidak's multiple-comparison test). **f** Normalized pRPA32 (S33) foci count (FC) per G1-phase U-2 OS nucleus following G1-specific transfection with control or CDH1 siRNA. Means ± SD of three independent experiments normalized to the siControl condition are shown (n_1 = 900, n_2 = 350, and n_3 = 300 cells per experiment). * $p = 0.0181$ (two-tailed unpaired t-test). **g** Western blot analysis of CDH1 and KAP1 protein levels in whole-cell lysates from U-2 OS, SaOS-2, HeLa, and RPE-1 cells following transfection with control or CDH1 siRNA. The knockdown validation by Western blot was performed once. A.U., arbitrary units. Source data are provided as a Source Data file.

Primer name	Primer sequence (5'-3')	Source
TurboID-RPA2_mCh_Cry2_del_HA-stop_fw	TACCCATACGACGTACCAGATTACGCTTAACCCGGGATCCACCGGTCGCCACCG	This study
TurboID-RPA2_mCh_Cry2_del_rev	TTCTGCATCTGTGGATTTAAAATGG	This study
pcDNA31_to_TurboID-RPA2-GA_fw	GACTCACTATAGGGAGACCCAAGCTGGCTAGCATGAAAGACAATACTGTGCCTCTGAAGC	This study
pcDNA31_to_TurboID-RPA2_GA_rev	GGACTAGTGGATCCGAGCTCGGTACCAAGCTTTTAAGCGTAATCTGGTACGTCGTATGG	This study
pcDNA3.1_lin_fw	TAAAAGCTTGGTACCGAGCTCGG	This study
pcDNA3.1_lin_rv	CATGCTAGCCAGCTTGGGTCTC	This study

Supplementary Table 1: List of primer sequences used for cloning.

Primary antibodies	Manufacturer	Identifier	Dilution
Rabbit polyclonal anti-APC1	Abcam	ab133397	WB 1:500
Rabbit polyclonal anti-BLM	Abcam	ab2179	IF 1:1000
Rat monoclonal anti-BrdU (BU1/75 (ICR1))	Abcam	ab6326	IF 1:500
Mouse monoclonal anti-CDC27/APC3 (AF3.1)	Santa Cruz	sc-9972	WB 1:200
Human polyclonal anti-CREST	Antibodies Incorporated	15-234	IF 1:500
Mouse monoclonal anti-Cyclin A (B-8)	Santa Cruz	sc-271682	WB 1:1000
Rabbit monoclonal anti-Cyclin A2 (EPR17351)	Abcam	ab181591	IF 1:500
Rabbit polyclonal anti-FANCD2	Novus Biologicals	NB100-182	IF 1:100
Mouse monoclonal anti-fzr/CDH1 (DCS-266)	Santa Cruz	sc-56312	WB 1:100
Mouse monoclonal anti-HA.11	Biolegend	MMS-101P	IF 1:500
Rabbit polyclonal anti-KAP1	Bethyl Laboratories	A300-274A	WB 1:2000
Rabbit polyclonal anti-Lamin A/C	Cell Signaling	2032	WB 1:1000
Goat polyclonal anti-MCM2 (N-19)	Santa Cruz	sc-9839	IF 1:100
Mouse monoclonal anti-Mono- and polyubiquitinylated conjugates (UBCJ2)	Enzo Life Sciences	ENZ-ABS840	IF 1:1000
Mouse monoclonal anti-Mono- and polyubiquitinylated conjugates (FK2)	Enzo Life Sciences	BML-PW8810-0500	IF 1:1000
Mouse monoclonal anti-PCNA (PC10)	Santa Cruz	sc-56	IF 1:500, WB 1:2000
Mouse monoclonal anti-PML (PG-M3)	Santa Cruz	sc-966	IF 1:200
Rabbit polyclonal anti-Phospho-RPA32 (Ser33)	Bethyl Laboratories	A300-246A	IF 1:500
Mouse monoclonal anti-RPA32/RPA2 (9H8)	Abcam	ab2175	IF 1:300, WB 1:500
Rabbit monoclonal anti-RPA70 (EPR3472)	Abcam	ab79398	IF 1:500, WB 1:500
Streptavidin-HRP antibody	Abcam	ab7403	WB 1:3700
Rabbit monoclonal anti-SUMO1 (Y299)	Abcam	ab32058	IF 1:200
Rabbit monoclonal anti-SUMO2/3 (18H8)	Cell Signaling	4971	IF 1:100
Rabbit polyclonal anti-TopBP1	Bethyl Laboratories	A300-111A	IF 1:1000, WB 1:200
Mouse monoclonal anti-TRF2 (4A794)	Abcam	ab13579	IF 1:500
Rabbit polyclonal anti-TRF2	Novus Biologicals	NB110-57130	IF 1:200
Mouse monoclonal anti-α-Tubulin (DM1A)	Sigma-Aldrich	T6199	WB 1:5000
Mouse monoclonal anti-H2A.X Phospho (Ser139) (2F3)	Biolegend	613402	IF 1:1000

Secondary antibodies	Manufacturer	Identifier	Dilution
Alexa Fluor 488 Goat Anti-Mouse	Thermo Fisher Scientific	A11029	IF 1:500
Alexa Fluor 488 Goat Anti-Rabbit	Thermo Fisher Scientific	A11034	IF 1:500
Alexa Fluor 488 Goat Anti-Human	Thermo Fisher Scientific	A11013	IF 1:250
Alexa Fluor 488 Donkey Anti-Goat	Thermo Fisher Scientific	A11055	IF 1:500
Alexa Fluor 488 Donkey Anti-Rat	Thermo Fisher Scientific	A21208	IF 1:500
Alexa Fluor 568 Goat Anti-Mouse	Thermo Fisher Scientific	A11031	IF 1:500
Alexa Fluor 568 Goat Anti-Rabbit	Thermo Fisher Scientific	A11036	IF 1:500
Alexa Fluor 647 Goat Anti-Mouse	Thermo Fisher Scientific	A21235	IF 1:500
HRP Horse Anti-Mouse IgG Antibody (H+L)	Vector Laboratories	PI-2000	WB 1:10000
HRP Goat Anti-Rabbit IgG Antibody (H+L)	Vector Laboratories	PI-1000	WB 1:10000
HRP Goat Anti-Mouse light-chain Antibody	Sigma-Aldrich	AP200P	WB 1:5000
HRP Mouse Anti-Rabbit light-chain Antibody	Sigma-Aldrich	MAB201P	WB 1:5000

Supplementary Table 2: List of antibodies used for immunofluorescence staining and Western blotting.

Primer	Sequence (5' to 3')	Source
BLM fwd	GCTCAGGAAAGTGACTCTCTG	This study, validated by knockdown in Supplementary Figure 5g
BLM rev	TTCTTCTGGAGAAGGTGGAAC	This study, validated by knockdown in Supplementary Figure 5g
CDC20 fwd	TCCTGTCCAGTGGTTCACGTTC	This study, validated by knockdown in Supplementary Figure 10d
CDC20 rev	CATTACCACCACTGGCCAAATG	This study, validated by knockdown in Supplementary Figure 10d
FANCD2 fwd	TACTGCAGCCTGCCATTATC	This study, validated by knockdown in Supplementary Figure 7k
FANCD2 rev	GACTGACAATGAGTCGAGGTATG	This study, validated by knockdown in Supplementary Figure 7k
FANCM fwd	GAGACACGAAGAGGGAGAATGC	This study, validated by knockdown in Supplementary Figure 5d
FANCM rev	ATCACTGAGGATGAACTGCCTTG	This study, validated by knockdown in Supplementary Figure 5d
FZR1 (CDH1) fwd	GCACGCCAACGAGCTGGTGAGC	This study, validated by knockdown in Supplementary Figure 10c
FZR1 (CDH1) rev	CAGACACAGACTCCCACTTTACC	This study, validated by knockdown in Supplementary Figure 10c
MCM2 fwd	GGTCAACATGGAGGAGACCATCTATC	This study, validated by knockdown in Supplementary Figure 7c
MCM2 rev	GCCAGTCAGCTCTATCTCGTCTCC	This study, validated by knockdown in Supplementary Figure 7c
POLD3 fwd	ACCAACAAGGAAACGAAAACAGA	PMID: 35440629; validated by knockdown in Supplementary Figure 5h
POLD3 rev	GGTTCCGTGACAGACACTGTA	PMID: 35440629; validated by knockdown in Supplementary Figure 5h
RAD18 fwd	CTGTATGCATGGGACAGGAAGAT	This study, validated by knockdown in Supplementary Figure 7h
RAD18 rev	GATGTCTGAACTGGAACTATTGCATG	This study, validated by knockdown in Supplementary Figure 7h
RMI1 fwd	GTCAGCCTGCATACTCCCAGATAC	PMID: 41614397; validated by knockdown in Supplementary Figure 5f
RMI1 rev	GCAGCATCAACATTCGTGAAGGC	PMID: 41614397; validated by knockdown in Supplementary Figure 5f
SLX4 fwd	AACCCACACCTGAGTGATGTCC	This study, validated by knockdown in Supplementary Figure 5b
SLX4 rev	TCCTCTACAGCGGAGAAGCCTT	This study, validated by knockdown in Supplementary Figure 5b
TOP3A fwd	ATGAGGCGGAGAGAAGGACT	PMID: 35920001; validated by knockdown in Supplementary Figure 5e
TOP3A rev	CTGCATCTGGAAATCATGAGCC	PMID: 35920001; validated by knockdown in Supplementary Figure 5e
TOPBP1 fwd	GCAGAACTGTTGCGGATTATG	This study, validated by knockdown in Supplementary Figure 12b
TOPBP1 rev	GCAAGTAACCAGCCATGTATTT	This study, validated by knockdown in Supplementary Figure 12b
WRN fwd	TGCTAGTGATTGCTCTTTCCTG	PMID: 42247504; validated by knockdown in Supplementary Figure 5c
WRN rev	CTTTGCCAAGTTTCCCTCTATTG	PMID: 42247504; validated by knockdown in Supplementary Figure 5c

Supplementary Table 3: List of primer sequences used for qPCR.
