# Supplementary material for: Characterization of post-mitotic DNA synthesis and its regulation in human cells
Source: Nat Commun. 2026 Sep 28;17:11186. doi: 10.1038/s41467-026-78035-5 (PMC13713988; doi:10.1038/s41467-026-78035-5)
Supplement: Supplementary file 4 [file 41467_2026_78035_MOESM2_ESM.pdf]

Characterization of post-mitotic DNA synthesis and its regulation in human cells

Vornberger et al. Nature Communications (2026)

Description of Additional Supplementary Files

File name: Supplementary Data 1
Description: List of siRNAs included in the siRNA screen in G1-synchronized cells presented in Figure 3.

File name: Supplementary Data 2
Description: Significantly enriched RPA70 interactors identified by RPA70 IP-MS from quadruplicate whole-cell protein extract samples presented in Figure 6 and Supplementary Figure 8. Statistical analysis of interactome data was performed using SAINT, with significant RPA70 interactors defined by a Bayesian false discovery rate (BFDR) ≤ 0.05 and an empirical fold change (EFC) ≥ 2 compared with negative IgG controls.

File name: Supplementary Data 3
Description: Significantly enriched RPA70 interactors identified by RPA70 IP-MS from quadruplicate chromatin-bound protein extract samples presented in Figure 6 and Supplementary Figure 8. Statistical analysis of interactome data was performed using SAINT, with significant RPA70 interactors defined by a BFDR ≤ 0.05 and an EFC ≥ 2 compared with IgG controls.

File name: Supplementary Data 4
Description: Significantly enriched RPA32 interactors identified by TurboID-RPA32 MS from duplicate nuclear protein extract samples presented in Supplementary Figure 9. Statistical analysis of interactome data was performed using SAINTexpress, with significant RPA32 interactors defined by a BFDR ≤ 0.05 and an EFC ≥ 1.9 compared with unlabeled controls.

File name: Supplementary Data 5
Description: List of siRNAs included in the siRNA validation screen of RPA70-IP LC-MS hits presented in Supplementary Figure 9.

File name: Supplementary Movie 1
Description: Time-lapse microscopy of untreated U-2 OS H2B-GFP cells over 24 hours with images acquired at 15-minute intervals.

File name: Supplementary Movie 2
Description: Time-lapse microscopy of U-2 OS H2B-GFP cells treated with Palbociclib (1 μM) for 72 hours and imaged during the final 24 hours at 15-minute intervals.

File name: Supplementary Movie 3
Description: Time-lapse microscopy of U-2 OS H2B-GFP cells treated with Palbociclib (1 μM) for 72 hours, with colcemid (0.1 μg/ml) added during the last 48 hours, and imaged during the final 24 hours at 15-minute intervals.
